# Diastereoselective Synthesis of Spirocyclopropanes under Mild Conditions via Formal [2 + 1] Cycloadditions Using 2,3-Dioxo-4-benzylidene-pyrrolidines

**DOI:** 10.3390/molecules22020328

**Published:** 2017-02-22

**Authors:** Yi Li, Qing-Zhu Li, Li Huang, Hong Liang, Kai-Chuan Yang, Hai-Jun Leng, Yue Liu, Xu-Dong Shen, Xiao-Jun Gou, Jun-Long Li

**Affiliations:** Antibiotics Research and Re-evaluation Key Laboratory of Sichuan Province, Sichuan Industrial Institute of Antibiotics, Chengdu University, Chengdu 610052, China; lee90918@outlook.com (Y.L.); lilililylily@outlook.com (L.H.); Lianghong@mail.cdu.edu.cn (H.L.); Kaichuanyang@163.com (K.-C.Y.); lnavy@126.com (H.-J.L.); lucindalau1225@sina.com (Y.L.); shenxudong@cdu.edu.cn (X.-D.S.)

**Keywords:** spirocyclopropanes, diastereoselective synthesis, catalyst free, sulfur ylide

## Abstract

A highly diastereoselective cyclopropanation of cyclic enones with sulfur ylides was developed under catalyst-free conditions, producing multifunctional spirocyclopropanes in generally excellent yields (up to 99% yield and >99:1 d.r.). The asymmetric version of this method was realized by using an easily available chiral sulfur ylide, affording products with moderate to good stereoselectivity.

## 1. Introduction

**Figure 1:**
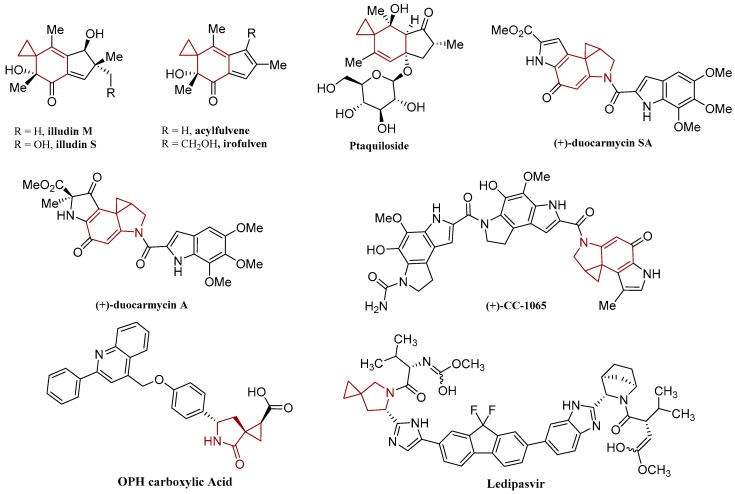
Selected products containing spirocyclopropanane possess biological activities.

Spirocycles are significant structures found in various natural products and many potent synthetic drug candidates [1,2,3]. Due to their important physiological functions, the synthesis of spirocycles has become an attractive target in organic chemistry, especially in recent years [4,5,6]. Among various spirocycles, spirocyclopropanes have shown high potential pharmaceutical activities (Figure 1). For example, the illudins [7,8], sesquiterpene secondary metabolites of basidiomycetes, have demonstrated activity against cancer; acylfulvene and irofulven [9,10], which are derived from illudin via a semisynthetic approach, also show anti-tumor bioactivity. Ptaquiloside [11], a toxic derivative from bracken, is recently reported to depress tumor-infiltration in HPV-16 transgenic mice. The natural products CC-1065, duocarmycin A and AS [12,13] were identified as strong anticancer drug candidates as well. Recently, many spirocyclopropanes possessing a pyrrolidin-2-one moiety have been developed into useful drug candidates. For instance, ledipasvir [14,15], a drug developed by Gilead Sciences, is an effective inhibitor of the hepatitis C virus. Other compounds reported by Berman et al. [16] also exhibited biological activities; for example, the OPH carboxylic acid can affect the function of disintegrin and metalloproteinase domain-containing proteins.

Meanwhile, cyclopropanes can also be applied as versatile units for the construction of other frameworks due to their unique combination of reactivity and structural properties [17,18,19,20]. For instance, the ring-enlargement reactions of cyclopropanes with nucleophiles, such as amines, alcohols, and carboxylic acids, are efficient pathways to various heterocycles [21,22,23,24,25,26,27]. Consequently, numerous efforts have been devoted to the formation of three-membered carbocyclic rings during the last few decades [28,29,30,31,32,33,34,35,36]. The reactions of carbenoids with alkenes such as the Simmons–Smith cyclo-propanation involving organozinc carbenes [37,38,39,40], or the addition of carbenes, generated from diazo compounds in the presence of transition metals, to double bonds [41,42,43,44] are the most significant and useful classical methods for the construction of cyclopropanes. In addition, base-promoted cyclopropanations between α-halogenated compounds and electron-deficient olefins [45,46,47] are also reliable approaches to cyclopropanes. However, these methods often require the use of metals or harsh conditions.

The cyclopropanation of electron-deficient olefins and ylides, including sulfonium [48,49,50,51,52,53,54,55], telluronium [56,57], arsonium [58,59,60,61], and ammonium ylides [62,63,64,65], represents one of the most efficient and straightforward strategies to construct cyclopropane-containing frameworks. Among them, sulfonium ylides, as well-developed active units, can also react with cyclic electron-withdrawing alkenes to access synthetically challenging spirocyclopropanes [51,52,53,54,55]. A great number of studies have been directed to the cyclopropanation with sulfonium ylides; however, harsh conditions such as strong base were usually required, which has limited the structural diversity of cyclopropane scaffolds as well as restricted functional group tolerance. Therefore, the demand on exploring cyclopropanation chemistry under mild conditions and further expanding the structural generality is still highly desirable. Recently, we developed some convenient synthetic strategies directly toward heterocyclic compounds bearing the pyrrolidin-2-one moiety by using 2,3-dioxobenzylidenepyrrolidine, a highly reactive cyclic enone [66,67]. Considering the potential capacity of this enone to serve as an electron-deficient alkene, we report herein an efficient catalyst-free cyclopropanation with 2,3-dioxopyrrolidine and sulfur ylides which leads to the diastereoselective synthesis of spirocyclopropanes.

## 2. Results

In our initial research, the reaction of readily available cyclic enone **1a** and sulfur ylide **2a** was carried out in dichloromethane (DCM) at room temperature (Table 1). We were pleased to find that, under these conditions, **1a** underwent the desired cyclopropanation with sulfur ylide **2a** giving spirocyclopropane **3a** in good yield (86%) and with promising diastereoselectivity (d.r. = 93:7, Entry 1). Encouraged by this result, we proceeded to optimize the reaction by evaluating the effect of solvents. As outlined in Table 1, a series of solvents were examined (Entries 2–9) and 1,4-dioxane provided the best yield and diastereoselectivity (Entry 5). Meanwhile, since the reactions worked quite well at room temperature, a screening study of temperature effects was avoided. Therefore, 1,4-dioxane as the solvent at room temperature were determined as the optimal reaction conditions.

**Scheme 1:**
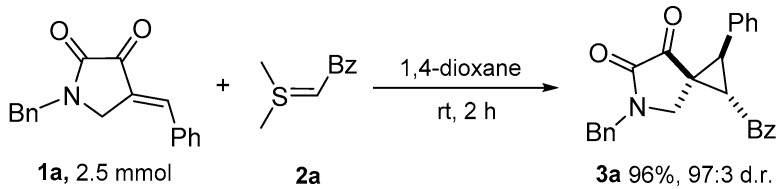
Scale-up experiment of the cyclopropanation reaction.

After the optimal conditions of the reaction were established, we then investigated the generality of this reaction with a variety of 2,3-dioxopyrrolidine derivatives **1** as substrates (Table 2). By using different cyclic enones **1** bearing various kinds of substituents on phenyl ring, the reactions can finished rapidly in 2 h to afford the corresponding spirocyclopropane **3a**–**3n** in good to excellent yields with satisfactory diastereoselectivity (Entries 1–14). The reactions were also suitable for enone substrates with polycyclic or heteroaromatics, such as 1-naphthyl and thienyl rings (Entries 15 and 16) (For details, please see Supplementary File Part 2). Furthermore, the practicality of this methodology was illustrated by a scaled up reaction: 2.5 mmol of cyclic enone **1a** was treated with 2.5 mmol of sulfur ylide **2a** under the optimal conditions in 1,4-dioxane. The desired product **3a** was obtained in excellent yield with outstanding diastereoselectivity (96% yield and 97:3 d.r., see Scheme 1).

On the other hand, the amide functional group commonly exists in many bioactive natural products and medicinal molecules [68,69]. With the intention of preparing amide-containing spirocyclopropanes, benzyl or PMB substituted amidic sulfonium salts **4a** and **4b** were prepared for further investigation. A simple attempt to cyclopropanate enone **1a** with an amidic sulfur ylide of **4a** was not successful. However, to our satisfaction, the reactions of cyclic enones **1** and amidic sulfonium salt **4** in the presence of 1,1,3,3-tetramethylguanidine (TMG) in 1,4-dioxone at room temperature proceeded efficiently to access the desired products within 4 h. As shown in Table 3, several cyclic enones bearing different substituents on the phenyl ring smoothly reacted with sulfonium salts **4a** or **4b**, to obtain an array of amide-containing spirocyclopropanes with good results (Entries 1–6). Moreover, the 1-naphthyl and 2-thienyl cyclic enones showed lower reactivity but also gave the corresponding products in good yields with excellent diastereoselectivity, albeit with longer reaction times (Entries 7–10) (For details, please see Supplementary File Part 3).

Encouraged by the selective reaction outcomes of amide substrates **4a** and **4b**, further investigation of asymmetric synthesis of chiral spirocyclopropanes were evaluated by introducing a chirality-inducing group on the amidic sulfonium salt. As outlined in Table 4, the easily available chiral *N*-phenylethyl sulfur ylide precursor **4c** was utilized to react with cyclic enone **1a** in 1,4-dioxane at room temperature, which was promoted by a series of organic and inorganic bases. Chiral spirocyclopropane **6a** was generally obtained in excellent yields with moderate diastereoselectivity (Entries 1–5), and TMG was demonstrated to be the optimal base. Notably, both the two diastereoisomers which are enantiopure products could be easily obtained by simple flash chromatography.

Having established the optimized conditions for the asymmetric cyclopropanation, we then investigated the performance of a variety of cyclic enone **1** in the reaction system promoted by TMG. The results are summarized in Table 5.

**Figure 2:**
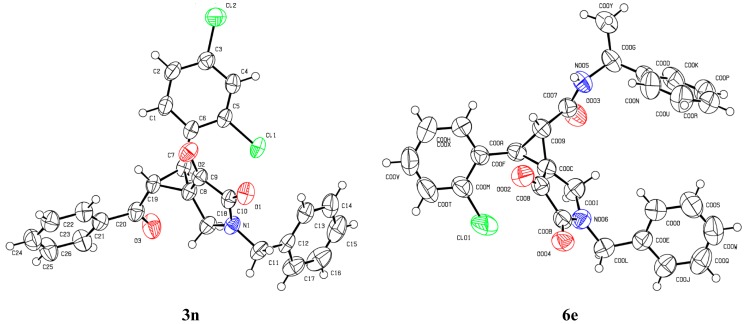
Single crystal X-ray diffraction analysis of products **3n** and **6e**.

Enone substrates with electron-withdrawing or electron-donating groups on the phenyl ring were well tolerated, delivering the desired products **6a**–**6g** in high yields with moderate to good diastereoselectivity (Entries 1–6). Furthermore, enones bearing diverse aryl or heteroaryl groups, such as 1-naphthyl, 2-naphthyl and 2-thienyl were also tolerated to produce **6h**–**6j** with similar results (Entries 7–9) (For details, please see Supplementary File Part 4). Moreover, structural correctness and the absolute configuration of the spirocyclopropanes were confirmed by X-ray diffraction analysis of the representative products **3n** and the enantiopure **6e** (Figure 2) [70]. (For details, please see Supplementary File Parts 5 and 6).

## 3. Materials and Methods

### 3.1. General Information

Commercial reagents and solvents were obtained from Adamas-beta (Shanghai, China), Aldrich Chemical Co. (Darmstadt, Germany), Alfa Aesar (Shanghai, China), Macklin (Shanghai, China) and Energy Chemical (Shanghai, China) and used as received with the following exceptions: THF, and toluene were purified by refluxing over Na-benzophenone under positive argon pressure followed by distillation [71,72,73]. The enone substrates were prepared according to literature procedure [74]. 

Proton nuclear magnetic resonance (^1^H-NMR, 400 MHz) and carbon-13 nuclear magnetic resonance (^13^C-NMR, 100 MHz) spectra were recorded in CDCl_3_ on an AV 400 MHz spectrometer (Bruker, Billerica, MA, USA). Proton chemical shifts are reported in parts per million (δ scale), and are referenced using residual protons in the NMR solvent (CHCl_3_ δ 7.26). Carbon chemical shifts are reported in parts per million (δ scale), and are referenced using the carbon resonances of the solvent (CDCl_3_: triplet centered at δ 77.01). High resolution mass spectra (HRMS) were recorded on a SYNAPT G2 system (Waters, Milford, CT, USA) using an electrospray (ESI) ionization source.

### 3.2. Synthesis

#### 3.2.1. General Procedure for the Synthesis of Multi-Substituted Spirocyclopropane **3**

A dried glass tube was charged with cyclic enone **1** (0.1 mmol) and sulfur ylide **2** (0.1 mmol) in 1,4-dioxane (0.5 M, 2 mL). The reaction vessel was sealed with a Teflon cap and stirred at room temperature for about 2 h. When the reaction was complete, the reaction mixture was concentrated and the residue was purified by flash chromatography on silica gel (petroleum ether/ethyl acetate) to afford the corresponding spirocyclopropane **3**, which was dried under vacuum oven and further analyzed by ^1^H-NMR, ^13^C-HMR, HRMS, etc.

*1-Benzoyl-5-benzyl-2-phenyl-5-azaspiro[2.4]heptane-6,7-dione* (**3a**). Purification of the crude product via flash chromatography on silica gel (petroleum ether/ethyl acetate = 4:1) to afford the corresponding **3a** as a white solid with 98:2 dr, 92% yield. ^1^H-NMR δ 8.05–7.99 (m, 2H), 7.69–7.60 (m, 1H), 7.52 (t, *J* = 7.7 Hz, 2H), 7.41–7.28 (m, 7H), 7.28–7.21 (m, 3H), 4.84–4.66 (dd, *J* = 14.4 Hz, 2H), 4.23 (d, *J* = 7.2 Hz, 1H), 3.88 (d, *J* = 12.2 Hz, 1H), 3.69 (d, *J* = 12.1 Hz, 1H), 3.59 (d, *J* = 7.2 Hz, 1H); ^13^C-NMR δ 194.9, 193.4, 159.2, 136.6, 134.5, 134.2, 131.7, 129.0, 129.0, 129.0, 128.6, 128.4, 128.4, 128.4, 128.1, 48.7, 47.3, 44.3, 41.1, 36.6; HRMS: *m*/*z* calculated for C_21_H_17_N_3_O_2_Na^+^: 366.1218, found: 366.1227.

*1-Benzoyl-5-benzyl-2-(m-tolyl)-5-azaspiro[2.4]heptane-6,7-dione* (**3b**). Purification of the crude product via flash chromatography on silica gel (petroleum ether/ethyl acetate = 4:1) to afford the corresponding **3b** as a white solid with 92:8 d.r., 83% yield. ^1^H-NMR δ (ppm) 8.06–7.99 (m, 2H), 7.68–7.61 (m, 1H), 7.52 (t, *J* = 7.8 Hz, 2H), 7.40–7.28 (m, 5H), 7.20 (t, *J* = 8.0 Hz, 1H), 7.06 (m, 3H), 4.74 (q, *J* = 14.4 Hz, 2H), 4.22 (d, *J* = 7.2 Hz, 1H), 3.87 (d, *J* = 12.4 Hz, 1H), 3.68 (d, *J* = 12.4 Hz, 1H), 3.55 (d, *J* = 7.2 Hz, 1H), 2.33 (s, 3H); ^13^C-NMR δ (ppm): 194.9, 193.4, 159.3, 138.1, 136.6, 134.5, 134.2, 131.6, 129.6, 129.0, 129.0, 128.9, 128.7, 128.6, 128.4, 128.3, 126.0, 48.7, 47.4, 44.4, 41.1, 36.6, 21.3; HRMS: *m*/*z* calculated for C_27_H_23_NO_3_Na^+^: 432.1576, found: 432.1573.

*1-Benzoyl-5-benzyl-2-(p-tolyl)-5-azaspiro[2.4]heptane-6,7-dione* (**3c**). Purification of the crude product via flash chromatography on silica gel (petroleum ether/ethyl acetate = 4:1) to afford the corresponding **3c** as a white solid with 98:2 d.r., 93% yield; ^1^H-NMR δ (ppm) 8.10–7.86 (m, 2H), 7.68–7.60 (m, 1H), 7.52 (t, *J* = 7.8 Hz, 2H), 7.41–7.28 (m, 5H), 7.18–7.04 (m, 4H), 4.78 (d, *J* = 14.4 Hz, 1H), 4.69 (d, *J* = 14.4 Hz, 1H), 4.20 (d, *J* = 7.2 Hz, 1H), 3.87 (d, *J* = 12.0 Hz, 1H), 3.68 (d, *J* = 12.0 Hz, 1H), 3.55 (d, *J* = 7.2 Hz, 1H), 2.31 (s, 3H); ^13^C-NMR δ (ppm) 195.0, 193.4, 159.3, 137.9, 136.6, 134.5, 134.2, 129.1, 129.0, 129.0, 128.8, 128.7, 128.6, 128.6, 128.3, 48.7, 47.3, 44.3, 41.2, 36.6, 21.1; HRMS: *m*/*z* calculated for C_27_H_23_NO_3_Na^+^: 432.1576, found: 432.1579.

*1-Benzoyl-5-benzyl-2-(2-methoxyphenyl)-5-azaspiro[2.4]heptane-6,7-dione* (**3d**). Purification of the crude product via flash chromatography on silica gel (petroleum ether/ethyl acetate = 4:1) to afford the corresponding **3d** as a white solid with 92:8 d.r., 83% yield; ^1^H-NMR δ (ppm) 8.11–7.99 (m, 2H), 7.70–7.61 (m, 1H), 7.57–7.48 (m, 2H), 7.43–7.31 (m, 5H), 7.30–7.21 (m, 2H), 6.96 (m, 1H), 6.78 (dd, *J* = 8.4, 0.8 Hz, 1H), 5.01 (d, *J* = 14.4 Hz, 1H), 4.54 (d, *J* = 14.4 Hz, 1H), 4.04 (d, *J* = 7.0 Hz, 1H), 3.95 (d, *J* = 11.8 Hz, 1H), 3.69 (d, *J* = 11.8 Hz, 1H), 3.62 (s, 3H); ^13^C-NMR δ (ppm): 195.2, 192.6, 159.8, 157.0, 136.8, 135.0, 134.1, 130.3, 130.1, 129.4, 129.0, 128.6, 128.4, 128.3, 121.2, 120.7, 110.3, 55.1, 48.5, 47.4, 39.8, 39.1, 36.7; HRMS: *m*/*z* calculated for C_27_H_23_NO_4_Na^+^: 448.1525, found: 448.1529.

*1-Benzoyl-5-benzyl-2-(3-methoxyphenyl)-5-azaspiro[2.4]heptane-6,7-dione* (**3e**). Purification of the crude product via flash chromatography on silica gel (petroleum ether/ethyl acetate = 4:1) to afford the corresponding **3e** as a white solid with 97:3 d.r., 91% yield; ^1^H-NMR δ (ppm) 8.05–7.97 (m, 2H), 7.69–7.60 (m, 1H), 7.52 (t, *J* = 7.6 Hz, 2H), 7.41–7.27 (m, 5H), 7.22 (m, 1H), 6.87–6.75 (m, 3H), 4.79 (d, *J* = 14.4 Hz, 1H), 4.69 (d, *J* = 14.4 Hz, 1H), 4.21 (d, *J* = 7.2 Hz, 1H), 3.87 (d, *J* = 12.0 Hz, 1H), 3.79 (s, 3H), 3.68 (d, *J* = 12.0 Hz, 1H), 3.55 (d, *J* = 7.2 Hz, 1H); ^13^C-NMR δ (ppm): 194.8, 193.4, 159.5, 159.2, 136.6, 134.5, 134.2, 133.2, 129.4, 129.0, 129.0, 128.7, 128.5, 128.4, 121.3, 115.1, 113.2, 55.2, 48.7, 47.3, 44.2, 41.1, 36.6; HRMS: *m*/*z* calculated for C_27_H_23_NO_4_Na^+^: 448.1525, found: 448.1523.

*1-Benzoyl-5-benzyl-2-(4-methoxyphenyl)-5-azaspiro[2.4]heptane-6,7-dione* (**3f**). Purification of the crude product via flash chromatography on silica gel (petroleum ether/ethyl acetate = 4:1) to afford the corresponding **3f** as a white solid with 96:4 d.r., 94% yield; ^1^H-NMR δ (ppm) 8.06–7.95 (m, 2H), 7.69–7.60 (m, 1H), 7.52 (t, *J* = 7.6 Hz, 2H), 7.40–7.28 (m, 5H), 7.22–7.13 (m, 2H), 6.89–6.78 (m, 2H), 4.78 (d, *J* = 14.4 Hz, 1H), 4.69 (d, *J* = 14.4 Hz, 1H), 4.19 (d, *J* = 7.2 Hz, 1H), 3.86 (d, *J* = 12.4 Hz, 1H), 3.78 (s, 3H), 3.68 (d, *J* = 12.0 Hz, 1H), 3.54 (d, *J* = 7.2 Hz, 1H); ^13^C-NMR δ (ppm) 195.0, 193.4, 159.4, 136.6, 134.5, 134.2, 130.1, 129.1, 129.0, 128.6, 128.4, 128.4, 128.0, 123.6, 113.9, 55.3, 48.7, 47.4, 44.2, 41.3, 36.8; HRMS: *m*/*z* calculated for C_27_H_23_NO_4_Na^+^: 448.1525, found: 448.1521.

*1-Benzoyl-5-benzyl-2-(4-fluorophenyl)-5-azaspiro[2.4]heptane-6,7-dione* (**3g**). Purification of the crude product via flash chromatography on silica gel (petroleum ether/ethyl acetate = 4:1) to afford the corresponding **3g** as a white solid with 96:4 d.r., 88% yield; ^1^H-NMR δ (ppm) 8.05–7.96 (m, 2H), 7.66 (t, *J* = 7.6 Hz, 1H), 7.53 (t, *J* = 7.7 Hz, 2H), 7.35 (m, 3H), 7.30 (m, 2H), 7.27–7.17 (m, 2H), 7.00 (t, *J* = 8.6 Hz, 2H), 4.79 (d, *J* = 14.4 Hz, 1H), 4.68 (d, *J* = 14.4 Hz, 1H), 4.17 (d, *J* = 7.2 Hz, 1H), 3.86 (d, *J* = 12.0 Hz, 1H), 3.67 (d, *J* = 12.0 Hz, 1H), 3.55 (d, *J* = 7.2 Hz, 1H); ^13^C-NMR δ (ppm) 194.6, 193.5, 159.1, 136.5, 134.4, 134.3, 130.7, 130.6, 129.1, 129.0, 128.6, 128.4, 128.4, 115.5, 115.3, 48.7, 47.2, 43.3, 41.0, 36.8; HRMS: *m*/*z* calculated for C_26_H_20_FNO_3_Na^+^: 436.1325, found: 436.1322.

*1-Benzoyl-5-benzyl-2-(2-chlorophenyl)-5-azaspiro[2.4]heptane-6,7-dione* (**3h**). Purification of the crude product via flash chromatography on silica gel (petroleum ether/ethyl acetate = 4:1) to afford the corresponding **3h** as a white solid with 97:3 d.r., 98% yield; ^1^H-NMR δ (ppm) 8.15–7.95 (m, 2H), 7.77–7.60 (m, 1H), 7.54 (t, *J* = 7.6 Hz, 2H), 7.41–7.29 (m, 7H), 7.27–7.20 (m, 2H), 5.06 (d, *J* = 14.4 Hz, 1H), 4.45 (d, *J* = 14.4 Hz, 1H), 4.16 (d, *J* = 6.8 Hz, 1H), 3.94 (d, *J* = 12.0 Hz, 1H), 3.67 (d, *J* = 12.0 Hz, 1H), 3.44 (d, *J* = 7.2 Hz, 1H); ^13^C-NMR δ (ppm) 194.4, 193.0, 159.2, 136.5, 135.2, 134.5, 134.4, 130.7, 130.6, 129.5, 129.4, 129.1, 129.0, 128.7, 128.4, 128.4, 126.8, 48.6, 46.5, 41.4, 39.9, 36.6; HRMS: *m*/*z* calculated for C_26_H_20_ClNO_3_Na^+^: 452.1029, found: 452.1028.

*1-Benzoyl-5-benzyl-2-(3-chlorophenyl)-5-azaspiro[2.4]heptane-6,7-dione* (**3i**). Purification of the crude product via flash chromatography on silica gel (petroleum ether/ethyl acetate = 4:1) to afford the corresponding **3i** as a white solid with 97:3 d.r., 94% yield; ^1^H-NMR δ (ppm) 8.05–7.97 (m, 2H), 7.70–7.62 (m, 1H), 7.53 (t, *J* = 7.8 Hz, 2H), 7.41–7.28 (m, 5H), 7.25 (m, 3H), 7.13 (m, 1H), 4.78 (d, *J* = 14.4 Hz, 1H), 4.69 (d, *J* = 14.4 Hz, 1H), 4.18 (d, *J* = 7.2 Hz, 1H), 3.86 (d, *J* = 12.0 Hz, 1H), 3.65 (d, *J* = 12.0 Hz, 1H), 3.54 (d, *J* = 7.2 Hz, 1H); ^13^C-NMR δ (ppm) 194.4, 193.4, 159.0, 136.4, 134.4, 134.4, 134.3, 133.7, 129.6, 129.1, 129.1, 129.1, 128.6, 128.4, 128.4, 128.3, 127.2, 48.8, 47.1, 43.0, 40.8, 36.5 HRMS: *m*/*z* calculated for C_26_H_20_ClNO_3_Na^+^: 452.1029, found: 452.1028.

*1-Benzoyl-5-benzyl-2-(4-chlorophenyl)-5-azaspiro[2.4]heptane-6,7-dione* (**3j**). Purification of the crude product via flash chromatography on silica gel (petroleum ether/ethyl acetate = 4:1) to afford the corresponding **3j** as a white solid with 96:4 d.r., 59% yield; ^1^H-NMR δ (ppm) 8.04–7.97 (m, 2H), 7.70–7.61 (m, 1H), 7.53 (t, *J* = 7.6 Hz, 2H), 7.35 (m, 2H), 7.32–7.24 (m, 5H), 7.22–7.15 (m, 2H), 4.79 (d, *J* = 14.4 Hz, 1H), 4.68 (d, *J* = 14.4 Hz, 1H), 4.17 (d, *J* = 7.2 Hz, 1H), 3.86 (d, *J* = 12.0 Hz, 1H), 3.66 (d, *J* = 12.0 Hz, 1H), 3.54 (d, *J* = 7.2 Hz, 1H); ^13^C-NMR δ (ppm) 194.6, 193.5, 159.1, 136.5, 134.5, 134.4, 134.1, 130.4, 130.3, 129.1, 129.1, 128.7, 128.6, 128.5, 128.4, 48.8, 47.2, 43.2, 41.0, 36.7; HRMS: *m*/*z* calculated for C_26_H_20_ClNO_3_Na^+^: 452.1029, found: 452.1028.

*1-Benzoyl-5-benzyl-2-(3-bromophenyl)-5-azaspiro[2.4]heptane-6,7-dione* (**3k**). Purification of the crude product via flash chromatography on silica gel (petroleum ether/ethyl acetate = 3:1) to afford the corresponding **3k** as a white solid with 94:6 d.r., 84% yield; ^1^H-NMR δ (ppm) 8.05–7.96 (m, 2H), 7.70–7.61 (m, 1H), 7.53 (t, *J* = 7.6 Hz, 2H), 7.45–7.38 (m, 2H), 7.35 (m, 3H), 7.30 (m, 2H), 7.21–7.15 (m, 2H), 4.78 (d, *J* = 14.4 Hz, 1H), 4.69 (d, *J* = 14.4 Hz, 1H), 4.18 (d, *J* = 7.2 Hz, 1H), 3.86 (d, *J* = 12.0 Hz, 1H), 3.65 (d, *J* = 12.0 Hz, 1H), 3.54 (d, *J* = 7.2 Hz, 1H); ^13^C-NMR δ (ppm) 194.4, 193.4, 159.0, 136.4, 134.4, 134.4, 134.0, 132.0, 131.3, 129.9, 129.1, 129.0, 128.6, 128.4, 128.4, 127.6, 122.4, 48.8, 47.1, 42.9, 40.8, 36.5; HRMS: *m*/*z* calculated for C_26_H_20_BrNO_3_Na^+^: 496.0524, found: 496.0526.

*1-Benzoyl-5-benzyl-2-(4-bromophenyl)-5-azaspiro[2.4]heptane-6,7-dione* (**3l**). Purification of the crude product via flash chromatography on silica gel (petroleum ether/ethyl acetate = 3:1) to afford the corresponding **3l** as a white solid with 96:4 d.r., 57% yield; ^1^H-NMR δ (ppm) 8.05–7.95 (m, 2H), 7.66 (t, *J* = 7.4 Hz, 1H), 7.53 (t, *J* = 7.6 Hz, 2H), 7.47–7.41 (m, 2H), 7.35 (m, 3H), 7.30 (m, 2H), 7.13 (d, *J* = 8.4 Hz, 2H), 4.79 (d, *J* = 14.4 Hz, 1H), 4.68 (d, *J* = 14.4 Hz, 1H), 4.17 (d, *J* = 7.2 Hz, 1H), 3.86 (d, *J* = 12.0 Hz, 1H), 3.66 (d, *J* = 12.0 Hz, 1H), 3.52 (d, *J* = 7.2 Hz, 1H); ^13^C-NMR δ (ppm) 194.5, 193.5, 159.0, 136.5, 134.4, 134.4, 131.6, 130.8, 130.6, 129.1, 129.1, 128.6, 128.4, 128.4, 122.2, 48.8, 47.3, 43.2, 40.9, 36.6; HRMS: *m*/*z* calculated for C_26_H_20_BrNO_3_Na^+^: 496.0524, found: 496.0522.

*1-Benzoyl-5-benzyl-2-(3,4-dimethoxyphenyl)-5-azaspiro[2.4]heptane-6,7-dione* (**3m**). Purification of the crude product via flash chromatography on silica gel (petroleum ether/ethyl acetate = 2:1) to afford the corresponding **3m** as a white solid with 98:2 d.r., 81% yield; ^1^H-NMR δ (ppm): 8.01 (m, 2H), 7.69–7.61 (m, 1H), 7.52 (t, *J* = 7.8 Hz, 2H), 7.40–7.28 (m, 5H), 6.87–6.76 (m, 3H), 4.79 (d, *J* = 14.4 Hz, 1H), 4.69 (d, *J* = 14.4 Hz, 1H), 4.19 (d, *J* = 7.6 Hz, 1H), 3.88 (s, 3H), 3.85 (s, 4H), 3.68 (d, *J* = 12.0Hz, 1H), 3.54 (d, *J* = 7.2 Hz, 1H); ^13^C-NMR δ (ppm): 194.8, 193.5, 159.3, 148.9, 148.7, 136.6, 134.5, 134.2, 129.0, 129.0, 128.5, 128.3, 128.3, 124.2, 121.5, 112.0, 110.9, 56.0, 55.9, 48.7, 47.3, 44.4, 41.4, 37.0; HR-MS (ESI): *m*/*z* calculated for C_28_H_25_NO_5_Na^+^: 478.1630, found: 478.1628.

*1-Benzoyl-5-benzyl-2-(2,4-dichlorophenyl)-5-azaspiro[2.4]heptane-6,7-dione* (**3n**). Purification of the crude product via flash chromatography on silica gel (petroleum ether/ethyl acetate = 4:1) to afford the corresponding **3n** as a white solid with >99:1 d.r., 99% yield; ^1^H-NMR δ (ppm) 8.07–7.98 (m, 2H), 7.72–7.62 (m, 1H), 7.54 (t, *J* = 7.6 Hz, 2H), 7.42–7.20 (m, 8H), 5.05 (d, *J* = 14.4 Hz, 1H), 4.42 (d, *J* = 14.4 Hz, 1H), 4.11 (d, *J* = 7.2 Hz, 1H), 3.91 (d, *J* = 12.0 Hz, 1H), 3.65 (d, *J* = 12.0 Hz, 1H), 3.38 (d, *J* = 7.2 Hz, 1H); ^13^C-NMR δ (ppm) 194.0, 193.0, 159.0, 136.3, 135.8, 134.7, 134.4, 131.4, 129.4, 129.2, 129.1, 128.9, 128.9, 128.7, 128.6, 128.4, 127.2, 48.6, 46.4, 40.4, 39.7, 36.4; HRMS: *m*/*z* calculated for C_26_H_19_Cl_2_NO_3_Na^+^: 486.0640, found: 486.0641.

*1-Benzoyl-5-benzyl-2-(naphthalen-1-yl)-5-azaspiro[2.4]heptane-6,7-dione* (**3o**). Purification of the crude product via flash chromatography on silica gel (petroleum ether/ethyl acetate = 3:1) to afford the corresponding **3o** as a white solid with 94:6 d.r., 94% yield; ^1^H-NMR δ (ppm) 8.15–8.08 (m, 2H), 7.84 (d, *J* = 8.2 Hz, 1H), 7.80 (d, *J* = 8.0 Hz, 1H), 7.71–7.65 (m, 1H), 7.61–7.51 (m, 3H), 7.51–7.44 (m, 5H), 7.40 (m, 4H), 5.20 (d, *J* = 14.4 Hz, 1H), 4.39 (d, *J* =6.8 Hz, 2H), 4.35 (d, *J* = 14.4 Hz, 2H), 4.14 (d, *J* = 12.4 Hz, 1H), 3.85 (s, 1H), 3.82 (d, *J* = 4.8Hz, 1H); ^13^C-NMR δ (ppm) 194.9, 192.6, 159.1, 136.6, 134.8, 134.3, 133.6, 132.5, 129.2, 129.1, 129.1, 129.0, 128.6, 128.5, 128.5, 128.0, 127.0, 126.9, 126.0, 125.0, 122.2, 48.6, 47.0, 42.0, 40.4, 36.1; HRMS: *m*/*z* calculated for C_28_H_25_NO_5_Na^+^: 468.1576, found: 468.1573.

*Ethyl-5-benzyl-6,7-dioxo-2-phenyl-5-azaspiro[2.4]heptane-1-carboxylate* (**3p**). Purification of the crude product via flash chromatography on silica gel (petroleum ether/ethyl acetate = 3:1) to afford the corresponding **3p** as a white solid with 98:2 d.r., 86% yield; ^1^H-NMR δ (ppm) 8.04–7.95 (m, 2H), 7.68–7.60 (m, 1H), 7.55–7.47 (m, 2H), 7.39–7.27 (m, 5H), 7.20 (dd, *J* = 5.2, 1.2 Hz, 1H), 7.04 (m, 1H), 6.95 (m, 1H), 4.76 (d, *J* = 14.4 Hz, 1H), 4.71 (d, *J* = 14.4 Hz, 1H), 4.18 (d, *J* = 7.0 Hz, 1H), 3.85 (d, *J* = 12.0 Hz, 1H), 3.66 (d, *J* = 12.0 Hz, 1H), 3.62 (dd, *J* = 7.0, 0.8Hz, 1H); ^13^C-NMR (100 MHz, CDCl_3_) δ (ppm) 194.2, 192.6, 159.1, 136.4, 134.4, 134.4, 134.2, 129.0, 128.9, 128.5, 128.3, 128.3, 127.5, 126.9, 125.6, 48.6, 47.0, 41.4, 38.5, 37.9; HRMS: *m*/*z* calculated for C_24_H_19_NO_3_SNa^+^: 424.0983, found: 424.0981.

*1-Benzoyl-5-(4-methoxybenzyl)-2-phenyl-5-azaspiro[2.4]heptane-6,7-dione* (**3q**). Purification of the crude product via flash chromatography on silica gel (petroleum ether/ethyl acetate = 4:1) to afford the corresponding **3q** as a white solid with 98:2 d.r., 90% yield; ^1^H-NMR δ (ppm) 8.04–7.98 (m, 2H), 7.69–7.60 (m, 1H), 7.52 (t, *J* = 8.0 Hz, 2H), 7.35–7.27 (m, 3H), 7.27–7.19 (m, 5H), 6.92–6.84 (m, 2H), 4.72 (d, *J* = 14.4 Hz, 1H), 4.64 (d, *J* = 14.4 Hz, 1H), 4.22 (d, *J* = 7.2 Hz, 1H), 3.86 (d, *J* = 12.0 Hz, 1H), 3.80 (s, 3H), 3.66 (d, *J* = 12.0 Hz, 1H), 3.58 (d, *J* = 7.2 Hz, 1H); ^13^C-NMR (100 MHz, CDCl_3_) δ (ppm) 194.9, 193.6, 159.6, 159.1, 136.6, 134.2, 131.7, 130.0, 129.0, 128.4, 128.4, 128.1, 126.5, 114.4, 55.3, 48.1, 47.1, 44.2, 41.1, 36.6; HRMS: *m*/*z* calculated for C_27_H_23_NO_4_Na^+^: 448.1525, found: 448.1528.

*Ethyl-5-benzyl-6,7-dioxo-2-phenyl-5-azaspiro[2.4]heptane-1-carboxylate* (**3r**). Purification of the crude product via flash chromatography on silica gel (petroleum ether/ethyl acetate = 4:1) to afford the corresponding **3r** as a white solid with 92:8 d.r., 99% yield; ^1^H-NMR δ (ppm) 7.42–7.34 (m, 3H), 7.34–7.30 (m, 2H), 7.29 (d, *J* = 1.8 Hz, 1H), 7.28–7.25 (m, 2H), 7.22–7.17 (m, 2H), 4.75 (s, 2H), 4.19 (qd, *J* = 7.2, 2.8 Hz, 2H), 3.77 (q, *J* = 12.0 Hz, 2H), 3.33 (d, *J* = 7.2 Hz, 1H), 3.21 (d, *J* = 7.2 Hz, 1H), 1.28 (t, *J* = 7.2 Hz, 3H); ^13^C-NMR δ (ppm) 192.9, 169.3, 159.1, 134.5, 131.1, 129.0, 128.9, 128.5, 128.3, 128.3, 128.0, 61.8, 48.6, 47.4, 42.9, 38.1, 33.5, 14.0; HRMS: *m*/*z* calculated for C_22_H_21_NO_4_Na^+^: 386.1386, found: 386.1385.

*Tert-butyl-5-benzyl-6,7-dioxo-2-phenyl-5-azaspiro[2.4]heptane-1-carboxylate* (**3s**). Purification of the crude product via flash chromatography on silica gel (petroleum ether/ethyl acetate = 4:1) to afford the corresponding **3s** as a white solid with 90:10 d.r., 99% yield; ^1^H-NMR δ (ppm) 7.30 (m, 3H), 7.28–7.24 (m, 2H), 7.22 (dd, *J* = 7.1, 1.8 Hz, 2H), 7.20–7.18 (m, 1H), 7.15–7.11 (m, 2H), 4.67 (s, 2H), 3.68 (d, *J* = 12.0 Hz, 1H), 3.62 (d, *J* = 12.0 Hz, 1H), 3.21 (d, *J* = 7.2 Hz, 1H), 3.05 (d, *J* = 7.2 Hz, 1H), 1.36 (s, 9H); ^13^C-NMR δ (ppm) 192.1, 167.3, 158.2, 133.5, 130.3, 128.0, 127.9, 127.5, 127.3, 127.2, 126.9, 81.8, 47.5, 46.3, 41.6, 37.0, 33.9, 27.0; HRMS: *m*/*z* calculated for C_24_H_25_NO_4_Na^+^: 414.1681, found: 414.1680.

#### 3.2.2. General Procedure for the Synthesis of Multi-Substituted Spirocyclopropane **5**

A dried glass tube was charged with cyclic enones **1** (0.1 mmol) and amidic sulfonium salt **4** (0.1 mmol) at the presence of TMG (13 μL, 0.1 mmol, 1.0 equiv.) in 1,4-dioxane (0.5 M, 2 mL). The reaction was sealed with a Teflon cap and stirred at room temperature overnight. When the reaction was complete, the reaction mixture was concentrated and the residue was purified by flash chromatography on silica gel (petroleum ether/ethyl acetate) to afford the corresponding spirocyclopropane **5**, which was dried under vacuum oven and further analyzed by ^1^H-NMR, ^13^C-HMR, HRMS, etc.

*N,5-Dibenzyl-6,7-dioxo-2-phenyl-5-azaspiro[2.4]heptane-1-carboxamide* (**5a**). Purification of the crude product via flash chromatography on silicagel (petroleum ether/ethyl acetate = 2:1) to afford the corresponding **5a** as a white solid with 96:4 d.r., 98% yield; ^1^H-NMR δ (ppm) 8.27 (s, 1H), 7.36–7.30 (m, 3H), 7.26 (m, 5H), 7.21 (m, 2H), 7.14 (m, 4H), 4.73 (d, *J* = 4.4 Hz, 2H), 4.48 (dd, *J* = 15.2, 6.4 Hz, 1H), 4.36 (dd, *J* = 15.2, 5.6 Hz, 1H), 3.68 (d, *J* = 5.2 Hz, 2H), 3.53 (d, *J* = 7.6 Hz, 1H), 3.15 (d, *J* = 7.2 Hz, 1H); ^13^C-NMR δ (ppm) 195.1, 167.0, 160.3, 138.1, 134.2, 132.3, 129.2, 129.1, 128.9, 128.4, 128.2, 128.2, 127.6, 127.3, 126.9, 48.7, 47.7, 43.6, 41.4, 38.1, 36.9; HRMS: *m*/*z* calculated for C_27_H_24_N2O_3_Na^+^: 447.1685, found: 447.1684.

*5-Benzyl-N-(4-methoxybenzyl)-6,7-dioxo-2-phenyl-5-azaspiro[2.4]heptane-1-carboxamide* (**5b**). Purification of the crude product via flash chromatography on silica gel (petroleum ether/ethyl acetate = 2:1) to afford the corresponding **5b** as a white solid with >99:1 d.r., 99% yield; ^1^H-NMR δ (ppm) 7.91 (t, *J* = 5.6 Hz, 1H), 7.27–7.24 (m, 3H), 7.24–7.16 (m, 5H), 7.10–7.03 (m, 4H), 6.69–6.60 (m, 2H), 4.67 (s, 2H), 4.32 (dd, *J* = 15.0, 6.2 Hz, 1H), 4.24 (dd, *J* = 14.8, 5.4 Hz, 1H), 3.70 (s, 3H), 3.62 (d, *J* = 8.8 Hz, 2H), 3.45 (d, *J* = 7.2 Hz, 1H), 3.03 (d, *J* = 7.6 Hz, 1H); ^13^C-NMR δ (ppm) 195.0, 166.9, 160.3, 158.6, 134.2, 132.3, 130.2, 129.2, 129.1, 128.7, 128.2, 128.2, 128.2, 127.6, 113.8, 55.2, 48.7, 47.7, 43.2, 41.4, 38.1, 37.0; HRMS: *m*/*z* calculated for C_28_H_26_N_2_O_4_Na^+^: 477.1790, found: 477.1785.

*N,5-Dibenzyl-6,7-dioxo-2-(p-tolyl)-5-azaspiro[2.4]heptane-1-carboxamide* (**5c**). Purification of the crude product via flash chromatography on silica gel (petroleum ether/ethyl acetate = 2:1) to afford the corresponding **5c** as a white solid with 97:3 d.r., 99% yield; ^1^H-NMR δ (ppm) 7.92 (s, 1H), 7.27–7.23 (m, 3H), 7.21–7.20 (m, 3H), 7.16–7.13 (m, 2H) , 7.12–7.09 (m, 2H), 7.02–6.92 (m, 4H), 4.67 (s, 2H), 4.41 (dd, *J* = 15.2, 6.0 Hz, 1H), 4.31 (dd, *J* = 15.2, 5.6 Hz, 1H), 3.62 (q, *J* = 16.8, 12.4 Hz, 2H), 3.43 (d, *J* = 7.4 Hz, 1H), 3.02 (d, *J* = 7.6 Hz, 1H), 2.26 (s, 3H); ^13^C-NMR δ (ppm) 194.9, 167.1, 160.3, 138.0, 137.3, 134.3, 129.2, 129.1, 129.0, 128.9, 128.4, 128.3, 128.2, 127.4, 127.0, 48.7, 47.8, 43.7, 41.5, 38.3, 36.9, 21.1; HRMS: *m*/*z* calculated for C_28_H_26_N_2_O_3_Na^+^: 461.1841, found: 461.1838.

*5-Benzyl-N-(4-methoxybenzyl)-6,7-dioxo-2-(p-tolyl)-5-azaspiro[2.4]heptane-1-carboxamide* (**5d**). Purification of the crude product via flash chromatography on silica gel (petroleum ether/ethyl acetate = 2:1) to afford the corresponding **5d** as a white solid with 94:6 d.r., 92% yield; ^1^H-NMR δ (ppm) 7.97 (t, *J* = 5.8 Hz, 1H), 7.27–7.16 (m, 5H), 7.10–7.03 (m, 2H), 7.01–6.92 (m, 4H), 6.67–6.60 (m, 2H), 4.66 (s, 2H), 4.27 (qd, *J* = 15.0, 5.8 Hz, 2H), 3.69 (s, 3H), 3.67–3.55 (m, 2H), 3.41 (d, *J* = 7.2 Hz, 1H), 3.01 (d, *J* = 7.6 Hz, 1H); ^13^C-NMR δ (ppm) 195.0, 167.0, 160.3, 158.6, 137.3, 134.3, 130.2, 129.2, 129.1, 129.0, 128.9, 128.7, 128.2, 128.2, 113.8, 55.2, 48.6, 47.7, 43.1, 41.3, 38.2, 37.1, 21.1; HRMS: *m*/*z* calculated for C_29_H_28_N_2_O_4_Na^+^: 491.1947, found: 491.1958.

*N,5-Dibenzyl-2-(4-chlorophenyl)-6,7-dioxo-5-azaspiro[2.4]heptane-1-carboxamide* (**5e**). Purification of the crude product via flash chromatography on silica gel (petroleum ether/ethyl acetate = 2:1) to afford the corresponding **5e** as a white solid with 91:9 d.r., 99% yield; ^1^H-NMR δ (ppm) 8.24 (t, *J* = 6.0 Hz, 1H), 7.40–7.29 (m, 3H), 7.29–7.24 (m, 3H), 7.24–7.13 (m, 6H), 7.11–7.01 (m, 2H), 4.80 (d, *J* = 14.4 Hz, 1H), 4.68 (d, *J* = 14.4 Hz, 1H), 4.52 (dd, *J* = 15.2, 6.4 Hz, 1H), 4.35 (dd, *J* = 15.2, 5.6 Hz, 1H), 3.68 (q, *J* = 15.6, 12.4 Hz, 2H), 3.50 (d, *J* = 7.4 Hz, 1H), 3.10 (d, *J* = 7.6 Hz, 1H); ^13^C-NMR δ (ppm) 195.2, 166.7, 160.2, 137.9, 134.0, 133.5, 130.8, 130.5, 129.2, 128.4, 128.4, 128.4, 128.1, 127.3, 127.1, 48.7, 47.6, 43.7, 40.4, 37.9, 37.1; HRMS: *m*/*z* calculated for C_27_H_23_ClN_2_O_3_Na^+^: 481.1295, found: 481.1296.

*5-Benzyl-2-(2-chlorophenyl)-N-(4-methoxybenzyl)-6,7-dioxo-5-azaspiro[2.4]heptane-1-carboxamide* (**5f**). Purification of the crude product via flash chromatography on silica gel (petroleum ether/ethyl acetate = 2:1) to afford the corresponding **5f** as a white solid with 94:6 d.r., 98% yield; ^1^H-NMR δ (ppm) 8.25–8.08 (m, 1H), 7.27–7.20 (m, 4H), 7.20–7.11 (m, 5H), 7.10–7.03 (m, 2H), 6.71–6.63 (m, 2H), 4.88 (d, *J* = 14.4 Hz, 1H), 4.40 (d, *J* = 14.4 Hz, 1H), 4.28 (qd, *J* = 14.8, 5.6 Hz, 2H), 3.72 (s, 3H), 3.68 (d, *J* = 12.2 Hz, 1H), 3.54 (d, *J* = 12.2 Hz, 1H), 3.22 (d, *J* = 7.4 Hz, 1H), 3.00–2.87 (m, 1H); ^13^C-NMR δ (ppm) 194.6, 166.6, 160.2, 158.7, 135.2, 134.3, 131.4, 130.9, 130.2, 129.2, 129.0, 129.0, 129.0, 128.8, 128.6, 128.2, 126.7, 113.8, 55.2, 48.5, 47.0, 43.3, 38.9, 37.0; HRMS: *m*/*z* calculated for C_28_H_25_ClN_2_O_4_Na^+^: 511.1401, found: 511.1401.

*N,5-Dibenzyl-2-(naphthalen-2-yl)-6,7-dioxo-5-azaspiro[2.4]heptane-1-carboxamide* (**5g**). Purification of the crude product via flash chromatography on silica gel (petroleum ether/ethyl acetate = 2:1) to afford the corresponding **5g** as a white solid with >99:1 d.r., 85% yield; ^1^H-NMR δ (ppm) 7.92 (t, *J* = 6.0 Hz, 1H), 7.82–7.80 (m, 1H), 7.76–7.68 (m, 1H), 7.63 (d, *J* = 1.6 Hz, 1H), 7.54–7.40 (m, 2H), 7.31–7.15 (m, 9H), 7.14–7.06 (m, 1H), 7.06–6.98 (m, 2H), 4.76 (s, 2H), 4.49 (dd, *J* = 15.2, 6.4 Hz, 1H), 4.35 (dd, *J* = 15.2, 5.2 Hz, 1H), 3.76 (d, *J* = 12.4 Hz, 1H), 3.72–3.62 (m, 2H), 3.22 (d, *J* = 7.6 Hz, 1H); ^13^C-NMR δ (ppm) 194.9, 167.0, 160.2, 137.9, 134.2, 133.0, 132.8, 129.8, 129.1, 128.4, 128.3, 128.3, 128.2, 128.0, 127.9, 127.6, 127.3, 127.1, 127.0, 126.3, 126.2, 48.7, 47.7, 43.7, 41.5, 38.1, 37.0; HRMS: *m*/*z* calculated for C_31_H_26_N_2_O_3_Na^+^: 497.1841, found: 497.1841.

*5-Benzyl-N-(4-methoxybenzyl)-2-(naphthalen-2-yl)-6,7-dioxo-5-azaspiro[2.4]heptane-1-carboxamide* (**5h**). Purification of the crude product via flash chromatography on silica gel (petroleum ether/ethyl acetate = 2:1) to afford the corresponding **5h** as a white solid with >99:1 d.r., 90% yield; ^1^H-NMR δ (ppm) 7.76–7.61 (m, 3H), 7.58 (s, 1H), 7.43–7.33 (m, 2H), 7.20 (d, *J* = 9.1 Hz, 7H), 7.14–7.12 (m, 1H), 7.09–7.02 (m, 2H), 6.63 (d, *J* = 8.4 Hz, 2H), 4.68 (dq, *J* = 12.6, 14.4 Hz, 2H), 4.29 (dd, *J* = 5.7, 3.3 Hz, 2H), 4.33–4.26 (m, 2H), 3.77–3.70 (m, 2H), 3.66 (s, 3H), 3.63–3.56 (m, 1H), 3.08 (d, *J* = 7.4 Hz, 1H); ^13^C-NMR δ (ppm) 195.0, 166.8, 160.2, 158.7, 134.2, 133.0, 132.7, 130.0, 129.8, 129.1, 128.8, 128.3, 128.2, 128.2, 127.9, 127.8, 127.6, 127.0, 126.3, 126.1, 113.8, 55.2, 48.7, 47.7, 43.3, 41.5, 38.1, 37.2; HRMS: *m*/*z* calculated for C_32_H_28_N_2_O_4_Na^+^: 527.1947, found: 527.1943.

*N,5-Dibenzyl-6,7-dioxo-2-(thiophen-2-yl)-5-azaspiro[2.4]heptane-1-carboxamide* (**5i**). Purification of the crude product via flash chromatography on silica gel (petroleum ether/ethyl acetate = 2:1) to afford the corresponding **5i** as a white solid with >99:1 d.r., 83% yield; ^1^H-NMR δ (ppm) 8.44–8.21 (m, 1H), 7.32–7.25 (m, 3H), 7.23–7.20 (m, 2H), 7.18–7.13 (m, 2H), 7.13–7.09 (m, 4H), 6.86–6.76 (m, 2H), 4.74 (d, *J* = 14.6 Hz, 1H), 4.61 (d, *J* = 14.6 Hz, 1H), 4.43 (dd, *J* = 15.2, 6.4 Hz, 1H), 4.28 (dd, *J* = 15.2, 5.4 Hz, 1H), 3.61 (s, 2H), 3.51 (d, *J* = 6.8 Hz, 1H), 3.11–2.97 (m, 1H); ^13^C-NMR δ (ppm) 194.3, 166.5, 160.3, 138.0, 135.2, 134.2, 129.1, 128.4, 128.3, 127.4, 127.3, 127.0, 126.7, 125.3, 48.7, 47.5, 43.7, 38.6, 38.3, 35.9; HRMS: *m*/*z* calculated for C_25_H_22_N_2_O_3_SNa^+^: 453.1249, found: 453.1250.

*5-Benzyl-N-(4-methoxybenzyl)-6,7-dioxo-2-(thiophen-2-yl)-5-azaspiro[2.4]heptane-1-carboxamide* (**5j**). Purification of the crude product via flash chromatography on silica gel (petroleum ether/ethyl acetate = 2:1) to afford the corresponding **5j** as a white solid with >99:1 d.r., 81% yield; ^1^H-NMR δ (ppm) 8.20–8.01 (m, 1H), 7.39–7.32 (m, 3H), 7.32–7.28 (m, 2H), 7.21–7.11 (m, 3H), 6.92–6.89 (m, 1H), 6.89–6.82 (m, 1H), 6.77–6.69 (m, 2H), 4.80 (d, *J* = 14.6 Hz, 1H), 4.70 (d, *J* = 14.6 Hz, 1H), 4.42 (dd, *J* = 14.9, 6.2 Hz, 1H), 4.30 (dd, *J* = 14.8, 6.0 Hz, 1H), 3.77 (s, 3H), 3.69 (d, *J* = 1.8 Hz, 2H), 3.58 (d, *J* = 7.2, 1H), 3.07 (d, *J* = 7.2 Hz, 1H); ^13^C-NMR δ (ppm) 194.2, 166.3, 160.2, 158.7, 135.2, 134.2, 130.1, 129.1, 128.7, 128.3, 127.4, 126.7, 125.3, 113.9, 55.2, 48.7, 47.5, 43.3, 38.6, 38.3, 35.9; HRMS: *m*/*z* calculated for C_26_H_24_N_2_O_4_Na^+^: 483.1354, found: 483.1356.

#### 3.2.3. General Procedure for the Synthesis of Multi-substituted Chiral Spirocyclopropane **6**

A dried glass tube was charged with cyclic enone **1** (0.1 mmol) and (*S*)-dimethyl(2-oxo-2-((1-phenylethyl)amino)ethyl)sulfonium bromide **4c** (0.1 mmol) at the presence of TMG (13 μL, 0.1 mmol, 1.0 equiv.) in 1,4-dioxane (0.5 M, 2 mL). The reaction was sealed with a Teflon cap and stirred at room temperature overnight. When the reaction was complete, the reaction mixture was concentrated and the residue was purified by flash chromatography on silica gel (methanol/dichloromethane) to afford the corresponding chiral spirocyclopropane **6**, which was dried under vacuum oven and further analyzed by ^1^H-NMR, ^13^C-HMR, HRMS, etc.

*(1S,2R,3S)-5-Benzyl-6,7-dioxo-2-phenyl-N-((S)-1-phenylethyl)-5-azaspiro[2.4]heptane-1-carboxamide* (**6a**). Purification of the crude product via flash chromatography on silica gel (methanol/dichloromethane = 1:300) to afford the corresponding 6a as a white solid with 72:28 d.r., 99% yield, [α]${}_{\text{D}}^{20}$ = +94.6 (*c* = 0.84 in CHCl_3_); ^1^H-NMR δ (ppm) 7.31–7.23 (m, 3H), 7.23–7.14 (m, 10H), 7.14–7.08 (m, 2H), 7.01 (d, *J* = 7.6 Hz, 1H), 5.03–4.86 (m, 1H), 4.73 (d, *J* = 14.4 Hz, 1H), 4.34 (d, *J* = 14.4 Hz, 1H), 3.57 (d, *J* = 12.2 Hz, 1H), 3.46–3.32 (m, 2H), 2.99 (d, *J* = 7.2 Hz, 1H), 1.38 (d, *J* = 7.0 Hz, 3H); ^13^C-NMR δ (ppm) 194.9, 166.1, 159.5, 143.3, 134.5, 132.1, 129.2, 129.0, 128.6, 128.5, 128.2, 128.2, 127.7, 127.2, 126.2, 49.8, 48.6, 47.5, 41.4, 38.1, 36.6, 21.8; HRMS: *m*/*z* calculated for C_28_H_26_N_2_O_3_Na^+^: 461.1841, found: 461.1832.

*(1S,2R,3S)-5-Benzyl-6,7-dioxo-N-((S)-1-phenylethyl)-2-(p-tolyl)-5-azaspiro[2.4]heptane-1-carboxamide* (**6b**). Purification of the crude product via flash chromatography on silica gel (methanol/dichloromethane = 1:300) to afford the corresponding **6b** as a white solid with 70:30 d.r., 97% yield, [α]${}_{\text{D}}^{20}$ = +96.6 (*c* = 0.54 in CHCl_3_); ^1^H-NMR δ (ppm) 7.35–7.30 (m, 3H), 7.28–7.19 (m, 7H), 7.07 (s, 4H), 7.04–7.01 (m, 1H), 5.07–4.97 (m, 1H), 4.79 (d, *J* = 14.4 Hz, 1H), 4.47 (d, *J* = 14.4 Hz, 1H), 3.64 (d, *J* = 12.2 Hz, 1H), 3.50–3.41 (m, 2H), 3.03 (d, *J* = 7.2 Hz, 1H), 2.31 (s, 3H), 1.46 (d, *J* = 7.0 Hz, 3H); ^13^C-NMR δ (ppm) 194.8, 166.2, 159.6, 143.3, 137.4, 134.6, 129.1, 129.0, 129.0, 128.9, 128.6, 128.5, 128.2, 127.2, 126.1, 49.7, 48.6, 47.5, 41.5, 38.3, 36.6, 21.9, 21.1; HRMS: *m*/*z* calculated for C_29_H_28_N_2_O_3_Na^+^: 475.1998, found: 475.1995.

*(1S,2R,3S)-5-Benzyl-2-(4-methoxyphenyl)-6,7-dioxo-N-((S)-1-phenylethyl)-5-azaspiro[2.4]heptane-1-carboxamide* (**6c**). Purification of the crude product via flash chromatography on silica gel (methanol/dichloromethane = 1:300) to afford the corresponding **6c** as a white solid with 64:36 d.r., 91% yield, [α]${}_{\text{D}}^{20}$ = +94.6 (*c* = 0.84 in CHCl_3_); ^1^H-NMR δ (ppm) 7.35–7.27 (m, 6H), 7.25–7.18 (m, 5H), 7.11 (d, *J* = 8.0 Hz, 2H), 6.82–6.75 (m, 2H), 5.06–4.97 (m, 1H), 4.79 (d, *J* = 14.4 Hz, 1H), 4.39 (d, *J* = 14.4 Hz, 1H), 3.76 (s, 3H), 3.62 (d, *J* = 12.2 Hz, 1H), 3.49–3.39 (m, 2H), 3.09 (d, *J* = 7.2 Hz, 1H), 1.44 (d, *J* = 7.0 Hz, 3H); ^13^C-NMR δ (ppm) 194.9, 166.2, 159.6, 159.0, 143.4, 134.6, 130.3, 128.9, 128.5, 128.4, 128.2, 127.1, 126.2, 124.0, 113.5, 55.2, 49.7, 48.6, 47.5, 41.2, 38.4, 36.8, 21.8; HRMS: *m*/*z* calculated for C_29_H_28_N_2_O_4_Na+: 491.1947, found: 491.1947.

*(1S,2R,3S)-5-Benzyl-2-(4-fluorophenyl)-6,7-dioxo-N-((S)-1-phenylethyl)-5-azaspiro[2.4]heptane-1-carboxamide* (**6d**). Purification of the crude product via flash chromatography on silica gel (methanol/dichloromethane = 1:300) to afford the corresponding **6d** as a white solid with 81:19 d.r., 92% yield, [α]${}_{\text{D}}^{20}$ = +112.0 (*c* = 0.97 in CHCl_3_); ^1^H-NMR δ (ppm) 7.36–7.27 (m, 6H), 7.26–7.20 (m, 5H), 7.17–7.12 (m, 2H), 6.96–6.89 (m, 2H), 5.07–4.98 (m, 1H), 4.79 (d, *J* = 14.4 Hz, 1H), 4.41 (d, *J* = 14.4 Hz, 1H), 3.63 (d, *J* = 12.2 Hz, 1H), 3.47–3.39 (m, 2H), 3.06 (d, *J* = 7.2 Hz, 1H), 1.47 (d, *J* = 7.0 Hz, 3H); ^13^C-NMR δ (ppm) 195.0, 165.9, 159.5, 143.3, 134.4, 130.9, 130.8, 129.0, 128.5, 128.5, 128.3, 127.2, 126.2, 115.2, 115.0, 49.8, 48.7, 47.4, 40.5, 38.0, 36.8, 21.8; HRMS: *m*/*z* calculated for C_28_H_25_FN_2_O_3_Na^+^: 479.1747, found: 479.1749.

*(1S,2S,3S)-5-Benzyl-2-(2-chlorophenyl)-6,7-dioxo-N-((S)-1-phenylethyl)-5-azaspiro[2.4]heptane-1-carboxamide* (**6e**). Purification of the crude product via flash chromatography on silica gel (methanol/dichloromethane = 1:300) to afford the corresponding **6e** as a white solid with 70:30 d.r., 90% yield, [α]${}_{\text{D}}^{20}$ = +104.3 (*c* = 1.01 in CHCl_3_); ^1^H-NMR δ (ppm) 7.38–7.28 (m, 7H), 7.27–7.20 (m, 7H), 7.19–7.13 (m, 1H), 5.09–4.93 (m, 2H), 4.22 (d, *J* = 14.4 Hz, 1H), 3.71 (d, *J* = 12.2 Hz, 1H), 3.44 (d, *J* = 12.2 Hz, 1H), 3.28 (d, *J* = 7.2 Hz, 1H), 2.96 (d, *J* = 7.2 Hz, 1H), 1.48 (d, *J* = 7.0 Hz, 3H); ^13^C-NMR δ (ppm) 194.5, 165.7, 159.4, 143.4, 135.3, 134.5, 131.2, 130.9, 129.1, 129.1, 128.9, 128.6, 128.5, 128.2, 127.2, 126.5, 126.2, 49.8, 48.5, 48.5, 46.7, 39.0, 37.0, 36.5, 21.8; HRMS: *m*/*z* calculated for C_28_H_25_ClN_2_O_3_Na^+^: 495.1451, found: 495.1449.

*(1S,2R,3S)-5-Benzyl-2-(4-bromophenyl)-6,7-dioxo-N-((S)-1-phenylethyl)-5-azaspiro[2.4]heptane-1-carboxamide* (**6f**). Purification of the crude product via flash chromatography on silica gel (methanol/dichloromethane = 1:300) to afford the corresponding **6f** as a white solid with 62:38 d.r., 81% yield, [α]${}_{\text{D}}^{20}$ = +106.8 (*c* = 0.44 in CHCl_3_); ^1^H-NMR δ (ppm) 7.40–7.32 (m, 5H), 7.31–7.27 (m, 2H), 7.27–7.21 (m, 5H), 7.06–6.98 (m, 3H), 5.09–4.99 (m, 1H), 4.79 (d, *J* = 14.4 Hz, 1H), 4.42 (d, *J* = 14.4 Hz, 1H), 3.64 (d, *J* = 12.2 Hz, 1H), 3.46 (d, *J* = 12.2 Hz, 1H), 3.40 (d, *J* = 7.2 Hz, 1H), 2.99 (d, *J* = 7.2 Hz, 1H), 1.49 (d, *J* = 7.0 Hz, 3H); ^13^C-NMR δ (ppm) 194.8, 165.7, 159.3, 143.1, 134.4, 131.3, 131.1, 130.9, 129.0, 128.6, 128.5, 128.3, 127.3, 126.2, 121.7, 49.8, 48.7, 47.4, 40.5, 37.9, 36.6, 21.7; HRMS: *m*/*z* calculated for C_28_H_25_BrN_2_O_3_Na^+^: 539.0946, found: 539.0935.

*(1S,2R,3S)-5-Benzyl-2-(naphthalen-1-yl)-6,7-dioxo-N-((S)-1-phenylethyl)-5-azaspiro[2.4]heptane-1-carboxamide* (**6g**). Purification of the crude product via flash chromatography on silica gel (methanol/dichloromethane = 1:300) to afford the corresponding **6g** as a white solid with 77:28 d.r., 97% yield, [α]${}_{\text{D}}^{20}$ = +116.9 (*c* = 0.12 in CHCl_3_); ^1^H-NMR δ (ppm) 7.78–7.70 (m, 2H), 7.41–7.31 (m, 6H), 7.29–7.23 (m, 4H), 7.22–7.15 (m, 5H), 6.85 (d, *J* = 7.8 Hz, 1H), 5.07 (d, *J* = 14.4 Hz, 1H), 4.99–4.90 (m, 1H), 4.09 (d, *J* = 14.4 Hz, 1H), 3.82 (d, *J* = 12.2 Hz, 1H), 3.63 (d, *J* = 7.2 Hz, 1H), 3.51 (d, *J* = 12.2 Hz, 1H), 3.08 (d, *J* = 7.2 Hz, 1H), 1.32 (d, *J* = 7.0 Hz, 3H); ^13^C-NMR δ (ppm) 194.0, 166.1, 159.3, 143.3, 134.8, 133.5, 132.6, 129.0, 129.0, 128.7, 128.6, 128.5, 128.4, 128.4, 127.3, 127.1, 126.8, 126.2, 125.9, 124.9, 122.4, 49.9, 48.5, 47.1, 39.4, 37.6, 36.0, 21.8; HRMS: *m*/*z* calculated for C_32_H_28_N_2_O_3_Na^+^: 511.1998, found: 511.2004.

*(1S,2R,3S)-5-Benzyl-2-(naphthalen-2-yl)-6,7-dioxo-N-((S)-1-phenylethyl)-5-azaspiro[2.4]heptane-1-carboxamide* (**6h**). Purification of the crude product via flash chromatography on silica gel (methanol/dichloromethane = 1:300) to afford the corresponding **6h** as a white solid with 77:23 d.r., 99% yield, [α]${}_{\text{D}}^{20}$ = +120.0 (*c* = 0.40 in CHCl_3_); ^1^H-NMR δ (ppm) 7.83–7.76 (m, 2H), 7.73–7.66 (m, 2H), 7.51–7.45 (m, 2H), 7.34 (q, *J* = 3.8 Hz, 3H), 7.30–7.22 (m, 8H), 6.53 (d, *J* = 7.6 Hz, 1H), 5.11–5.00 (m, 1H), 4.79 (d, *J* = 14.4 Hz, 1H), 4.53 (d, *J* = 14.4 Hz, 1H), 3.74 (d, *J* = 12.2 Hz, 1H), 3.61 (d, *J* = 7.4 Hz, 1H), 3.56 (d, *J* = 12.2 Hz, 1H), 3.07 (d, *J* = 7.4 Hz, 1H), 1.47 (d, *J* = 7.0 Hz, 3H); ^13^C-NMR δ (ppm) 194.5, 166.1, 159.4, 144.1, 135.5, 132.5, 132.2, 130.8, 128.8, 128.3, 128.0, 127.8, 127.7, 127.5, 127.5, 127.4, 126.7, 126.3, 126.0, 125.8, 48.4, 47.5, 47.4, 40.6, 38.1, 34.7, 22.5; HRMS: *m*/*z* calculated for C_32_H_28_N_2_O_3_Na^+^: 511.1998, found: 511.1997.

*(1S,2S,3R)-5-Benzyl-6,7-dioxo-N-((S)-1-phenylethyl)-2-(thiophen-2-yl)-5-azaspiro[2.4]heptane-1-carboxamide* (**6i**). Purification of the crude product via flash chromatography on silica gel (methanol/dichloromethane = 1:300) to afford the corresponding **6i** as a white solid with 60:40 d.r., 90% yield, [α]${}_{\text{D}}^{20}$ = +97.6 (*c* = 0.54 in CHCl_3_); ^1^H-NMR δ (ppm) 7.38–7.32 (m, 3H), 7.31–7.22 (m, 7H), 7.20–7.17 (m, 1H), 7.00–6.96 (m, 1H), 6.95–6.90 (m, 1H), 6.83 (d, *J* = 7.6 Hz, 1H), 5.11–4.97 (m, 1H), 4.76 (d, *J* = 14.4 Hz, 1H), 4.52 (d, *J* = 14.4 Hz, 1H), 3.65 (d, *J* = 12.2 Hz, 1H), 3.56–3.43 (m, 2H), 3.04 (d, *J* = 7.0 Hz, 1H), 1.49 (d, *J* = 7.0 Hz, 3H); ^13^C-NMR δ (ppm) 193.8, 165.5, 159.4, 143.0, 134.8, 134.5, 129.0, 128.7, 128.5, 128.3, 127.6, 127.4, 126.8, 126.1, 125.5, 49.9, 48.7, 47.2, 38.7, 37.7, 36.1, 21.8; HR-MS (ESI): *m*/*z* calculated for C_26_H_24_N_2_O_3_SNa^+^: 467.1405, found: 467.1406.

## 4. Conclusions

In conclusion, we have developed a highly diastereoselective cyclopropanation reaction of readily available cyclic enones with sulfur ylides. An array of ketone or amide substituted spirocyclopanane derivatives with high molecular complexity were efficiently produced in a concise procedure. A series of chiral spirocyclopananes were also successfully accessed by using a chiral amidic sulfur ylide in excellent yields with moderate to good stereoselectivity. Currently the development of a catalytic asymmetric version of this cyclopropanation is under investigation in our laboratory.

## Figures and Tables

**Table 1:**
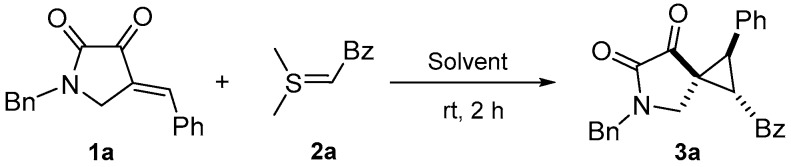
Screening studies of the cyclopropanation reaction ^a^.

Entry	Solvent	d.r. ^b^	Yield (%) ^c^
1	DCM	93:7	86
2	CHCl_3_	96:4	94
3	DCE	94:6	90
4	THF	97:3	79
5	1,4-dioxane	98:2	92
6	MeOH	53:47	76
7	EtOH	68:32	74
8	MeCN	84:16	92
9	Toluene	87:13	88

^a^ Reaction conditions: **1a** (0.1 mmol), **2a** (0.1 mmol), solvent (2 mL), r.t. DCE = 1,2-dichloroethane; Bn = benzyl; Bz = benzoyl; ^b^ Determined by ^1^H-NMR spectroscopy of the crude reaction mixture; ^c^ Isolated yields.

**Table 2:**
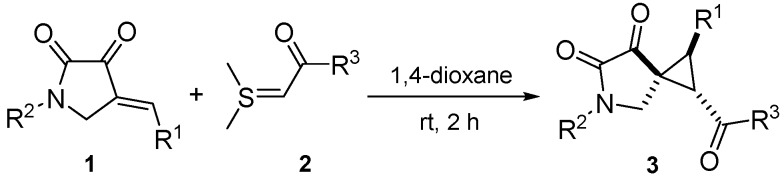
Substrates scope of cyclopropanation of cyclic enones **1** with sulfur ylides **2** ^a^.

Entry	R^1^	R^2^	R^3^	Product	d.r. ^b^	Yield (%) ^c^
1	Ph	Bn	Ph	**3a**	98:2	92
2	3-MeC_6_H_4_	Bn	Ph	**3b**	92:8	83
3	4-MeC_6_H_4_	Bn	Ph	**3c**	98:2	93
4	2-MeOC_6_H_4_	Bn	Ph	**3d**	92:8	83
5	3-MeOC_6_H_4_	Bn	Ph	**3e**	97:3	91
6	4-MeOC_6_H_4_	Bn	Ph	**3f**	96:4	94
7	4-FC_6_H_4_	Bn	Ph	**3g**	96:4	88
8	2-ClC_6_H_4_	Bn	Ph	**3h**	97:3	98
9	3-ClC_6_H_4_	Bn	Ph	**3i**	97:3	94
10	4-ClC_6_H_4_	Bn	Ph	**3j**	96:4	59
11	3-BrC_6_H_4_	Bn	Ph	**3k**	94:6	84
12	4-BrC_6_H_4_	Bn	Ph	**3l**	96:4	57
13	3,4-(MeO)_2_C_6_H_3_	Bn	Ph	**3m**	98:2	81
14 ^d^	2,4-Cl_2_C_6_H_3_	Bn	Ph	**3n**	>99:1	99
15	1-Naphthyl	Bn	Ph	**3o**	94:6	94
16	2-Thienyl	Bn	Ph	**3p**	98:2	86
17	Ph	PMB	Ph	**3q**	98:2	90
18 ^e^	Ph	Bn	OEt	**3r**	92:8	99
19 ^e^	Ph	Bn	O*t*-Bu	**3s**	90:10	99

^a^ Unless otherwise noted, reaction was carried out with **1** (0.1 mmol), **2** (0.1 mmol) in 2 mL of 1,4-dioxane at r.t. PMB = *p*-methoxybenzyl; ^b^ Determined by ^1^H-NMR spectroscopy of the crude reaction mixture; ^c^ Isolated yields; ^d^ The absolute configuration of **3n** was determined by X-ray analysis. Other products were assigned by analogy; ^e^ Sulfonium bromide salts and 0.1 mmol extra TMG was used instead of **2**.

**Table 3:**
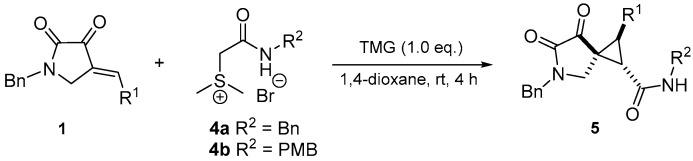
Further studies of the cyclopropanation of cyclic enone **1** and sulfonium salts **4** ^a^.

Entry	R^1^	R^2^	Product	d.r. ^b^	Yield (%) ^c^
1	Ph	Bn	**5a**	96:4	98
2	Ph	PMB	**5b**	>99:1	99
3	3-MeC_6_H_4_	Bn	**5c**	97:3	99
4	3-MeC_6_H_4_	PMB	**5d**	94:6	92
5	4-ClC_6_H_4_	Bn	**5e**	91:9	99
6	2-ClC_6_H_4_	PMB	**5f**	94:6	98
7 ^d^	2-Naphthyl	Bn	**5g**	> 99:1	85
8 ^d^	2-Naphthyl	PMB	**5h**	> 99:1	90
9 ^d^	2-Thienyl	Bn	**5i**	> 99:1	83
10 ^d^	2-Thienyl	PMB	**5j**	> 99:1	81

^a^ Unless otherwise noted, reactions were carried out with **1** (0.1 mmol), **4** (0.1 mmol) and TMG (0.1 mmol) in 2 mL of 1,4-dioxane at rt for 4 h; ^b^ Determined by ^1^H-NMR spectroscopy of the crude reaction mixture; ^c^ Isolated yields; ^d^ Reaction time was 36 h.

**Table 4:**
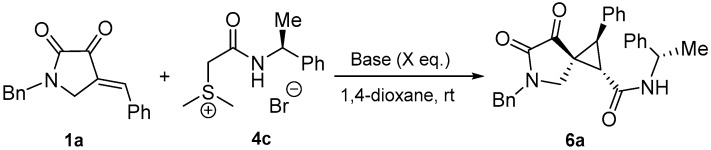
Optimization of reaction condition for the synthesis of chiral spirocyclopropane **6a** ^a^.

Entry	Base	X (eq.)	Time	d.r. ^b^	Yield (%) ^c^
1	TMG	1.0	24 h	72:28	99
2	DBU	1.0	24 h	62:38	99
3	KOH	1.5	15 min	65:35	99
4	*t*BuOK	1.5	10 min	68:32	95
5	K_2_CO_3_	2.0	72 h	68:32	92

^a^ Unless otherwise noted, reaction was carried out with **1** (0.1 mmol), **4c** (0.1 mmol) and corresponding base in 2 mL of 1,4-dioxane at r.t.; ^b^ Determined by ^1^H-NMR spectroscopy of the crude reaction mixture; ^c^ Isolated yields of both diastereoisomers.

**Table 5:**
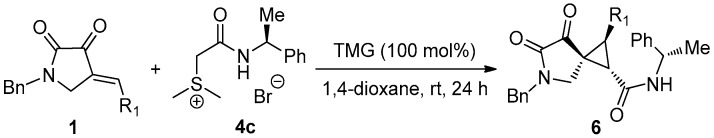
Substrates scope of cyclopropanation of cyclic enones **1** with chiral sulfonium salt **4c** ^a^.

Entry	R^1^	Product	d.r. ^b^	Yield(%) ^c^
1	Ph	**6a**	72:28	99
2	4-MeC_6_H_4_	**6b**	70:30	97
3	4-MeOC_6_H_4_	**6c**	64:36	91
4	4-FC_6_H_4_	**6d**	81:19	92
5 ^d^	2-ClC_6_H_4_	**6e**	70:30	90
6	4-BrC_6_H_4_	**6f**	62:38	81
7 ^e^	1-Naphthyl	**6g**	72:28	97
8 ^e^	2-Naphthyl	**6h**	77:23	99
9 ^e^	2-Thienyl	**6i**	60:40	90

^a^ Unless otherwise noted, reaction was carried out with **1** (0.1 mmol), **4** (0.1 mmol) and TMG (0.1 mmol) in 2 mL of 1,4-dioxane at r.t. for 24 h; ^b^ Determined by ^1^H-NMR spectroscopy of the crude reaction mixture; ^c^ Isolated yields of both diastereoisomers; ^d^ The absolute configuration of **6e** was determined by X-ray analysis. Other products were assigned by analogy; ^e^ Reaction time was 36 h.

## Supplementary Materials

Supplementary Files are available online.

## References

[1] Rajesh S.M., Perumal S., Menéndez J.C., Yogeeswari P., Sriram D. (2011). Antimycobacterial activity of spirooxindolo-pyrrolidine, pyrrolizine and pyrrolothiazole hybrids obtained by a three-component regio- and stereoselective 1,3-dipolar cycloaddition. Med. Chem. Commun..

[2] Saha S., Acharya C., Pal U., Chowdhury S.R., Sarkar K., Maiti N.C., Jaisankar P., Majumder H.K. (2016). A Novel spirooxindole derivative inhibits the growth of leishmania donovani parasites both in vitro and in vivo by targeting type IB topoisomerase. Antimicrob. Agents Chemother..

[3] Huang C.-Y., Chang C.-W., Tseng Y.-J., Lee J., Sung P.-J., Su J.-H., Hwang T.-L., Dai C.-F., Wang H.-C., Sheu J.-H. (2016). Bioactive steroids from the formosan soft coral umbellulifera petasites. Mar. Drugs.

[4] Xie J., Xing X.-Y., Sha F., Wu Z.-Y., Wu X.-Y. (2016). Enantioselective synthesis of spiro[indoline-3,4′-pyrano[2,3-*c*]pyrazole] derivatives via an organocatalytic asymmetric Michael/cyclization cascade reaction. Org. Biomol. Chem..

[5] Zhang J.-X., Wang H.-Y., Jin Q.-W., Zheng C.-W., Zhao G., Shang Y.-J. (2016). Thiourea-Quaternary Ammonium salt catalyzed asymmetric 1, 3-dipolar cycloaddition of imino esters to construct spiro[pyrrolidin-3,3′-oxindoles]. Org. Lett..

[6] Qiu Y., Yang B., Zhu C., Bäckvall J.E. (2016). Highly efficient cascade reaction for selective formation of spirocyclobutenes from dienallenes via palladium-catalyzed oxidative double carbocyclization-carbonylation-alkynylation. J. Am. Chem. Soc..

[7] Schobert R., Knauer S., Seibt S., Biersack B. (2011). Anticancer active illudins: Recent developments of a potent alkylating compound class. Curr. Med. Chem..

[8] McMorris T.C., Cong Q., Kelner M.J. (2003). Structure−activity relationship studies of illudins: Analogues possessing a spiro-cyclobutane ring. J. Org. Chem..

[9] McMorris T.C., Kelner M.J., Wang W., Diaz M.A., Estes L.A., Taetle R. (1996). Acylfulvenes, a new class of potent antitumor agents. Experientia.

[10] Tanasova M., Sturla S.J. (2012). Chemistry and biology of acylfulvenes: Sesquiterpene-derived antitumor agents. Chem. Rev..

[11] Santos C., Ferreirinha P., Sousa H., Ribeiro J., Bastos M.M., Neto T., Oliveira P.A., Medeiros R., Vilanova M., da Costa R.M.G. (2016). Ptaquiloside from bracken (*Pteridium* spp.) inhibits tumour-infiltrating CD8+ T cells in HPV-16 transgenic mice. Food Chem. Toxicol..

[12] Boger D.L., Hughes T.V., Hedrick M.P. (2001). Synthesis, chemical properties, and biological evaluation of CC-1065 and duocarmycin analogues incorporating the 5-methoxycarbonyl-1,2,9,9a-tetrahydrocyclopropa. J. Org. Chem..

[13] Boger D.L., Boyce C.W., Garbaccio R.M., Goldberg J.A. (1997). CC-1065 and the duocarmycins: Synthetic studies. Chem. Rev..

[14] Kumari R., Nguyen M.H. (2015). Fixed-dose combination of sofosbuvir and ledipasvir for the treatment of chronic hepatitis C genotype 1. Expert. Opin. Pharmacother..

[15] Younossi Z.M., Stepanova M., Marcellin P., Afdhal N., Kowdley K.V., Zeuzem S., Hunt S.L. (2015). Treatment with ledipasvir and sofosbuvir improves patient-reported outcomes: Results from the ION-1, -2, and -3 clinical trials. Hepatology.

[16] Berman H.M., Westbrook J., Feng Z., Gilliland G., Bhat T.N., Weissig H., Shindyalov I.N., Bourne P.E. (2000). The protein data bank. Nucleic Acids Res..

[17] René O., Stepek I.A., Gobbi A., Fauber B.P., Gaines S. (2015). Palladium-catalyzed ring expansion of spirocyclopropanes to form caprolactams and azepanes. J. Org. Chem..

[18] Gopalakrishnan B., Mohan S., Parella R., Babu S.A. (2016). Diastereoselective Pd(II)-catalyzed sp3 C-H arylation followed by ring opening of cyclopropanecarboxamides: Construction of anti β-Acyloxy carboxamide derivatives. J. Org. Chem..

[19] Chen H.-Y., Zhang J., Wang D.Z. (2015). Gold-catalyzed rearrangement of alkynyl donor-acceptor cyclopropanes to construct highly functionalized alkylidenecyclopentenes. Org. Lett..

[20] Fu X., Lin L.-L., Xia Y., Zhou P.-F., Liu X.-H., Feng X.-M. (2016). Catalytic asymmetric [3 + 3] annulation of cyclopropanes with mercaptoacetaldehyde. Org. Biomol. Chem..

[21] Zhang R., Liang Y.-J., Zhou G.-Y., Wang K.-W., Dong D.-W. (2008). Ring-enlargement of dimethylaminopropenoyl cyclopropanes: An efficient route to substituted 2,3-dihydrofurans. J. Org. Chem..

[22] Zhang Z.-G., Zhang W., Li J.-L., Liu Q.-F., Liu T.-X., Zhang G.-S. (2014). Synthesis of multisubstituted pyrroles from doubly activated cyclopropanes using an iron-mediated oxidation domino reaction. J. Org. Chem..

[23] Rösner C., Hennecke U. (2015). Homohalocyclization: Electrophilic bromine-induced cyclizations of cyclopropanes. Org. Lett..

[24] Tsunoi S., Maruoka Y., Suzuki I., Shibata I. (2015). Catalytic [3 + 2] cycloaddition through ring cleavage of simple cyclopropanes with isocyanates. Org. Lett..

[25] Kaicharla T., Roy T., Thangaraj M., Gonnade R.G., Biju A.T. (2016). Lewis acid catalyzed selective reactions of donor-acceptor cyclopropanes with 2-naphthols. Angew. Chem. Int. Ed..

[26] Nambu H., Ono N., Yakura T. (2016). Acid-catalyzed ring-opening cyclization of spirocyclopropanes for the construction of a 2-arylbenzofuran skeleton: Total synthesis of cuspidan B. Synthesis.

[27] Nambu H., Fukumoto M., Hirota W., Yakura T. (2014). Ring-opening cyclization of cyclohexane-1,3-dione-2-spirocyclopropanes with amines: Rapid access to 2-substituted 4-hydroxyindole. Org. Lett..

[28] Chanthamath S., Iwasa S. (2016). Enantioselective cyclopropanation of a wide variety of olefins catalyzed by Ru(II)-Pheox complexes. Acc. Chem. Res..

[29] Schröder F. (2014). Present and future of cyclopropanations in fragrance chemistry. Chem. Biodivers..

[30] Lebel H., Marcoux J.F., Molinaro C., Charette A.B. (2003). Stereoselective cyclopropanation reactions. Chem. Rev..

[31] Donaldson W.A. (2001). Synthesis of cyclopropane containing natural products. Tetrahedron.

[32] Shchepin V.V., Stepanyan Y.G., Silaichev P.S., Ezhikova M.A., Kodess M.I. (2010). Reactions of bromine-containing organozinc compounds derived from α,α-dibromo ketones with 2-arylmethylideneindan-1,3-diones and 5-arylmethylidene-2,2-dimethyl-1,3-dioxane-4,6-diones. Russ. J. Org. Chem..

[33] Russo A., Meninno S., Tedesco C., Lattanzi A. (2011). Synthesis of Activated Cyclopropanes by an MIRC Strategy: An enantioselective organocatalytic approach to spirocyclopropanes. Eur. J. Org. Chem..

[34] Wang G.-W., Gao J. (2009). Selective formation of spiro dihydrofurans and cyclopropanes through unexpected reaction of aldehydes with 1,3-dicarbonyl compounds. Org. Lett..

[35] Yoshida J.-I., Yamamoto M., Kawabata N. (1985). Electrochemical reduction of dibromodiketones facile [3 + 2] cycloaddition with olefins. Tetrahedron Lett..

[36] Qian P., Du B.-N., Song R.-C., Wu X.-D., Mei H.-B., Han J.-L., Pan Y. (2016). *N*-Iodosuccinimide-initiated spirocyclopropanation of styrenes with 1,3-dicarbonyl compound for the synthesis of spirocyclopropanes. J. Org. Chem..

[37] Lautens M., Klute W., Tam W. (1996). Transition metal-mediated cycloaddition reactions. Chem. Rev..

[38] Young I.S., Qiu Y.-P., Smith M.J., Hay M.B., Doubleday W.W. (2016). Preparation of a tricyclopropylamino acid derivative via Simmons–Smith cyclopropanation with downstream intramolecular aminoacetoxylation for impurity control. Org. Process Res. Dev..

[39] Lévesque E., Goudreauand S.R., Charette A.B. (2014). Improved zinc-catalyzed Simmons-Smith reaction: Access to various 1,2,3-trisubstituted cyclopropanes. Org. Lett..

[40] Lu T., Hayashi R., Hsung R.P., DeKorver K.A., Lohse A.G., Song Z., Tang Y. (2009). Synthesis of amido-spiro[2.2]pentanes via Simmons-Smith cyclopropanation of allenamides. Org. Biomol. Chem..

[41] Davies S.G., Ling K.B., Roberts P.M., Russell A.J., Thomson J.E. (2007). Diastereoselective Simmons-Smith cyclopropanations of allylic amines and carbamates. Chem. Commun..

[42] Pellissier H. (2008). Recent developments in asymmetric cyclopropanation. Tetrahedron.

[43] Su Y., Li Q.-F., Zhao Y.-M., Gu P.-M. (2016). Preparation of optically active cis-cyclopropane carboxylates: Cyclopropanation of α-silyl stryenes with aryldiazoacetates and desilylation of the resulting silyl cyclopropanes. Org. Lett..

[44] Rosenfeld M.J., Shankar B.K.R., Shechter H. (1988). Rhodium(II) acetate-catalyzed reactions of 2-diazo-1,3-indandione and 2-diazo-1-indanone with various substrates. J. Org. Chem..

[45] Su Y., Bai M., Qiao J.-B., Li X.-J., Li R., Tu Y.-Q., Gu P.-M. (2015). Diastereo- and enantioselective cyclopropanation of alkyenyl fluorides with benzyl diazoarylacetates. Tetrahedron Lett..

[46] Arai S., Nakayama K., Hatano K., Shioiri T. (1998). Stereoselective synthesis of cyclopropane rings under phase-transfer-catalyzed conditions. J. Org. Chem..

[47] Miyagawa T., Tatenuma T., Tadokoro M., Satoh T. (2008). A short and stereoselective synthesis of highly substituted cyclopropanes from α,β-unsaturated carbonyl compounds with dichloromethyl *p*-tolyl sulfoxide. Tetrahedron.

[48] Elinson M.N., Feducovich S.K., Vereshchagin A.N., Gorbunov S.V., Belyakov P.A., Nikishin G.I., Wang Q.-F., Song X.-K., Chen J., Yan C.-G. (2009). Pyridinium ylide-assisted one-pot two-step tandem synthesis of polysubstituted cyclopropanes. J. Comb. Chem..

[49] Li A.-H., Dai L.-X., Aggarwal V.K. (1997). Asymmetric ylide reactions: Epoxidation, cyclopropanation, aziridination, olefination, and rearrangement. Chem. Rev..

[50] McGarrigle E.M., Myers E.L., Illa O., Shaw M.A., Riches S.L., Aggarwal V.K. (2007). Chalcogenides as organocatalysts. Chem. Rev..

[51] Luo J., Wu B., Chen M.-W., Jiang G.-F., Zhou Y.-G. (2014). The concise synthesis of spiro-cyclopropane compounds via the dearomatization of indole derivatives. Org. Lett..

[52] Yuan Z.-B., Fang X.-X., Li X.-Y., Wu J., Yao H.-Q., Lin A.-J. (2015). 1,6-Conjugated addition-mediated [2 + 1] annulation: Approach to spiro[2.5]octa-4,7-dien-6-one. J. Org. Chem..

[53] Zhang S., Hu X.-Q., Wang Z.-Y., Xu P.-F. (2015). Reactions of sulfonium salts with 2,3-dioxopyrrolidine derivatives: A concise synthesis of spirocyclopropane. Synthesis.

[54] Nambu H., Ono N., Hirota W., Fukumoto M., Yakura T. (2016). An efficient method for the synthesis of 2′,3′-nonsubstituted cycloalkane-1,3-dione-2-spirocyclopropanes using (2-bromoethyl)diphenylsulfonium trifluoromethanesulfonate. Chem. Pharm. Bull. (Tokyo).

[55] Nambu H., Fukumoto M., Hirota W., Ono N., Yakura T. (2015). An efficient synthesis of cycloalkane-1,3-dione-2-spirocyclopropanes from 1,3-cycloalkanediones using (1-aryl-2-bromoethyl)-dimethylsulfonium bromides: Application to a one-pot synthesis of tetrahydroindol-4(*5H*)-one. Tetrahedron Lett..

[56] Liao W.-W., Li K., Tang Y. (2003). Controllable diastereoselective cyclopropanation. Enantioselective synthesis of vinylcyclopropanes via chiral telluronium ylides. J. Am. Chem. Soc..

[57] Zheng J.-C., Liao W.-W., Tang Y., Sun X.-L., Dai L.-X. (2005). The michael addition-elimination of ylides to α,β-unsaturated imines. Highly stereoselective synthesis of vinylcyclopropanecarbaldehydes and vinylcyclopropylaziridines. J. Am. Chem. Soc..

[58] Cao W.-G., Zhang H., Chen J., Zhou X.-H., Shao M., McMills M.C. (2008). Stereoselective synthesis of highly substituted *trans*-2,3-dihydrofuran and *trans*-1,2-cyclopropane derivatives containing sulfonyl groups. Tetrahedron.

[59] Wu X.-Y., Cao W.-G., Zhang H., Chen J., Jiang H.-Y., Deng H.-M., Shao S., Zhang J.-P., Chen H.-Y. (2008). Highly stereoselective synthesis of β,γ-disubstituted and α,β,γ-trisubstituted butyrolactones. Tetrahedron.

[60] Zhao Y.-H., Zheng C.-W., Zhao G., Cao W.-G. (2008). Highly enantioselective tandem cyclopropanation/Wittig reaction of α,β-unsaturated aldehydes with arsonium ylides catalyzed by recyclable dendritic catalyst. Tetrahedron Asymmetry.

[61] Cao W.-G., Zhang H., Chen J., Deng H.-M., Shao M., Lei L., Qian J.-X., Zhu Y. (2008). A facile preparation of *trans*-1,2-cyclopropanes containing *p*-trifluoromethylphenyl group and its application to the construction of pyrazole and cyclopropane ring fused pyridazinone derivatives. Tetrahedron.

[62] Papageorgiou C.D., Cubillo de Dios M.A., Ley S.V., Gaunt M.J. (2004). Enantioselective organocatalytic cyclopropanation via ammonium ylides. Angew. Chem. Int. Ed..

[63] Vanecko J.A., Wan H., West F.G. (2006). Recent advances in the Stevens rearrangement of ammonium ylides. Application to the synthesis of alkaloid natural products. Tetrahedron.

[64] Jończyk A., Konarska A. (1999). Generation and reactions of ammonium ylides in basic two-phase systems: Convenient synthesis of cyclopropanes, oxiranes and alkenes substituted with electron-withdrawing groups. Synlett.

[65] Kowalkowska A., Sucholbiak D., Jończyk A. (2005). Generation and reaction of ammonium ylides in basic two-phase systems. Eur. J. Org. Chem..

[66] Li J.-L., Yang K.-C., Li Y., Li Q., Zhu H.-P., Han B., Peng C., Zhi Y.-G., Gou X.-J. (2016). Asymmetric synthesis of bicyclic dihydropyrans via organocatalytic inverse-electron-demand oxo-Diels-Alder reactions of enolizable aliphatic aldehydes. Chem. Commun..

[67] Li J.-L., Li Q., Yang K.-C., Li Y., Zhou L., Han B., Peng C., Gou X.-J. (2016). A practical green chemistry approach to synthesize fused bicyclic *4H*-pyranes via an amine catalysed 1,4-addition and cyclization cascade. RSC Adv..

[68] Kaufmann D., West P.J., Smith M.D., Yagen B., Bialer M., Devor M., White H.S., Brennan K.C. (2016). sec-Butylpropylacetamide (SPD), a new amide derivative of valproic acid for the treatment of neuropathic and inflammatory pain. Pharmacol. Res..

[69] Mathieson S.R., Livingstone V., Low E., Pressler R., Rennie J.M., Boylan G.B. (2016). Phenobarbital reduces EEG amplitude and propagation of neonatal seizures but does not alter performance of automated seizure detection. Clin. Neurophysiol..

[B70-molecules-22-00328] 70.CCDC 1478324 (**3n**) and CCDC 1524538 (**6e**) Contain the Supplementary Crystallographic Data for This Paper. These Data can Be Obtained Free of Charge from the Cambridge Crystallographic Data Centre via www.ccdc.cam.ac.uk/data_request/cif.

[71] Krell E. (1963). Handbook of Laboratory Distillation.

[72] Rosengart M.J. (1954). The Technique of Distillation and Rectification in the Laboratory.

[73] Stage H. (1947). Columns for laboratory distillation. Angew. Chem..

[74] Southwick P.L., Barnas E.F. (1962). 4-Benzylidine-2,3-dioxopyrrolidines and 4-benzyl-2,3-dioxopyrrolidines. synthesis and experiments on reduction and alkylation. J. Org. Chem..

