# Supplementary material for: Diastereoselective Synthesis of Spirocyclopropanes under Mild Conditions via Formal [2 + 1] Cycloadditions Using 2,3-Dioxo-4-benzylidene-pyrrolidines
Source: Molecules. 2017 Feb 22;22(2):328. doi: 10.3390/molecules22020328 (PMC6155796; doi:10.3390/molecules22020328)
Supplement: Supplementary file 1 [file molecules-22-00328-s001.pdf]

Diastereoselective Synthesis of Spirocyclopropanes under Mild Conditions via Formal [2 + 1] Cycloadditions Using 2,3-Dioxo-4-benzylidene-pyrrolidines

Yi Li, Qing-Zhu Li,* Li Huang, Hong Liang, Kai-Chuan Yang, Hai-Jun Leng, Yue Liu, Xu-Dong Shen, Xiao-Jun Gou* and Jun-Long Li*

Antibiotics Research and Re-evaluation Key Laboratory of Sichuan Province, Sichuan Industrial Institute of Antibiotics, Chengdu University, Chengdu 610052, PR China; siia@cdu.edu.cn

** Correspondence: lqz_0519@163.com (Qing-Zhu Li); gouxj@163.com (Xiao-Jun Gou); lijunlong709@hotmail.com (Jun-Long Li); Tel.: +86-139-8223-5594 (Qing-Zhu Li); +86-028-84216087 (Xiao-Jun Gou); +86-183-8226-5469 (Jun-Long Li).*

Supporting Information

Table of Contents

1. General Information

2. General Procedure for the Synthesis of the Spirocyclopropane **3**

3. General Procedure for the Synthesis of the Multi-substituted Spirocyclopropane **5**

4. General Procedure for the Synthesis of the Multi-substituted Chiral Spirocyclopropane **6**

5. Crystal Data and Structure Refinement for the Representative Product **3n**

6. Crystal Data and Structure Refinement for the Representative Product **6e**

7. References and Notes

8. NMR Spectra of the Multi-substituted Spirocyclopropanes

1. General Information

General Procedures. All reactions were performed in oven-dried or flame-dried reaction vessels, modified Schlenk flasks, or round-bottom flasks. The flasks were fitted with Teflon screw caps and reactions were conducted under an atmosphere of argon if needed. Gas-tight syringes with stainless steel needles were used to transfer air- and moisture-sensitive liquids. All moisture and/or air sensitive solid compounds were manipulated inside normal desiccators. Flash column chromatography was performed using silica gel (40–63 μm, 230–400 mesh).

Analytical thin layer chromatography (TLC) was performed on silica gel 60 F_{254} aluminum plates (Merck) containing a 254 nm fluorescent indicator. TLC plates were visualized by exposure to short wave ultraviolet light (254 nm) and to a solution of $KMnO_4$ (1 g of $KMnO_4$, 6 g of K_2CO_3 and 0.1 g of KOH in 100 mL of H_2O) or vanillin (2 g of vanillin and 4 mL of concentrated H_2SO_4 in 100 mL of EtOH) followed by heating.

Organic solutions were concentrated at 30-50 ℃ on rotary evaporators at ~10 torr followed by drying on vacuum pump at ~1 torr. Reaction temperatures are reported as the temperature of the bath surrounding the vessel unless otherwise stated.

Materials. Commercial reagents and solvents were obtained from Adamas-beta, Aldrich Chemical Co., Alfa Aesar, Macklin and Energy Chemical and used as received with the following exceptions: THF, and toluene were purified by refluxing over Na-benzophenone under positive argon pressure followed by distillation [1-3]. The enone substrates were prepared according to literature procedure [4].

Instrumentation.

- Proton nuclear magnetic resonance (^{1}H NMR) spectra were recorded with Bruker AV 400 MHz spectrometers. Proton chemical shifts are reported in parts per million (δ scale), and are referenced using residual protium in the NMR solvent ($CDCl_3$: δ 7.26 ($CHCl_3$)). Data are reported as follows: chemical shift [multiplicity (s = singlet, d = doublet, t = triplet, q = quartet, m = multiplet, br s = broad singlet), coupling constant(s) (Hz), integration].
- Carbon-13 nuclear magnetic resonance (^{13}C NMR) spectra were recorded with Bruker AV 400 MHz spectrometers. Carbon chemical shifts are reported in parts per million (δ scale), and are referenced using the carbon resonances of the solvent (δ 77.01 ($CHCl_3$)). Data are reported as follows: chemical shift [multiplicity (if not singlet), assignment (C_q = fully substituted carbon)].
- High resolution mass spectra (HRMS) were recorded on a Waters SYNAPT G2 using an electrospray (ESI) ionization source.

2. **General Procedure for the Synthesis of multi-substituted spirocyclopropane 3.**

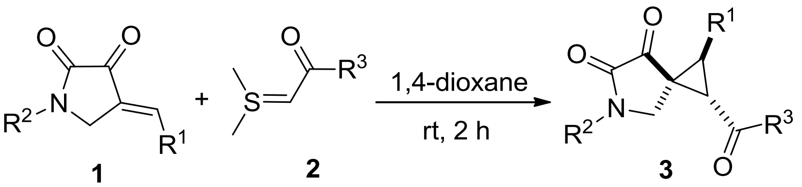

A dried glass tube was charged with cyclic enone **1** (0.1 mmol) and sulfur ylide **2** (0.1 mmol) in 1,4-dioxane (0.5 M, 2 mL). The reaction was sealed with a Teflon cap and stirred at room temperature for about 2 hours. When the reaction was complete, the reaction mixture was concentrated and the residue was purified by flash chromatography on silica gel (petroleum ether/ethyl acetate) to afford the corresponding spirocyclopropane **3**, which was dried under vacuum oven and further analyzed by ^{1}H-NMR, ^{13}C-HMR, HRMS, *etc*.

1-Benzoyl-5-benzyl-2-phenyl-5-azaspiro[2.4]heptane-6,7-dione 3a

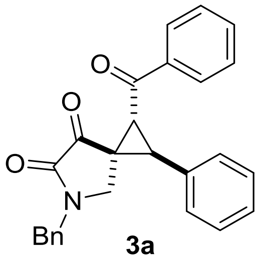

3a

Prepared according to the general procedure using 1-benzyl-4-benzylidenepyrrolidine-2,3-dione **1a** (27.7 mg, 0.1 mmol, 1.0 equiv) and sulfur ylide **2a** (18.0 mg, 0.1 mmol, 1.0 equiv). Purification of the crude product via flash chromatography on silica gel (petroleum ether/ethyl acetate = 4:1) to afford the corresponding **3a** as a white solid with 98:2 dr, 92% yield.

Characterization data for the product **3a***:*

^{1}H NMR (400 MHz, CDCl$_3$): δ (ppm): 8.05 – 7.99 (m, 2H), 7.69 – 7.60 (m, 1H), 7.52 (t, *J* = 7.7 Hz, 2H), 7.41 – 7.28 (m, 7H), 7.28 – 7.21 (m, 3H), 4.84 – 4.66 (dd, *J* = 14.4 Hz, 2H), 4.23 (d, *J* = 7.2 Hz, 1H), 3.88 (d, *J* = 12.2 Hz, 1H), 3.69 (d, *J* = 12.1 Hz, 1H), 3.59 (d, *J* = 7.2 Hz, 1H).

^{13}C NMR (100 MHz, CDCl$_3$): δ (ppm): 194.9, 193.4, 159.2, 136.6, 134.5, 134.2, 131.7, 129.0, 129.0, 129.0, 128.6, 128.4, 128.4, 128.4, 128.1, 48.7, 47.3, 44.3, 41.1, 36.6.

HR-MS (ESI): *m/z* calculated for $C_{21}H_{17}N_3O_2Na^+$: 366.1218, found: 366.1227.

1-Benzoyl-5-benzyl-2-(m-tolyl)-5-azaspiro[2.4]heptane-6,7-dione 3b

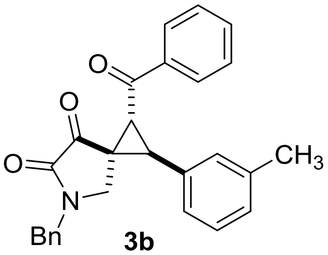

3b

Prepared according to the general procedure using 1-benzyl-4-(3-methylbenzylidene)pyrrolidine-2,3-dione **1b** (29.1 mg, 0.1 mmol, 1.0 equiv) and sulfur ylide **2a** (18.0mg, 0.1 mmol, 1.0 equiv). Purification of the crude product via flash chromatography on silica gel (petroleum ether/ethyl acetate = 4:1) to afford the corresponding **3b** as a white solid with 92:8 dr, 83% yield.

Characterization data for the product **3b**:

^{1}H NMR (400 MHz, CDCl$_3$): δ (ppm): 8.06 – 7.99 (m, 2H), 7.68 – 7.61 (m, 1H), 7.52 (t, *J* = 7.8 Hz, 2H), 7.40 – 7.28 (m, 5H), 7.20 (t, *J* = 8.0 Hz, 1H), 7.06 (m, 3H), 4.74 (q, *J* = 14.4 Hz, 2H), 4.22 (d, *J* = 7.2 Hz, 1H), 3.87 (d, *J* = 12.4 Hz, 1H), 3.68 (d, *J* = 12.4 Hz, 1H), 3.55 (d, *J* = 7.2 Hz, 1H), 2.33 (s, 3H).

^{13}C NMR (100 MHz, CDCl$_3$): δ (ppm): 194.9, 193.4, 159.3, 138.1, 136.6, 134.5, 134.2, 131.6, 129.6, 129.0, 129.0, 128.9, 128.7, 128.6, 128.4, 128.3, 126.0, 48.7, 47.4, 44.4, 41.1, 36.6, 21.3.

HR-MS (ESI): *m/z* calculated for $C_{27}H_{23}NO_3Na^+$: 432.1576, found: 432.1573.

1-Benzoyl-5-benzyl-2-(p-tolyl)-5-azaspiro[2.4]heptane-6,7-dione 3c

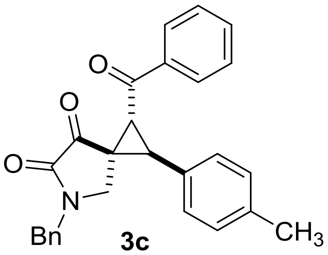

3c

Prepared according to the general procedure using 1-benzyl-4-(4-methylbenzylidene)pyrrolidine-2,3-dione **1c** (29.1 mg, 0.1 mmol, 1.0 equiv) and sulfur ylide **2a** (18.0 mg, 0.1 mmol, 1.0 equiv). Purification of the crude product via flash chromatography on silica gel (petroleum ether/ethyl acetate = 4:1) to afford the corresponding **3c** as a white solid with 98:2 dr, 93% yield.

Characterization data for the product **3c**:

^{1}H NMR (400 MHz, CDCl$_3$): δ (ppm): 8.10 – 7.86 (m, 2H), 7.68 – 7.60 (m, 1H), 7.52 (t, *J* = 7.8 Hz, 2H), 7.41 – 7.28 (m, 5H), 7.18 – 7.04 (m, 4H), 4.78 (d, *J* = 14.4 Hz, 1H), 4.69 (d, *J* = 14.4 Hz, 1H), 4.20 (d, *J* = 7.2 Hz, 1H), 3.87 (d, *J* = 12.0 Hz, 1H), 3.68 (d, *J* = 12.0 Hz, 1H), 3.55 (d, *J* = 7.2 Hz, 1H), 2.31 (s, 3H).

^{13}C NMR (100 MHz, CDCl$_3$): δ (ppm): 195.0, 193.4, 159.3, 137.9, 136.6, 134.5, 134.2, 129.1, 129.0, 129.0, 128.8, 128.7, 128.6, 128.6, 128.3, 48.7, 47.3, 44.3, 41.2, 36.6, 21.1.

HR-MS (ESI): *m/z* calculated for $C_{27}H_{23}NO_3Na^+$: 432.1576, found: 432.1579.

1-Benzoyl-5-benzyl-2-(2-methoxyphenyl)-5-azaspiro[2.4]heptane-6,7-dione 3d

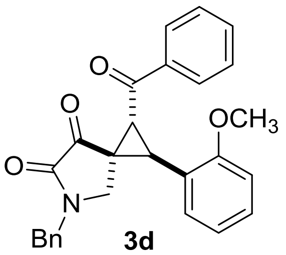

3d

Prepared according to the general procedure using 1-benzyl-4-(2-methoxybenzylidene)pyrrolidine-2,3-dione **1d** (30.7 mg, 0.1 mmol, 1.0 equiv) and sulfur ylide **2a** (18.0 mg, 0.1 mmol, 1.0 equiv). Purification of the crude product via flash chromatography on silica gel (petroleum ether/ethyl acetate = 4:1) to afford the corresponding **3d** as a white solid with 92:8 dr, 83% yield.

Characterization data for the product **3d***:*

^{1}H NMR (400 MHz, CDCl$_3$): δ (ppm): 8.11 – 7.99 (m, 2H), 7.70 – 7.61 (m, 1H), 7.57 – 7.48 (m, 2H), 7.43 – 7.31 (m, 5H), 7.30 – 7.21 (m, 2H), 6.96 (m, 1H), 6.78 (dd, *J* = 8.4, 0.8 Hz, 1H), 5.01 (d, *J* = 14.4 Hz, 1H), 4.54 (d, *J* = 14.4 Hz, 1H), 4.04 (d, *J* = 7.0 Hz, 1H), 3.95 (d, *J* = 11.8 Hz, 1H), 3.69 (d, *J* = 11.8 Hz, 1H), 3.62 (s, 3H).

^{13}C NMR (100 MHz, CDCl$_3$): δ (ppm): 195.2, 192.6, 159.8, 157.0, 136.8, 135.0, 134.1, 130.3, 130.1, 129.4, 129.0, 128.6, 128.4, 128.3, 121.2, 120.7, 110.3, 55.1, 48.5, 47.4, 39.8, 39.1, 36.7.

HR-MS (ESI): *m/z* calculated for $C_{27}H_{23}NO_4Na^+$: 448.1525, found: 448.1529.

1-Benzoyl-5-benzyl-2-(3-methoxyphenyl)-5-azaspiro[2.4]heptane-6,7-dione 3e

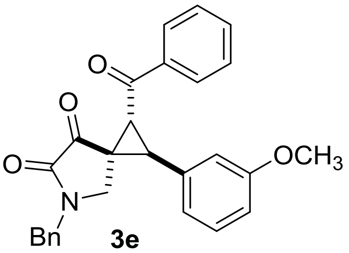

3e

Prepared according to the general procedure using 1-benzyl-4-(3-methoxybenzylidene)pyrrolidine-2,3-dione **1e** (30.7 mg, 0.1 mmol, 1.0 equiv) and sulfur ylide **2a** (18.0 mg, 0.1 mmol, 1.0 equiv). Purification of the crude product via flash chromatography on silica gel (petroleum ether/ethyl acetate = 4:1) to afford the corresponding **3e** as a white solid with 97:3 dr, 91% yield.

Characterization data for the product **3e***:*

^{1}H NMR (400 MHz, CDCl$_3$): δ (ppm): 8.05 – 7.97 (m, 2H), 7.69 – 7.60 (m, 1H), 7.52 (t, *J* = 7.6 Hz, 2H), 7.41 – 7.27 (m, 5H), 7.22 (m, 1H), 6.87 – 6.75 (m, 3H), 4.79 (d, *J* = 14.4 Hz, 1H), 4.69 (d, *J* = 14.4 Hz, 1H), 4.21 (d, *J* = 7.2 Hz, 1H), 3.87 (d, *J* = 12.0 Hz, 1H), 3.79 (s, 3H), 3.68 (d, *J* = 12.0 Hz, 1H), 3.55 (d, *J* = 7.2 Hz, 1H).

^{13}C NMR (100 MHz, CDCl$_3$): δ (ppm): 194.8, 193.4, 159.5, 159.2, 136.6, 134.5, 134.2, 133.2, 129.4, 129.0, 129.0, 128.7, 128.5, 128.4, 121.3, 115.1, 113.2, 55.2, 48.7, 47.3, 44.2, 41.1, 36.6.

HR-MS (ESI): *m/z* calculated for $C_{27}H_{23}NO_4Na^+$: 448.1525, found: 448.1523.

1-Benzoyl-5-benzyl-2-(4-methoxyphenyl)-5-azaspiro[2.4]heptane-6,7-dione 3f

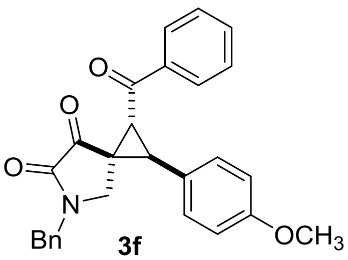

3f

Prepared according to the general procedure using 1-benzyl-4-(4-methoxybenzylidene)pyrrolidine-2,3-dione **1f** (30.7 mg, 0.1 mmol, 1.0 equiv) and sulfur ylide **2a** (18.0 mg, 0.1 mmol, 1.0 equiv). Purification of the crude product via flash chromatography on silica gel (petroleum ether/ethyl acetate = 4:1) to afford the corresponding **3f** as a white solid with 96:4 dr, 94% yield.

Characterization data for the product **3f***:*

^{1}H NMR (400 MHz, $CDCl_3$): δ (ppm): 8.06 – 7.95 (m, 2H), 7.69 – 7.60 (m, 1H), 7.52 (t, *J* = 7.6 Hz, 2H), 7.40 – 7.28 (m, 5H), 7.22 – 7.13 (m, 2H), 6.89 – 6.78 (m, 2H), 4.78 (d, *J* = 14.4 Hz, 1H), 4.69 (d, *J* = 14.4 Hz, 1H), 4.19 (d, *J* = 7.2 Hz, 1H), 3.86 (d, *J* = 12.4 Hz, 1H), 3.78 (s, 3H), 3.68 (d, *J* = 12.0 Hz, 1H), 3.54 (d, *J* = 7.2 Hz, 1H).

^{13}C NMR (100 MHz, $CDCl_3$): δ (ppm): 195.0, 193.4, 159.4, 136.6, 134.5, 134.2, 130.1, 129.1, 129.0, 128.6, 128.4, 128.4, 128.0, 123.6, 113.9, 55.3, 48.7, 47.4, 44.2, 41.3, 36.8.

HR-MS (ESI): *m/z* calculated for $C_{27}H_{23}NO_4Na^+$: 448.1525, found: 448.1521.

1-Benzoyl-5-benzyl-2-(4-fluorophenyl)-5-azaspiro[2.4]heptane-6,7-dione 3g

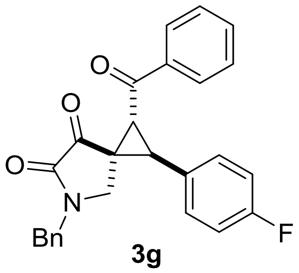

3g

Prepared according to the general procedure using 1-benzyl-4-(4-fluorophenyl)pyrrolidine-2,3-dione **1g** (29.5 mg, 0.1 mmol, 1.0 equiv) and sulfur ylide **2a** (18.0, 0.1 mmol, 1.0 equiv). Purification of the crude product via flash chromatography on silica gel (petroleum ether/ethyl acetate = 4:1) to afford the corresponding **3g** as a white solid with 96:4 dr, 88% yield.

Characterization data for the product **3g***:*

^{1}H NMR (400 MHz, $CDCl_3$): δ (ppm): 8.05 – 7.96 (m, 2H), 7.66 (t, *J* = 7.6 Hz, 1H), 7.53 (t, *J* = 7.7 Hz, 2H), 7.35 (m, 3H), 7.30 (m, 2H), 7.27 – 7.17 (m, 2H), 7.00 (t, *J* = 8.6 Hz, 2H), 4.79 (d, *J* = 14.4 Hz, 1H), 4.68 (d, *J* = 14.4 Hz, 1H), 4.17 (d, *J* = 7.2 Hz, 1H), 3.86 (d, *J* = 12.0 Hz, 1H), 3.67 (d, *J* = 12.0 Hz, 1H), 3.55 (d, *J* = 7.2 Hz, 1H).

^{13}C NMR (100 MHz, $CDCl_3$): δ (ppm): 194.6, 193.5, 159.1, 136.5, 134.4, 134.3, 130.7, 130.6, 129.1, 129.0, 128.6, 128.4, 128.4, 115.5, 115.3, 48.7, 47.2, 43.3, 41.0, 36.8.

HR-MS (ESI): *m/z* calculated for $C_{26}H_{20}FNO_3Na^+$: 436.1325, found: 436.1322.

1-Benzoyl-5-benzyl-2-(2-chlorophenyl)-5-azaspiro[2.4]heptane-6,7-dione 3h

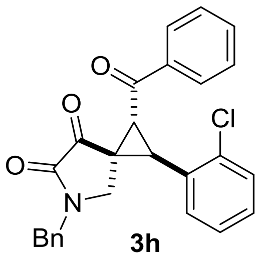

Prepared according to the general procedure using 1-benzyl-4-(2-chlorophenyl) pyrrolidine-2,3-dione **1h** (31.1 mg, 0.1 mmol, 1.0 equiv) and sulfur ylide **2a** (18.0 mg, 0.1 mmol, 1.0 equiv). Purification of the crude product via flash chromatography on silica gel (petroleum ether/ethyl acetate = 4:1) to afford the corresponding **3h** as a white solid with 97:3 dr, 98% yield.

Characterization data for the product **3h***:*

^{1}H NMR (400 MHz, $CDCl_3$): δ (ppm): 8.15 – 7.95 (m, 2H), 7.77 – 7.60 (m, 1H), 7.54 (t, *J* = 7.6 Hz, 2H), 7.41 – 7.29 (m, 7H), 7.27 – 7.20 (m, 2H), 5.06 (d, *J* = 14.4 Hz, 1H), 4.45 (d, *J* = 14.4 Hz, 1H), 4.16 (d, *J* = 6.8 Hz, 1H), 3.94 (d, *J* = 12.0 Hz, 1H), 3.67 (d, *J* = 12.0 Hz, 1H), 3.44 (d, *J* = 7.2 Hz, 1H).

^{13}C NMR (100 MHz, $CDCl_3$): δ (ppm): 194.4, 193.0, 159.2, 136.5, 135.2, 134.5, 134.4, 130.7, 130.6, 129.5, 129.4, 129.1, 129.0, 128.7, 128.4, 128.4, 126.8, 48.6, 46.5, 41.4, 39.9, 36.6.

HR-MS (ESI): *m/z* calculated for $C_{26}H_{20}ClNO_3Na^+$: 452.1029, found: 452.1028.

1-Benzoyl-5-benzyl-2-(3-chlorophenyl)-5-azaspiro[2.4]heptane-6,7-dione 3i

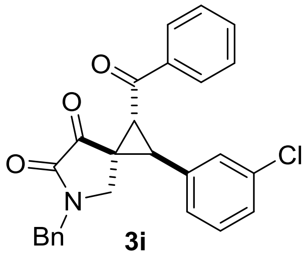

Prepared according to the general procedure using 1-benzyl-4-(3-chlorophenyl) pyrrolidine-2,3-dione **1i** (31.1 mg, 0.1 mmol, 1.0 equiv) and sulfur ylide **2a** (18.0 mg, 0.1 mmol, 1.0 equiv). Purification of the crude product via flash chromatography on silica gel (petroleum ether/ethyl acetate = 4:1) to afford the corresponding **3i** as a white solid with 97:3 dr, 94% yield.

Characterization data for the product **3i***:*

^{1}H NMR (400 MHz, $CDCl_3$): δ (ppm): 8.05 – 7.97 (m, 2H), 7.70 – 7.62 (m, 1H), 7.53 (t, *J* = 7.8 Hz, 2H), 7.41 – 7.28 (m, 5H), 7.25 (m, 3H), 7.13 (m, 1H), 4.78 (d, *J* = 14.4 Hz, 1H), 4.69 (d, *J* = 14.4 Hz, 1H), 4.18 (d, *J* = 7.2 Hz, 1H), 3.86 (d, *J* = 12.0 Hz, 1H), 3.65 (d, *J* = 12.0 Hz, 1H), 3.54 (d, *J* = 7.2 Hz, 1H).

^{13}C NMR (100 MHz, $CDCl_3$): δ (ppm): 194.4, 193.4, 159.0, 136.4, 134.4, 134.4, 134.3, 133.7, 129.6, 129.1, 129.1, 129.1, 128.6, 128.4, 128.4, 128.3, 127.2, 48.8, 47.1, 43.0, 40.8, 36.5.

HR-MS (ESI): *m/z* calculated for $C_{26}H_{20}ClNO_3Na^+$: 452.1029, found: 452.1028.

1-Benzoyl-5-benzyl-2-(4-chlorophenyl)-5-azaspiro[2.4]heptane-6,7-dione 3j

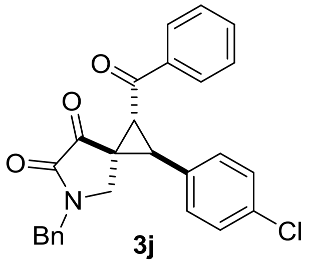

3j

Prepared according to the general procedure using 1-benzyl-4-(4-chlorophenyl) pyrrolidine-2,3-dione **1j** (31.1 mg, 0.1 mmol, 1.0 equiv) and sulfur ylide **2a** (18.0, 0.1 mmol, 1.0 equiv). Purification of the crude product via flash chromatography on silica gel (petroleum ether/ethyl acetate = 4:1) to afford the corresponding **3j** as a white solid with 96:4 dr, 59% yield.

Characterization data for the product **3j***:*

^{1}H NMR (400 MHz, $CDCl_3$): δ (ppm): 8.04 – 7.97 (m, 2H), 7.70 – 7.61 (m, 1H), 7.53 (t, *J* = 7.6 Hz, 2H), 7.35 (m, 2H), 7.32 – 7.24 (m, 5H), 7.22 – 7.15 (m, 2H), 4.79 (d, *J* = 14.4 Hz, 1H), 4.68 (d, *J* = 14.4 Hz, 1H), 4.17 (d, *J* = 7.2 Hz, 1H), 3.86 (d, *J* = 12.0 Hz, 1H), 3.66 (d, *J* = 12.0 Hz, 1H), 3.54 (d, *J* = 7.2 Hz, 1H).

^{13}C NMR (100 MHz, $CDCl_3$): δ (ppm): 194.6, 193.5, 159.1, 136.5, 134.5, 134.4, 134.1, 130.4, 130.3, 129.1, 129.1, 128.7, 128.6, 128.5, 128.4, 48.8, 47.2, 43.2, 41.0, 36.7.

HR-MS (ESI): *m/z* calculated for $C_{26}H_{20}ClNO_3Na^+$: 452.1029, found: 452.1028.

1-Benzoyl-5-benzyl-2-(3-bromophenyl)-5-azaspiro[2.4]heptane-6,7-dione 3k

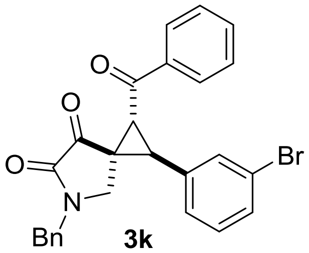

3k

Prepared according to the general procedure using 1-benzyl-4-(3-bromophenyl) pyrrolidine-2,3-dione **1k** (35.6 mg, 0.1 mmol, 1.0 equiv) and sulfur ylide **2a** (18.0 mg, 0.1 mmol, 1.0 equiv). Purification of the crude product via flash chromatography on silica gel (petroleum ether/ethyl acetate = 3:1) to afford the corresponding **3k** as a white solid with 94:6 dr, 84% yield.

Characterization data for the product **3k***:*

^{1}H NMR (400 MHz, $CDCl_3$): δ (ppm): 8.05 – 7.96 (m, 2H), 7.70 – 7.61 (m, 1H), 7.53 (t, *J* = 7.6 Hz, 2H), 7.45 – 7.38 (m, 2H), 7.35 (m, 3H), 7.30 (m, 2H), 7.21 – 7.15 (m, 2H), 4.78 (d, *J* = 14.4 Hz, 1H), 4.69 (d, *J* = 14.4 Hz, 1H), 4.18 (d, *J* = 7.2 Hz, 1H), 3.86 (d, *J* = 12.0 Hz, 1H), 3.65 (d, *J* = 12.0 Hz, 1H), 3.54 (d, *J* = 7.2 Hz, 1H).

^{13}C NMR (100 MHz, $CDCl_3$): δ (ppm): 194.4, 193.4, 159.0, 136.4, 134.4, 134.4, 134.0, 132.0, 131.3, 129.9, 129.1, 129.0, 128.6, 128.4, 128.4, 127.6, 122.4, 48.8, 47.1, 42.9, 40.8, 36.5.

HR-MS (ESI): *m/z* calculated for $C_{26}H_{20}BrNO_3Na^+$: 496.0524, found: 496.0526.

1-Benzoyl-5-benzyl-2-(4-bromophenyl)-5-azaspiro[2.4]heptane-6,7-dione 3l

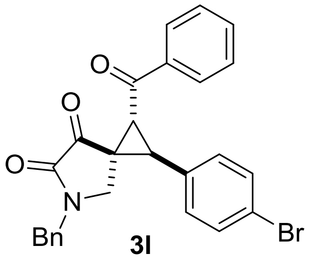

Prepared according to the general procedure using 1-benzyl-4-(4-bromophenyl) pyrrolidine-2,3-dione **1l** (35.6 mg, 0.1 mmol, 1.0 equiv) and sulfur ylide **2a** (18.0 mg, 0.1 mmol, 1.0 equiv). Purification of the crude product via flash chromatography on silica gel (petroleum ether/ethyl acetate = 3:1) to afford the corresponding **3l** as a white solid with 96:4 dr, 57% yield.

Characterization data for the product **3l***:*

^{1}H NMR (400 MHz, CDCl$_3$): δ (ppm): 8.05 – 7.95 (m, 2H), 7.66 (t, *J* = 7.4 Hz, 1H), 7.53 (t, *J* = 7.6 Hz, 2H), 7.47 – 7.41 (m, 2H), 7.35 (m, 3H), 7.30 (m, 2H), 7.13 (d, *J* = 8.4 Hz, 2H), 4.79 (d, *J* = 14.4 Hz, 1H), 4.68 (d, *J* = 14.4 Hz, 1H), 4.17 (d, *J* = 7.2 Hz, 1H), 3.86 (d, *J* = 12.0 Hz, 1H), 3.66 (d, *J* = 12.0 Hz, 1H), 3.52 (d, *J* = 7.2 Hz, 1H).

^{13}C NMR (100 MHz, CDCl$_3$): δ (ppm): 194.5, 193.5, 159.0, 136.5, 134.4, 134.4, 131.6, 130.8, 130.6, 129.1, 129.1, 128.6, 128.4, 128.4, 122.2, 48.8, 47.3, 43.2, 40.9, 36.6.

HR-MS (ESI): *m/z* calculated for $C_{26}H_{20}BrNO_3Na^+$: 496.0524, found: 496.0522.

1-Benzoyl-5-benzyl-2-(3,4-dimethoxyphenyl)-5-azaspiro[2.4]heptane-6,7-dione 3m

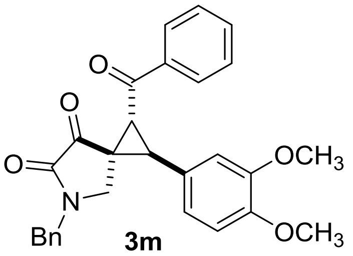

Prepared according to the general procedure using 1-benzyl-4-(3,4-dimethoxybenzylidene)pyrrolidine-2,3-dione **1m** (33.7 mg, 0.1 mmol, 1.0 equiv) and sulfur ylide **2a** (18.0 mg, 0.1 mmol, 1.0 equiv). Purification of the crude product via flash chromatography on silica gel (petroleum ether/ethyl acetate = 2:1) to afford the corresponding **3m** as a white solid with 98:2 dr, 81% yield.

Characterization data for the product **3m***:*

^{1}H NMR (400 MHz, CDCl$_3$): δ (ppm): 8.01 (m, 2H), 7.69 – 7.61 (m, 1H), 7.52 (t, *J* = 7.8 Hz, 2H), 7.40 – 7.28 (m, 5H), 6.87 – 6.76 (m, 3H), 4.79 (d, *J* = 14.4 Hz, 1H), 4.69 (d, *J* = 14.4 Hz, 1H), 4.19 (d, *J* = 7.6 Hz, 1H), 3.88 (s, 3H), 3.85 (s, 4H), 3.68 (d, *J* = 12.0Hz, 1H), 3.54 (d, *J* = 7.2 Hz, 1H),

^{13}C NMR (100 MHz, CDCl$_3$): δ (ppm): 194.8, 193.5, 159.3, 148.9, 148.7, 136.6, 134.5, 134.2, 129.0, 129.0, 128.5, 128.3, 128.3, 124.2, 121.5, 112.0, 110.9, 56.0, 55.9, 48.7, 47.3, 44.4, 41.4, 37.0.

HR-MS (ESI): *m/z* calculated for $C_{28}H_{25}NO_5Na^+$: 478.1630, found: 478.1628.

1-Benzoyl-5-benzyl-2-(2,4-dichlorophenyl)-5-azaspiro[2.4]heptane-6,7-dione 3n

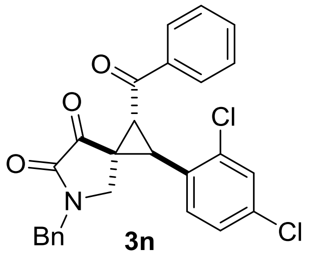

3n

Prepared according to the general procedure using 1-benzyl-4-(2,4-dichlorophenyl)pyrrolidine-2,3-dione **1n** (34.6 mg, 0.1 mmol, 1.0 equiv) and sulfur ylide **2a** (18.0 mg, 0.1 mmol, 1.0 equiv). Purification of the crude product via flash chromatography on silica gel (petroleum ether/ethyl acetate = 4:1) to afford the corresponding **3n** as a white solid with > 99:1 dr, 99% yield.

Characterization data for the product **3n***:*

^{1}H NMR (400 MHz, $CDCl_3$): δ (ppm): 8.07 – 7.98 (m, 2H), 7.72 – 7.62 (m, 1H), 7.54 (t, *J* = 7.6 Hz, 2H), 7.42 – 7.20 (m, 8H), 5.05 (d, *J* = 14.4 Hz, 1H), 4.42 (d, *J* = 14.4 Hz, 1H), 4.11 (d, *J* = 7.2 Hz, 1H), 3.91 (d, *J* = 12.0 Hz, 1H), 3.65 (d, *J* = 12.0 Hz, 1H), 3.38 (d, *J* = 7.2 Hz, 1H).

^{13}C NMR (100 MHz, $CDCl_3$): δ (ppm): 194.0, 193.0, 159.0, 136.3, 135.8, 134.7, 134.4, 131.4, 129.4, 129.2, 129.1, 128.9, 128.9, 128.7, 128.6, 128.4, 127.2, 48.6, 46.4, 40.4, 39.7, 36.4.

HR-MS (ESI): *m/z* calculated for $C_{26}H_{19}Cl_2NO_3Na^+$: 486.0640, found: 486.0641.

1-Benzoyl-5-benzyl-2-(naphthalen-1-yl)-5-azaspiro[2.4]heptane-6,7-dione 3o

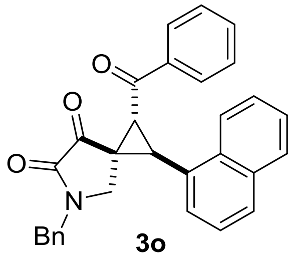

3o

Prepared according to the general procedure using 1-benzyl-4-(naphthalen-1-ylmethylene)pyrrolidine-2,3-dione **1o** (32.7 mg, 0.1 mmol, 1.0 equiv) and sulfur ylide **2a** (18.0 mg, 0.1 mmol, 1.0 equiv). Purification of the crude product via flash chromatography on silica gel (petroleum ether/ethyl acetate = 3:1) to afford the corresponding **3o** as a white solid with 94:6 dr, 94% yield.

Characterization data for the product **3o***:*

^{1}H NMR (400 MHz, $CDCl_3$): δ (ppm): 8.15 – 8.08 (m, 2H), 7.84 (d, *J* = 8.2 Hz, 1H), 7.80 (d, *J* = 8.0 Hz, 1H), 7.71 – 7.65 (m, 1H), 7.61 – 7.51 (m, 3H), 7.51 – 7.44 (m, 5H), 7.40 (m, 4H), 5.20 (d, *J* = 14.4 Hz, 1H), 4.39 (d, *J* =6.8 Hz, 2H), 4.35 (d, *J* = 14.4 Hz, 2H), 4.14 (d, *J* = 12.4 Hz, 1H), 3.85 (s, 1H), 3.82 (d, *J* = 4.8Hz, 1H).

^{13}C NMR (100 MHz, $CDCl_3$): δ (ppm): 194.9, 192.6, 159.1, 136.6, 134.8, 134.3, 133.6, 132.5, 129.2, 129.1, 129.1, 129.0, 128.6, 128.5, 128.5, 128.0, 127.0, 126.9, 126.0, 125.0, 122.2, 48.6, 47.0, 42.0, 40.4, 36.1.

HR-MS (ESI): *m/z* calculated for $C_{28}H_{25}NO_5Na^+$: 468.1576, found: 468.1573.

Ethyl-5-benzyl-6,7-dioxo-2-phenyl-5-azaspiro[2.4]heptane-1-carboxylate 3p

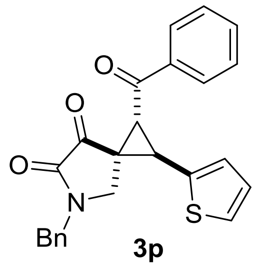

3p

Prepared according to the general procedure using 1-benzyl-4-(thiophen-2-ylmethylene)pyrrolidine-2,3-dione **1p** (28.3 mg, 0.1 mmol, 1.0 equiv) and sulfur ylide **2a** (18.0 mg, 0.1 mmol, 1.0 equiv). Purification of the crude product via flash chromatography on silica gel (petroleum ether/ethyl acetate = 3:1) to afford the corresponding **3p** as a white solid with 98:2 dr, 86% yield.

Characterization data for the product **3p***:*

^{1}H NMR (400 MHz, $CDCl_3$): δ (ppm): 8.04 – 7.95 (m, 2H), 7.68 – 7.60 (m, 1H), 7.55 – 7.47 (m, 2H), 7.39 – 7.27 (m, 5H), 7.20 (dd, *J* = 5.2, 1.2 Hz, 1H), 7.04 (m, 1H), 6.95 (m, 1H), 4.76 (d, *J* = 14.4 Hz, 1H), 4.71 (d, *J* = 14.4 Hz, 1H), 4.18 (d, *J* = 7.0 Hz, 1H), 3.85 (d, *J* = 12.0 Hz, 1H), 3.66 (d, *J* = 12.0 Hz, 1H), 3.62 (dd, *J* = 7.0, 0.8Hz, 1H).

^{13}C NMR (100 MHz, $CDCl_3$): δ (ppm): 194.2, 192.6, 159.1, 136.4, 134.4, 134.4, 134.2, 129.0, 128.9, 128.5, 128.3, 128.3, 127.5, 126.9, 125.6, 48.6, 47.0, 41.4, 38.5, 37.9.

HR-MS (ESI): *m/z* calculated for $C_{24}H_{19}NO_3SNa^+$: 424.0983, found: 424.0981.

1-Benzoyl-5-(4-methoxybenzyl)-2-phenyl-5-azaspiro[2.4]heptane-6,7-dione 3q

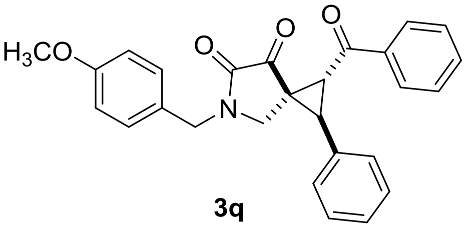

3q

Prepared according to the general procedure using *(Z)*-4-benzylidene-1-(4-methoxybenzyl)pyrrolidine-2,3-dione **1q** (30.7 mg, 0.1 mmol, 1.0 equiv) and sulfur ylide **2a** (18.0 mg, 0.1 mmol, 1.0 equiv). Purification of the crude product via flash chromatography on silica gel (petroleum ether/ethyl acetate = 4:1) to afford the corresponding **3q** as a white solid with 98:2 dr, 90% yield.

Characterization data for the product **3q***:*

^{1}H NMR (400 MHz, $CDCl_3$): δ (ppm): 8.04 – 7.98 (m, 2H), 7.69 – 7.60 (m, 1H), 7.52 (t, *J* = 8.0 Hz, 2H), 7.35 – 7.27 (m, 3H), 7.27 – 7.19 (m, 5H), 6.92 – 6.84 (m, 2H), 4.72 (d, *J* = 14.4 Hz, 1H), 4.64 (d, *J* = 14.4 Hz, 1H), 4.22 (d, *J* = 7.2 Hz, 1H), 3.86 (d, *J* = 12.0 Hz, 1H), 3.80 (s, 3H), 3.66 (d, *J* = 12.0 Hz, 1H), 3.58 (d, *J* = 7.2 Hz, 1H).

^{13}C NMR (100 MHz, $CDCl_3$): δ (ppm): 194.9, 193.6, 159.6, 159.1, 136.6, 134.2, 131.7, 130.0, 129.0, 128.4, 128.4, 128.1, 126.5, 114.4, 55.3, 48.1, 47.1, 44.2, 41.1, 36.6.

HR-MS (ESI): *m/z* calculated for $C_{27}H_{23}NO_4Na^+$: 448.1525, found: 448.1528.

Ethyl-5-benzyl-6,7-dioxo-2-phenyl-5-azaspiro[2.4]heptane-1-carboxylate 3r

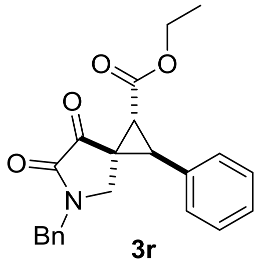

3r

Prepared according to the general procedure using 1-benzyl-4-benzylidenepyrrolidine-2,3-dione **1a** (27.7 mg, 0.1 mmol, 1.0 equiv) and (ethoxycarbonyl)dimethylsulfonium bromide **2b** (21.3 mg, 0.1 mmol, 1.0 equiv) in the presence of TMG (13μl, 0,1mmol, 1.0 equiv). Purification of the crude product via flash chromatography on silica gel (petroleum ether/ethyl acetate = 4:1) to afford the corresponding **3r** as a white solid with 92:8 dr, 99% yield.

Characterization data for the product **3r**:

^{1}H NMR (400 MHz, CDCl$_3$): δ (ppm): 7.42 – 7.34 (m, 3H), 7.34 – 7.30 (m, 2H), 7.29 (d, *J* = 1.8 Hz, 1H), 7.28 – 7.25 (m, 2H), 7.22 – 7.17 (m, 2H), 4.75 (s, 2H), 4.19 (qd, *J* = 7.2, 2.8 Hz, 2H), 3.77 (q, *J* = 12.0 Hz, 2H), 3.33 (d, *J* = 7.2 Hz, 1H), 3.21 (d, *J* = 7.2 Hz, 1H), 1.28 (t, *J* = 7.2 Hz, 3H).

^{13}C NMR (100 MHz, CDCl$_3$): δ (ppm): 192.9, 169.3, 159.1, 134.5, 131.1, 129.0, 128.9, 128.5, 128.3, 128.3, 128.0, 61.8, 48.6, 47.4, 42.9, 38.1, 33.5, 14.0.

HR-MS (ESI): *m/z* calculated for $C_{22}H_{21}NO_4Na^+$: 386.1386, found: 386.1385.

Tert-butyl-5-benzyl-6,7-dioxo-2-phenyl-5-azaspiro[2.4]heptane-1-carboxylate 3s

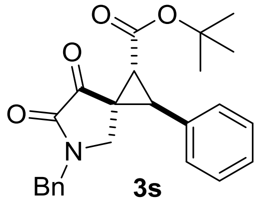

3s

Prepared according to the general procedure using 1-benzyl-4-benzylidenepyrrolidine-2,3-dione **1a** (27.7 mg, 0.1 mmol, 1.0 equiv) and (2-(tert-butoxy)-2-oxoethyl)dimethylsulfonium bromide **2c** (25.6 mg, 0.1 mmol, 1.0 equiv) in the presence of TMG (13μl, 0,1mmol, 1.0 equiv). Purification of the crude product via flash chromatography on silica gel (petroleum ether/ethyl acetate = 4:1) to afford the corresponding **3s** as a white solid with 90:10 dr, 99% yield.

Characterization data for the product **3s**:

^{1}H NMR (400 MHz, CDCl$_3$): δ (ppm): 7.30 (m, 3H), 7.28 – 7.24 (m, 2H), 7.22 (dd, *J* = 7.1, 1.8 Hz, 2H), 7.20 – 7.18 (m, 1H), 7.15 – 7.11 (m, 2H), 4.67 (s, 2H), 3.68 (d, *J* = 12.0 Hz, 1H), 3.62 (d, *J* = 12.0 Hz, 1H), 3.21 (d, *J* = 7.2 Hz, 1H), 3.05 (d, *J* = 7.2 Hz, 1H), 1.36 (s, 9H).

^{13}C NMR (100 MHz, CDCl$_3$): δ (ppm): 192.1, 167.3, 158.2, 133.5, 130.3, 128.0, 127.9, 127.5, 127.3, 127.2, 126.9, 81.8, 47.5, 46.3, 41.6, 37.0, 33.9, 27.0.

HR-MS (ESI): *m/z* calculated for $C_{24}H_{25}NO_4Na^+$: 414.1681, found: 414.1680.

3. General Procedure for the Synthesis of multi-substituted spirocyclopropane 5

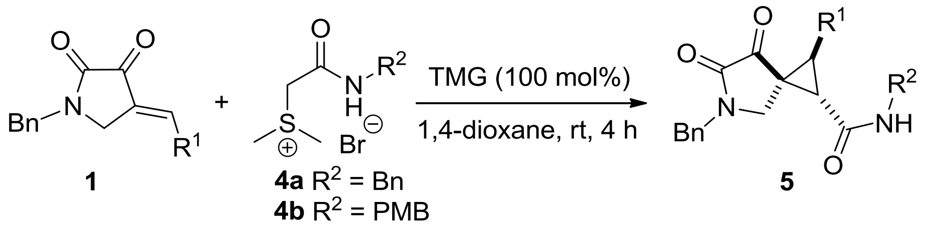

A dried glass tube was charged with cyclic enones **1** (0.1 mmol) and amidic sulfonium salt **4** (0.1 mmol) at the presence of TMG (13μl, 0,1mmol, 1.0 equiv) in 1,4-dioxane (0.5 M, 2 mL). The reaction was sealed with a Teflon cap and stirred at room temperature overnight. When the reaction was complete, the reaction mixture was concentrated and the residue was purified by flash chromatography on silica gel (petroleum ether/ethyl acetate) to afford the corresponding spirocyclopropane **5**, which was dried under vacuum oven and further analyzed by ^{1}H-NMR, ^{13}C-HMR, HRMS, *etc.*

<u>*N*,5-Dibenzyl-6,7-dioxo-2-phenyl-5-azaspiro[2.4]heptane-1-carboxamide 5a</u>

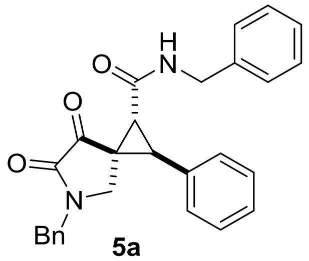

5a

Prepared according to the general procedure using 1-benzyl-4-benzylidenepyrrolidine-2,3-dione **1a** (27.7 mg, 0.1 mmol, 1.0 equiv) and (2-(benzylamino)-2-oxoethyl)dimethylsulfonium bromide **4a** (29.0 mg, 0.1 mmol, 1.0 equiv). Purification of the crude product via flash chromatography on silicagel (petroleum ether/ethyl acetate = 2:1) to afford the corresponding **5a** as a white solid with 96:4 dr, 98% yield.

Characterization data for the product **5a***:*

^{1}H NMR (400 MHz, $CDCl_3$): δ (ppm): 8.27 (s, 1H), 7.36 – 7.30 (m, 3H), 7.26 (m, 5H), 7.21 (m, 2H), 7.14 (m, 4H), 4.73 (d, *J* = 4.4 Hz, 2H), 4.48 (dd, *J* = 15.2, 6.4 Hz, 1H), 4.36 (dd, *J* = 15.2, 5.6 Hz, 1H), 3.68 (d, *J* = 5.2 Hz, 2H), 3.53 (d, *J* = 7.6 Hz, 1H), 3.15 (d, *J* = 7.2 Hz, 1H).

^{13}C NMR (100 MHz, $CDCl_3$): δ (ppm): 195.1, 167.0, 160.3, 138.1, 134.2, 132.3, 129.2, 129.1, 128.9, 128.4, 128.2, 128.2, 127.6, 127.3, 126.9, 48.7, 47.7, 43.6, 41.4, 38.1, 36.9.

HR-MS (ESI): *m/z* calculated for $C_{27}H_{24}N2O_3Na^+$: 447.1685, found: 447.1684.

<u>5-Benzyl-N-(4-methoxybenzyl)-6,7-dioxo-2-phenyl-5-azaspiro[2.4]heptane-1-carboxamide 5b</u>

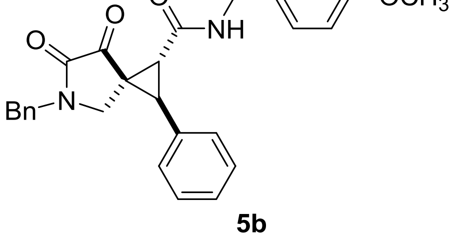

5b

Prepared according to the general procedure using 1-benzyl-4-benzylidenepyrrolidine-2,3-dione **1a** (27.7 mg, 0.1 mmol, 1.0 equiv) and (2-((4-methoxybenzyl)amino)-2-

oxoethyl)dimethylsulfonium bromide **4b** (32.0 mg, 0.1 mmol, 1.0 equiv). Purification of the crude product via flash chromatography on silica gel (petroleum ether/ethyl acetate = 2:1) to afford the corresponding **5b** as a white solid with > 99:1 dr, 99% yield.
Characterization data for the product **5b***:*
^{1}H NMR (400 MHz, $CDCl_3$): δ (ppm): 7.91 (t, *J* = 5.6 Hz, 1H), 7.27 – 7.24 (m, 3H), 7.24 – 7.16 (m, 5H), 7.10 – 7.03 (m, 4H), 6.69 – 6.60 (m, 2H), 4.67 (s, 2H), 4.32 (dd, *J* = 15.0, 6.2 Hz, 1H), 4.24 (dd, *J* = 14.8, 5.4 Hz, 1H), 3.70 (s, 3H), 3.62 (d, *J* = 8.8 Hz, 2H), 3.45 (d, *J* = 7.2 Hz, 1H), 3.03 (d, *J* = 7.6 Hz, 1H).
^{13}C NMR (100 MHz, $CDCl_3$): δ (ppm): 195.0, 166.9, 160.3, 158.6, 134.2, 132.3, 130.2, 129.2, 129.1, 128.7, 128.2, 128.2, 128.2, 127.6, 113.8, 55.2, 48.7, 47.7, 43.2, 41.4, 38.1, 37.0.
HR-MS (ESI): *m/z* calculated for $C_{28}H_{26}N_2O_4Na^+$: 477.1790, found: 477.1785.

<u>*N*,5-Dibenzyl-6,7-dioxo-2-(p-tolyl)-5-azaspiro[2.4]heptane-1-carboxamide 5c</u>

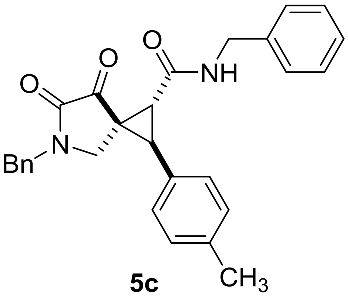

5c

Prepared according to the general procedure using 1-benzyl-4-(4-methylbenzylidene)pyrrolidine-2,3-dione **1c** (29.1 mg, 0.1 mmol, 1.0 equiv) and (2-(benzylamino)-2-oxoethyl)dimethylsulfonium bromide **4a** (29.0 mg, 0.1 mmol, 1.0 equiv). Purification of the crude product via flash chromatography on silica gel (petroleum ether/ethyl acetate = 2:1) to afford the corresponding **5c** as a white solid with 97:3 dr, 99% yield.
Characterization data for the product **5c***:*
^{1}H NMR (400 MHz, $CDCl_3$): δ (ppm): 7.92 (s, 1H), 7.27 – 7.23 (m, 3H), 7.21 – 7.20 (m, 3H), 7.16 – 7.13 (m, 2H) , 7.12 – 7.09 (m, 2H), 7.02 – 6.92 (m, 4H), 4.67 (s, 2H), 4.41 (dd, *J* = 15.2, 6.0 Hz, 1H), 4.31 (dd, *J* = 15.2, 5.6 Hz, 1H), 3.62 (q, *J* = 16.8, 12.4 Hz, 2H), 3.43 (d, *J* = 7.4 Hz, 1H), 3.02 (d, *J* = 7.6 Hz, 1H), 2.26 (s, 3H).
^{13}C NMR (100 MHz, $CDCl_3$): δ (ppm): 194.9, 167.1, 160.3, 138.0, 137.3, 134.3, 129.2, 129.1, 129.0, 128.9, 128.4, 128.3, 128.2, 127.4, 127.0, 48.7, 47.8, 43.7, 41.5, 38.3, 36.9, 21.1.
HR-MS (ESI): *m/z* calculated for $C_{28}H_{26}N_2O_3Na^+$: 461.1841, found: 461.1838.

<u>5-Benzyl-N-(4-methoxybenzyl)-6,7-dioxo-2-(p-tolyl)-5-azaspiro[2.4]heptane-1-carboxamide 5d</u>

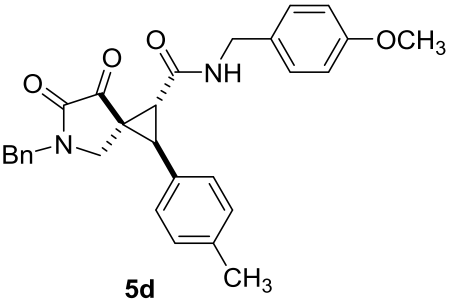

5d

Prepared according to the general procedure using 1-benzyl-4-(4-methylbenzylidene)pyrrolidine-2,3-dione **1c** (29.1 mg, 0.1 mmol, 1.0 equiv) and (2-((4-methoxybenzyl)amino)-2-oxoethyl)dimethylsulfonium bromide **4b** (32.0 mg, 0.1 mmol, 1.0 equiv). Purification of the crude product via flash chromatography on silica gel (petroleum ether/ethyl acetate = 2:1) to afford the corresponding **5d** as a white solid with 94:6 dr, 92% yield.

Characterization data for the product **5d***:*

^{1}H NMR (400 MHz, $CDCl_3$): δ (ppm): 7.97 (t, *J* = 5.8 Hz, 1H), 7.27 – 7.16 (m, 5H), 7.10 – 7.03 (m, 2H), 7.01 – 6.92 (m, 4H), 6.67 – 6.60 (m, 2H), 4.66 (s, 2H), 4.27 (qd, *J* = 15.0, 5.8 Hz, 2H), 3.69 (s, 3H), 3.67 – 3.55 (m, 2H), 3.41 (d, *J* = 7.2 Hz, 1H), 3.01 (d, *J* = 7.6 Hz, 1H).

^{13}C NMR (100 MHz, $CDCl_3$): δ (ppm): 195.0, 167.0, 160.3, 158.6, 137.3, 134.3, 130.2, 129.2, 129.1, 129.0, 128.9, 128.7, 128.2, 128.2, 113.8, 55.2, 48.6, 47.7, 43.1, 41.3, 38.2, 37.1, 21.1.

HR-MS (ESI): *m/z* calculated for $C_{29}H_{28}N_2O_4Na^+$: 491.1947, found: 491.1958.

N,5-Dibenzyl-2-(4-chlorophenyl)-6,7-dioxo-5-azaspiro[2.4]heptane-1-carboxamide 5e

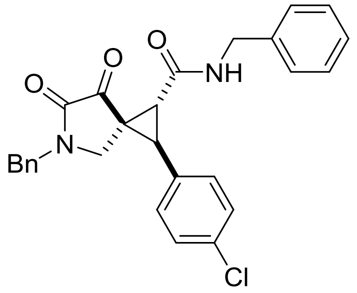

5e

Prepared according to the general procedure using 1-benzyl-4-(4-chlorophenyl) pyrrolidine-2,3-dione **1j** (31.1 mg, 0.1 mmol, 1.0 equiv) and (2-(benzylamino)-2-oxoethyl)dimethylsulfonium bromide **4a** (29.0 mg, 0.1 mmol, 1.0 equiv). Purification of the crude product via flash chromatography on silica gel (petroleum ether/ethyl acetate = 2:1) to afford the corresponding **5e** as a white solid with 91:9 dr, 99% yield.

Characterization data for the product **5e***:*

^{1}H NMR (400 MHz, $CDCl_3$): δ (ppm): 8.24 (t, *J* = 6.0 Hz, 1H), 7.40 – 7.29 (m, 3H), 7.29 – 7.24 (m, 3H), 7.24 – 7.13 (m, 6H), 7.11 – 7.01 (m, 2H), 4.80 (d, *J* = 14.4 Hz, 1H), 4.68 (d, *J* = 14.4 Hz, 1H), 4.52 (dd, J = 15.2, 6.4 Hz, 1H), 4.35 (dd, *J* = 15.2, 5.6 Hz, 1H), 3.68 (q, *J* = 15.6, 12.4 Hz, 2H), 3.50 (d, *J* = 7.4 Hz, 1H), 3.10 (d, *J* = 7.6 Hz, 1H).

^{13}C NMR (100 MHz, $CDCl_3$): δ (ppm): 195.2, 166.7, 160.2, 137.9, 134.0, 133.5, 130.8, 130.5, 129.2, 128.4, 128.4, 128.4, 128.1, 127.3, 127.1, 48.7, 47.6, 43.7, 40.4, 37.9, 37.1.

HR-MS (ESI): *m/z* calculated for $C_{27}H_{23}ClN_2O_3Na^+$: 481.1295, found: 481.1296.

5-Benzyl-2-(2-chlorophenyl)-N-(4-methoxybenzyl)-6,7-dioxo-5-azaspiro[2.4]heptane-1-carboxamide 5f

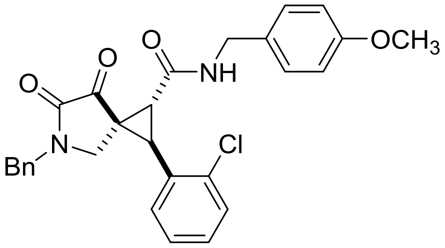

5f

Prepared according to the general procedure using 1-benzyl-4-(2-chlorophenyl) pyrrolidine-2,3-dione **1h** (31.1 mg, 0.1 mmol, 1.0 equiv) and (2-((4-methoxybenzyl)amino)-2-oxoethyl)dimethylsulfonium bromide **4b** (32.0 mg, 0.1 mmol, 1.0 equiv). Purification of the crude product via flash chromatography on silica gel (petroleum ether/ethyl acetate = 2:1) to afford the corresponding **5f** as a white solid with 94:6 dr, 98% yield.

Characterization data for the product **5f**:

^{1}H NMR (400 MHz, $CDCl_3$): δ (ppm): 8.25 – 8.08 (m, 1H), 7.27 – 7.20 (m, 4H), 7.20 – 7.11 (m, 5H), 7.10 – 7.03 (m, 2H), 6.71 – 6.63 (m, 2H), 4.88 (d, *J* = 14.4 Hz, 1H), 4.40 (d, *J* = 14.4 Hz, 1H), 4.28 (qd, *J* = 14.8, 5.6 Hz, 2H), 3.72 (s, 3H), 3.68 (d, *J* = 12.2 Hz, 1H), 3.54 (d, *J* = 12.2 Hz, 1H), 3.22 (d, *J* = 7.4 Hz, 1H), 3.00 – 2.87 (m, 1H).

^{13}C NMR (100 MHz, $CDCl_3$): δ (ppm): 194.6, 166.6, 160.2, 158.7, 135.2, 134.3, 131.4, 130.9, 130.2, 129.2, 129.0, 129.0, 129.0, 128.8, 128.6, 128.2, 126.7, 113.8, 55.2, 48.5, 47.0, 43.3, 38.9, 37.0.

HR-MS (ESI): *m/z* calculated for $C_{28}H_{25}ClN_2O_4Na^+$: 511.1401, found: 511.1401.

N,5-Dibenzyl-2-(naphthalen-2-yl)-6,7-dioxo-5-azaspiro[2.4]heptane-1-carboxamide 5g

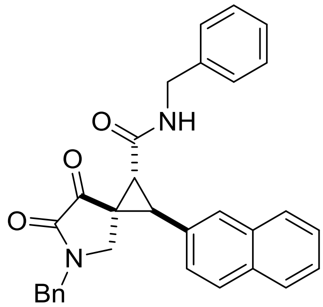

5g

Prepared according to the general procedure using 1-benzyl-4-(naphthalen-2-ylmethylene)pyrrolidine-2,3-dione **1r** (32.7 mg, 0.1 mmol, 1.0 equiv) and (2-(benzylamino)-2-oxoethyl)dimethylsulfonium bromide **4a** (29.0 mg, 0.1 mmol, 1.0 equiv). Purification of the crude product via flash chromatography on silica gel (petroleum ether/ethyl acetate = 2:1) to afford the corresponding **5g** as a white solid with > 99:1 dr, 85% yield.

Characterization data for the product **5g***:*

^{1}H NMR (400 MHz, $CDCl_3$): δ (ppm): 7.92 (t, *J* = 6.0 Hz, 1H), 7.82 – 7.80 (m, 1H), 7.76 – 7.68 (m, 1H), 7.63 (d, *J* = 1.6 Hz, 1H), 7.54 – 7.40 (m, 2H), 7.31 – 7.15 (m, 9H), 7.14 – 7.06 (m, 1H), 7.06 – 6.98 (m, 2H), 4.76 (s, 2H), 4.49 (dd, *J* = 15.2, 6.4 Hz, 1H), 4.35 (dd, *J* = 15.2, 5.2 Hz, 1H), 3.76 (d, *J* = 12.4 Hz, 1H), 3.72 – 3.62 (m, 2H), 3.22 (d, *J* = 7.6 Hz, 1H).

^{13}C NMR (100 MHz, CDCl$_3$): δ (ppm): 194.9, 167.0, 160.2, 137.9, 134.2, 133.0, 132.8, 129.8, 129.1, 128.4, 128.3, 128.3, 128.2, 128.0, 127.9, 127.6, 127.3, 127.1, 127.0, 126.3, 126.2, 48.7, 47.7, 43.7, 41.5, 38.1, 37.0.
HR-MS (ESI): *m/z* calculated for $C_{31}H_{26}N_2O_3Na^+$: 497.1841, found: 497.1841.

5-Benzyl-*N*-(4-methoxybenzyl)-2-(naphthalen-2-yl)-6,7-dioxo-5-azaspiro[2.4]heptane-1-carboxamide 5h

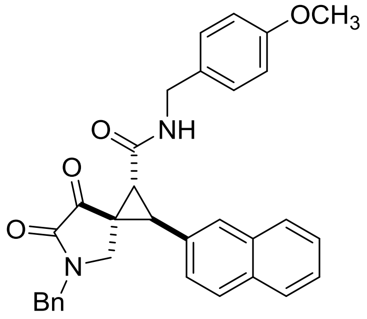

5h

Prepared according to the general procedure using 1-benzyl-4-(naphthalen-2-ylmethylene)pyrrolidine-2,3-dione **1r** (32.7 mg, 0.1 mmol, 1.0 equiv) and (2-((4-methoxybenzyl)amino)-2-oxoethyl)dimethylsulfonium bromide **4b** (32.0 mg, 0.1 mmol, 1.0 equiv). Purification of the crude product via flash chromatography on silica gel (petroleum ether/ethyl acetate = 2:1) to afford the corresponding **5h** as a white solid with > 99:1 dr, 90% yield.
Characterization data for the product **5h***:*
^{1}H NMR (400 MHz, CDCl$_3$): δ (ppm): 7.76 – 7.61 (m, 3H), 7.58 (s, 1H), 7.43 – 7.33 (m, 2H), 7.20 (d, *J* = 9.1 Hz, 7H), 7.14 – 7.12 (m, 1H), 7.09 – 7.02 (m, 2H), 6.63 (d, *J* = 8.4 Hz, 2H), 4.68 (dq, *J* = 12.6, 14.4 Hz, 2H), 4.29 (dd, *J* = 5.7, 3.3 Hz, 2H), 4.33 – 4.26 (m, 2H), 3.77 – 3.70 (m, 2H), 3.66 (s, 3H), 3.63 – 3.56 (m, 1H), 3.08 (d, *J* = 7.4 Hz, 1H).
^{13}C NMR (100 MHz, CDCl$_3$): δ (ppm): 195.0, 166.8, 160.2, 158.7, 134.2, 133.0, 132.7, 130.0, 129.8, 129.1, 128.8, 128.3, 128.2, 128.2, 127.9, 127.8, 127.6, 127.0, 126.3, 126.1, 113.8, 55.2, 48.7, 47.7, 43.3, 41.5, 38.1, 37.2.
HR-MS (ESI): *m/z* calculated for $C_{32}H_{28}N_2O_4Na^+$: 527.1947, found: 527.1943.

***N*,5-Dibenzyl-6,7-dioxo-2-(thiophen-2-yl)-5-azaspiro[2.4]heptane-1-carboxamide 5i**

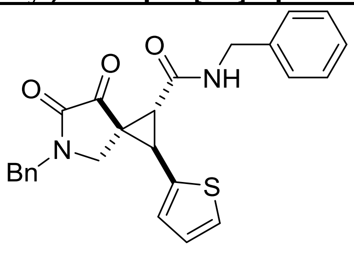

5i

Prepared according to the general procedure using 1-benzyl-4-(thiophen-2-ylmethylene)pyrrolidine-2,3-dione **1p** (28.3 mg, 0.1 mmol, 1.0 equiv) and (2-(benzylamino)-2-oxoethyl)dimethylsulfonium bromide **4a** (28.9 mg, 0.1 mmol, 1.0 equiv). Purification of the

crude product via flash chromatography on silica gel (petroleum ether/ethyl acetate = 2:1) to afford the corresponding **5i** as a white solid with > 99:1 dr, 83% yield.

Characterization data for the product **5i***:*

^{1}H NMR (400 MHz, CDCl$_3$): δ (ppm): 8.44 – 8.21 (m, 1H), 7.32 – 7.25 (m, 3H), 7.23 – 7.20 (m, 2H), 7.18 – 7.13 (m, 2H), 7.13 – 7.09 (m, 4H), 6.86 – 6.76 (m, 2H), 4.74 (d, *J* = 14.6 Hz, 1H), 4.61 (d, *J* = 14.6 Hz, 1H), 4.43 (dd, *J* = 15.2, 6.4 Hz, 1H), 4.28 (dd, *J* = 15.2, 5.4 Hz, 1H), 3.61 (s, 2H), 3.51 (d, *J* = 6.8 Hz, 1H), 3.11 – 2.97 (m, 1H).

^{13}C NMR (100 MHz, CDCl$_3$): δ (ppm): 194.3, 166.5, 160.3, 138.0, 135.2, 134.2, 129.1, 128.4, 128.3, 127.4, 127.3, 127.0, 126.7, 125.3, 48.7, 47.5, 43.7, 38.6, 38.3, 35.9.

HR-MS (ESI): *m/z* calculated for $C_{25}H_{22}N_2O_3SNa^+$: 453.1249, found: 453.1250.

5-Benzyl-N-(4-methoxybenzyl)-6,7-dioxo-2-(thiophen-2-yl)-5-azaspiro[2.4]heptane-1-carboxamide 5j

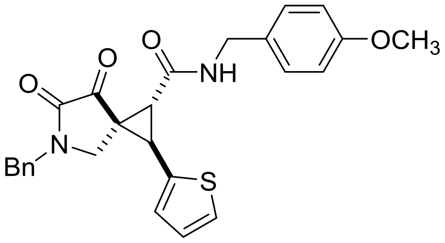

5j

Prepared according to the general procedure using 1-benzyl-4-(thiophen-2-ylmethylene)pyrrolidine-2,3-dione **1p** (28.3 mg, 0.1 mmol, 1.0 equiv) and (2-((4-methoxybenzyl)amino)-2-oxoethyl)dimethylsulfonium bromide **4b** (32.0 mg, 0.1 mmol, 1.0 equiv). Purification of the crude product via flash chromatography on silica gel (petroleum ether/ethyl acetate = 2:1) to afford the corresponding **5j** as a white solid with > 99:1 dr, 81% yield.

Characterization data for the product **5j***:*

^{1}H NMR (400 MHz, CDCl$_3$): δ (ppm): 8.20 – 8.01 (m, 1H), 7.39 – 7.32 (m, 3H), 7.32 – 7.28 (m, 2H), 7.21 – 7.11 (m, 3H), 6.92 – 6.89 (m, 1H), 6.89 – 6.82 (m, 1H), 6.77 – 6.69 (m, 2H), 4.80 (d, *J* = 14.6 Hz, 1H), 4.70 (d, *J* = 14.6 Hz, 1H), 4.42 (dd, *J* = 14.9, 6.2 Hz, 1H), 4.30 (dd, *J* = 14.8, 6.0 Hz, 1H), 3.77 (s, 3H), 3.69 (d, *J* = 1.8 Hz, 2H), 3.58 (d, *J* = 7.2, 1H), 3.07 (d, *J* = 7.2 Hz, 1H).

^{13}C NMR (100 MHz, CDCl$_3$): δ (ppm): 194.2, 166.3, 160.2, 158.7, 135.2, 134.2, 130.1, 129.1, 128.7, 128.3, 127.4, 126.7, 125.3, 113.9, 55.2, 48.7, 47.5, 43.3, 38.6, 38.3, 35.9.

HR-MS (ESI): *m/z* calculated for $C_{26}H_{24}N_2O_4Na^+$: 483.1354, found: 483.1356.

4. General Procedure for the Synthesis of multi-substituted chiral spirocyclopropane 6

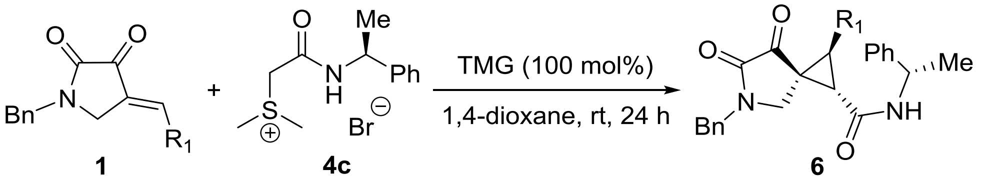

1 **4c** **6**

A dried glass tube was charged with cyclic enone **1** (0.1 mmol) and (*S*)-dimethyl(2-oxo-2-((1-phenylethyl)amino)ethyl)sulfonium bromide **4c** (0.1 mmol) at the presence of TMG (13μl, 0.1mmol, 1.0 equiv) in 1,4-dioxane (0.5 M, 2 mL). The reaction was sealed with a Teflon cap

and stirred at room temperature overnight. When the reaction was complete, the reaction mixture was concentrated and the residue was purified by flash chromatography on silica gel (methanol/dichloromethane) to afford the corresponding chiral spirocyclopropane **6**, which was dried under vacuum oven and further analyzed by ^{1}H-NMR, ^{13}C-HMR, HRMS, *etc*.

(1*S*,2*R*,3*S*)-5-Benzyl-6,7-dioxo-2-phenyl-*N*-((*S*)-1-phenylethyl)-5-azaspiro[2.4]heptane-1-carboxamide 6a

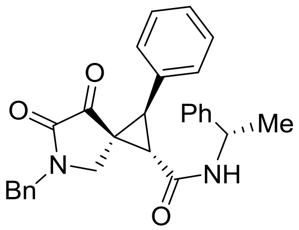

6a

Prepared according to the general procedure using 1-benzyl-4-benzylidenepyrrolidine-2,3-dione **1a** (27.7 mg, 0.1 mmol, 1.0 equiv) and (*S*)-dimethyl(2-oxo-2-((1-phenylethyl)amino)ethyl)sulfonium bromide **4c** (30.4 mg, 0.1 mmol, 1.0 equiv). Purification of the crude product via flash chromatography on silica gel (methanol/dichloromethane = 1:300) to afford the corresponding **6a** as a white solid with 72:28 dr, 99% yield, $[\alpha]_D^{20}$ = +94.6 (*c* = 0.84 in $CHCl_3$).

Characterization data for the product **6a**:

^{1}H NMR (400 MHz, $CDCl_3$): δ (ppm): 7.31 – 7.23 (m, 3H), 7.23 – 7.14 (m, 10H), 7.14 – 7.08 (m, 2H), 7.01 (d, *J* = 7.6 Hz, 1H), 5.03 – 4.86 (m, 1H), 4.73 (d, *J* = 14.4 Hz, 1H), 4.34 (d, *J* = 14.4 Hz, 1H), 3.57 (d, *J* = 12.2 Hz, 1H), 3.46 – 3.32 (m, 2H), 2.99 (d, *J* = 7.2 Hz, 1H), 1.38 (d, *J* = 7.0 Hz, 3H).

^{13}C NMR (100 MHz, $CDCl_3$): δ (ppm): 194.9, 166.1, 159.5, 143.3, 134.5, 132.1, 129.2, 129.0, 128.6, 128.5, 128.2, 128.2, 127.7, 127.2, 126.2, 49.8, 48.6, 47.5, 41.4, 38.1, 36.6, 21.8.

HR-MS (ESI): *m/z* calculated for $C_{28}H_{26}N_2O_3Na^+$: 461.1841, found: 461.1832.

(1*S*,2*R*,3*S*)-5-Benzyl-6,7-dioxo-*N*-((*S*)-1-phenylethyl)-2-(p-tolyl)-5-azaspiro[2.4]heptane-1-carboxamide 6b

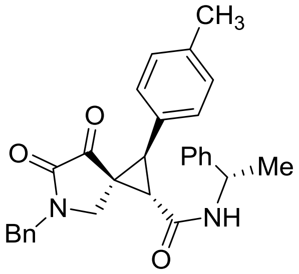

6b

Prepared according to the general procedure using 1-benzyl-4-(4-methylbenzylidene)pyrrolidine-2,3-dione **1c** (29.1 mg, 0.1 mmol, 1.0 equiv) and (*S*)-dimethyl(2-oxo-2-((1-phenylethyl)amino)ethyl)sulfonium bromide **4c** (30.4 mg, 0.1 mmol, 1.0 equiv). Purification of the crude product via flash chromatography on silica gel

(methanol/dichloromethane = 1:300) to afford the corresponding **6b** as a white solid with 70:30 dr, 97% yield, $[\alpha]_D^{20}$ = +96.6(*c* = 0.54 in $CHCl_3$).

Characterization data for the product **6b**:

^{1}H NMR (400 MHz, $CDCl_3$): δ (ppm): 7.35 – 7.30 (m, 3H), 7.28 – 7.19 (m, 7H), 7.07 (s, 4H), 7.04 – 7.01 (m, 1H), 5.07 – 4.97 (m, 1H), 4.79 (d, *J* = 14.4 Hz, 1H), 4.47 (d, *J* = 14.4 Hz, 1H), 3.64 (d, *J* = 12.2 Hz, 1H), 3.50 – 3.41 (m, 2H), 3.03 (d, *J* = 7.2 Hz, 1H), 2.31 (s, 3H), 1.46 (d, *J* = 7.0 Hz, 3H).

^{13}C NMR (100 MHz, $CDCl_3$): δ (ppm): 194.8, 166.2, 159.6, 143.3, 137.4, 134.6, 129.1, 129.0, 129.0, 128.9, 128.6, 128.5, 128.2, 127.2, 126.1, 49.7, 48.6, 47.5, 41.5, 38.3, 36.6, 21.9, 21.1.

HR-MS (ESI): *m/z* calculated for $C_{29}H_{28}N_2O_3Na^+$: 475.1998, found: 475.1995.

(1*S*,2*R*,3*S*)-5-Benzyl-2-(4-methoxyphenyl)-6,7-dioxo-*N*-((*S*)-1-phenylethyl)-5-azaspiro[2.4]heptane-1-carboxamide 6c

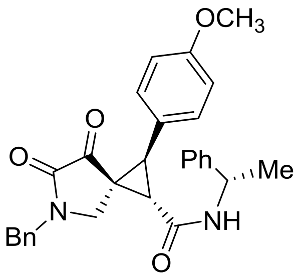

6c

Prepared according to the general procedure using 1-benzyl-4-(4-methoxybenzylidene)pyrrolidine-2,3-dione **1e** (30.7 mg, 0.1 mmol, 1.0 equiv) and (*S*)-dimethyl(2-oxo-2-((1-phenylethyl)amino)ethyl)sulfonium bromide **4c** (30.4 mg, 0.1 mmol, 1.0 equiv). Purification of the crude product via flash chromatography on silica gel (methanol/dichloromethane = 1:300) to afford the corresponding **6c** as a white solid with 64:36 dr, 91% yield, $[\alpha]_D^{20}$ = +94.6 (*c* = 0.84 in $CHCl_3$).

Characterization data for the product **6c**:

^{1}H NMR (400 MHz, $CDCl_3$): δ (ppm): 7.35 – 7.27 (m, 6H), 7.25 – 7.18 (m, 5H), 7.11 (d, *J* = 8.0 Hz, 2H), 6.82 – 6.75 (m, 2H), 5.06 – 4.97 (m, 1H), 4.79 (d, *J* = 14.4 Hz, 1H), 4.39 (d, *J* = 14.4 Hz, 1H), 3.76 (s, 3H), 3.62 (d, *J* = 12.2 Hz, 1H), 3.49 – 3.39 (m, 2H), 3.09 (d, *J* = 7.2 Hz, 1H), 1.44 (d, *J* = 7.0 Hz, 3H).

^{13}C NMR (100 MHz, CDCl3): δ (ppm): 194.9, 166.2, 159.6, 159.0, 143.4, 134.6, 130.3, 128.9, 128.5, 128.4, 128.2, 127.1, 126.2, 124.0, 113.5, 55.2, 49.7, 48.6, 47.5, 41.2, 38.4, 36.8, 21.8.

HR-MS (ESI): m/z calculated for $C_{29}H_{28}N_2O_4Na+$: 491.1947, found: 491.1947.

(1*S*,2*R*,3*S*)-5-Benzyl-2-(4-fluorophenyl)-6,7-dioxo-*N*-((*S*)-1-phenylethyl)-5-azaspiro[2.4]heptane-1-carboxamide 6d

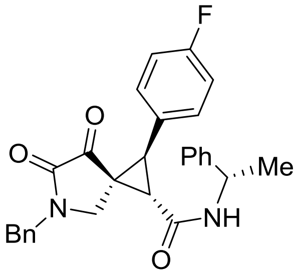

6d

Prepared according to the general procedure using 1-benzyl-4-(4-fluorophenyl)pyrrolidine-2,3-dione **1g** (29.5 mg, 0.1 mmol, 1.0 equiv) and (*S*)-dimethyl(2-oxo-2-((1-phenylethyl)amino)ethyl)sulfonium bromide **4c** (30.4 mg, 0.1 mmol, 1.0 equiv). Purification of the crude product via flash chromatography on silica gel (methanol/dichloromethane = 1:300) to afford the corresponding **6d** as a white solid with 81:19 dr, 92% yield, $[\alpha]_D^{20}$ = +112.0 (c = 0.97 in $CHCl_3$).

Characterization data for the product **6d**:

^{1}H NMR (400 MHz, $CDCl_3$): δ (ppm): 7.36 – 7.27 (m, 6H), 7.26 – 7.20 (m, 5H), 7.17 – 7.12 (m, 2H), 6.96 – 6.89 (m, 2H), 5.07 – 4.98 (m, 1H), 4.79 (d, J = 14.4 Hz, 1H), 4.41 (d, J = 14.4 Hz, 1H), 3.63 (d, J = 12.2 Hz, 1H), 3.47 – 3.39 (m, 2H), 3.06 (d, J = 7.2 Hz, 1H), 1.47 (d, J = 7.0 Hz, 3H).

^{13}C NMR (100 MHz, CDCl3): δ (ppm): 195.0, 165.9, 159.5, 143.3, 134.4, 130.9, 130.8, 129.0, 128.5, 128.5, 128.3, 127.2, 126.2, 115.2, 115.0, 49.8, 48.7, 47.4, 40.5, 38.0, 36.8, 21.8.

HR-MS (ESI): *m/z* calculated for $C_{28}H_{25}FN_2O_3Na^+$: 479.1747 , found: 479.1749.

(1*S*,2*S*,3*S*)-5-Benzyl-2-(2-chlorophenyl)-6,7-dioxo-*N*-((*S*)-1-phenylethyl)-5-azaspiro[2.4]heptane-1-carboxamide 6e

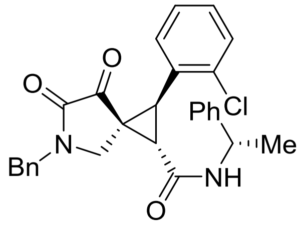

6e

Prepared according to the general procedure using 1-benzyl-4-(2-chlorophenyl) pyrrolidine-2,3-dione **1h** (31.1 mg, 0.1 mmol, 1.0 equiv) and (*S*)-dimethyl(2-oxo-2-((1-phenylethyl)amino)ethyl)sulfonium bromide **4c** (30.4 mg, 0.1 mmol, 1.0 equiv). Purification of the crude product via flash chromatography on silica gel (methanol/dichloromethane = 1:300) to afford the corresponding **6e** as a white solid with 70:30 dr, 90% yield, $[\alpha]_D^{20}$ = +104.3 (c = 1.01 in $CHCl_3$).

Characterization data for the product **6e**:

^{1}H NMR (400 MHz, $CDCl_3$): δ (ppm): 7.38 – 7.28 (m, 7H), 7.27 – 7.20 (m, 7H), 7.19 – 7.13 (m, 1H), 5.09 – 4.93 (m, 2H), 4.22 (d, J = 14.4 Hz, 1H), 3.71 (d, J = 12.2 Hz, 1H), 3.44 (d, J = 12.2 Hz, 1H), 3.28 (d, J = 7.2 Hz, 1H), 2.96 (d, J = 7.2 Hz, 1H), 1.48 (d, J = 7.0 Hz, 3H).

^{13}C NMR (100 MHz, CDCl3): δ (ppm): 194.5, 165.7, 159.4, 143.4, 135.3, 134.5, 131.2, 130.9, 129.1, 129.1, 128.9, 128.6, 128.5, 128.2, 127.2, 126.5, 126.2, 49.8, 48.5, 48.5, 46.7, 39.0, 37.0, 36.5, 21.8.

HR-MS (ESI): *m/z* calculated for $C_{28}H_{25}ClN_2O_3Na^+$: 495.1451, found: 495.1449.

(1*S*,2*R*,3*S*)-5-benzyl-2-(4-bromophenyl)-6,7-dioxo-*N*-((*S*)-1-phenylethyl)-5-azaspiro[2.4]heptane-1-carboxamide 6f

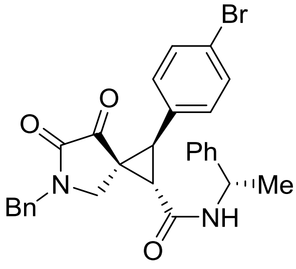

6f

Prepared according to the general procedure using 1-benzyl-4-(4-bromophenyl) pyrrolidine-2,3-dione **1l** (35.6 mg, 0.1 mmol, 1.0 equiv) and (*S*)-dimethyl(2-oxo-2-((1-phenylethyl)amino)ethyl)sulfonium bromide **4c** (30.4 mg, 0.1 mmol, 1.0 equiv). Purification of the crude product via flash chromatography on silica gel (methanol/dichloromethane = 1:300) to afford the corresponding **6f** as a white solid with 62:38 dr, 81% yield, $[\alpha]_D^{20}$ = +106.8 (*c* = 0.44 in $CHCl_3$).

Characterization data for the product **6f**:

^{1}H NMR (400 MHz, $CDCl_3$): δ (ppm): 7.40 – 7.32 (m, 5H), 7.31 – 7.27 (m, 2H), 7.27 – 7.21 (m, 5H), 7.06 – 6.98 (m, 3H), 5.09 – 4.99 (m, 1H), 4.79 (d, *J* = 14.4 Hz, 1H), 4.42 (d, *J* = 14.4 Hz, 1H), 3.64 (d, *J* = 12.2 Hz, 1H), 3.46 (d, *J* = 12.2 Hz, 1H), 3.40 (d, *J* = 7.2 Hz, 1H), 2.99 (d, *J* = 7.2 Hz, 1H), 1.49 (d, *J* = 7.0 Hz, 3H).

^{13}C NMR (100 MHz, CDCl3): δ (ppm): 194.8, 165.7, 159.3, 143.1, 134.4, 131.3, 131.1, 130.9, 129.0, 128.6, 128.5, 128.3, 127.3, 126.2, 121.7, 49.8, 48.7, 47.4, 40.5, 37.9, 36.6, 21.7.

HR-MS (ESI): *m/z* calculated for $C_{28}H_{25}BrN_2O_3Na^+$: 539.0946, found: 539.0935.

(1*S*,2*R*,3*S*)-5-Benzyl-2-(naphthalen-1-yl)-6,7-dioxo-*N*-((*S*)-1-phenylethyl)-5-azaspiro[2.4]heptane-1-carboxamide 6g

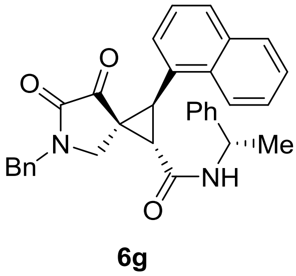

6g

Prepared according to the general procedure using 1-benzyl-4-(naphthalen-1-ylmethylene)pyrrolidine-2,3-dione **1o** (32.7 mg, 0.1 mmol, 1.0 equiv) and (*S*)-dimethyl(2-oxo-2-((1-phenylethyl)amino)ethyl)sulfonium bromide **4c** (30.4 mg, 0.1 mmol, 1.0 equiv). Purification of the crude product via flash chromatography on silica gel (methanol/dichloromethane = 1:300) to afford the corresponding **6g** as a white solid with 77:28 dr, 97% yield, $[\alpha]_D^{20}$ = +116.9 (*c* = 0.12 in $CHCl_3$).

Characterization data for the product **6g**:

^{1}H NMR (400 MHz, $CDCl_3$): δ (ppm): 7.78 – 7.70 (m, 2H), 7.41 – 7.31 (m, 6H), 7.29 – 7.23 (m, 4H), 7.22 – 7.15 (m, 5H), 6.85 (d, *J* = 7.8 Hz, 1H), 5.07 (d, *J* = 14.4 Hz, 1H), 4.99 – 4.90 (m, 1H), 4.09 (d, *J* = 14.4 Hz, 1H), 3.82 (d, *J* = 12.2 Hz, 1H), 3.63 (d, *J* = 7.2 Hz, 1H), 3.51 (d, *J* = 12.2 Hz, 1H), 3.08 (d, *J* = 7.2 Hz, 1H), 1.32 (d, *J* = 7.0 Hz, 3H).

^{13}C NMR (100 MHz, CDCl3): δ (ppm): 194.0, 166.1, 159.3, 143.3, 134.8, 133.5, 132.6, 129.0, 129.0, 128.7, 128.6, 128.5, 128.4, 128.4, 127.3, 127.1, 126.8, 126.2, 125.9, 124.9, 122.4, 49.9, 48.5, 47.1, 39.4, 37.6, 36.0, 21.8.

HR-MS (ESI): *m/z* calculated for $C_{32}H_{28}N_2O_3Na^+$: 511.1998, found: 511.2004.

(1*S*,2*R*,3*S*)-5-Benzyl-2-(naphthalen-2-yl)-6,7-dioxo-*N*-((*S*)-1-phenylethyl)-5-azaspiro[2.4]heptane-1-carboxamide 6h

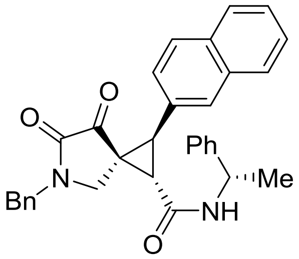

6h

Prepared according to the general procedure using 1-benzyl-4-(naphthalen-2-ylmethylene)pyrrolidine-2,3-dione **1r** (32.7 mg, 0.1 mmol, 1.0 equiv) and (*S*)-dimethyl(2-oxo-2-((1-phenylethyl)amino)ethyl)sulfonium bromide **4c** (30.4 mg, 0.1 mmol, 1.0 equiv). Purification of the crude product via flash chromatography on silica gel (methanol/dichloromethane = 1:300) to afford the corresponding **6h** as a white solid with 77:23 dr, 99% yield, $[\alpha]_D^{20}$ = +120.0 (*c* = 0.40 in $CHCl_3$).

Characterization data for the product **6h**:

^{1}H NMR (400 MHz, $CDCl_3$): δ (ppm): 7.83 – 7.76 (m, 2H), 7.73 – 7.66 (m, 2H), 7.51 – 7.45 (m, 2H), 7.34 (q, *J* = 3.8 Hz, 3H), 7.30 – 7.22 (m, 8H), 6.53 (d, *J* = 7.6 Hz, 1H), 5.11 – 5.00 (m, 1H), 4.79 (d, *J* = 14.4 Hz, 1H), 4.53 (d, *J* = 14.4 Hz, 1H), 3.74 (d, *J* = 12.2 Hz, 1H), 3.61 (d, *J* = 7.4 Hz, 1H), 3.56 (d, *J* = 12.2 Hz, 1H), 3.07 (d, *J* = 7.4 Hz, 1H), 1.47 (d, *J* = 7.0 Hz, 3H).

^{13}C NMR (100 MHz, DMSO-d^6): δ (ppm): 194.5, 166.1, 159.4, 144.1, 135.5, 132.5, 132.2, 130.8, 128.8, 128.3, 128.0, 127.8, 127.7, 127.5, 127.5, 127.4, 126.7, 126.3, 126.0, 125.8, 48.4, 47.5, 47.4, 40.6, 38.1, 34.7, 22.5.

HR-MS (ESI): *m/z* calculated for $C_{32}H_{28}N_2O_3Na^+$: 511.1998, found: 511.1997.

(1*S*,2*S*,3*R*)-5-Benzyl-6,7-dioxo-*N*-((*S*)-1-phenylethyl)-2-(thiophen-2-yl)-5-azaspiro[2.4]heptane-1-carboxamide 6i

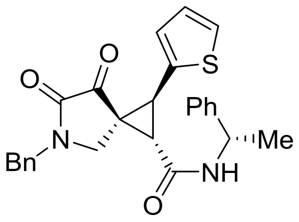

6i

Prepared according to the general procedure using 1-benzyl-4-(thiophen-2-ylmethylene)pyrrolidine-2,3-dione **1p** (28.3 mg, 0.1 mmol, 1.0 equiv) and (*S*)-dimethyl(2-oxo-2-((1-phenylethyl)amino)ethyl)sulfonium bromide **4c** (30.4 mg, 0.1 mmol, 1.0 equiv). Purification of the crude product via flash chromatography on silica gel (methanol/dichloromethane = 1:300) to afford the corresponding **6i** as a white solid with 60:40 dr, 90% yield, $[\alpha]_D^{20}$ = +97.6 (c = 0.54 in $CHCl_3$).

Characterization data for the product **6i**:

^{1}H NMR (400 MHz, $CDCl_3$): δ (ppm): 7.38 – 7.32 (m, 3H), 7.31 – 7.22 (m, 7H), 7.20 – 7.17 (m, 1H), 7.00 – 6.96 (m, 1H), 6.95 – 6.90 (m, 1H), 6.83 (d, J = 7.6 Hz, 1H), 5.11 – 4.97 (m, 1H), 4.76 (d, J = 14.4 Hz, 1H), 4.52 (d, J = 14.4 Hz, 1H), 3.65 (d, J = 12.2 Hz, 1H), 3.56 – 3.43 (m, 2H), 3.04 (d, J = 7.0 Hz, 1H), 1.49 (d, J = 7.0 Hz, 3H).

^{13}C NMR (100 MHz, $CDCl_3$): δ (ppm): 193.8, 165.5, 159.4, 143.0, 134.8, 134.5, 129.0, 128.7, 128.5, 128.3, 127.6, 127.4, 126.8, 126.1, 125.5, 49.9, 48.7, 47.2, 38.7, 37.7, 36.1, 21.8.

HR-MS (ESI): m/z calculated for $C_{26}H_{24}N_2O_3SNa^+$: 467.1405, found: 467.1406.

5. Crystal data and structure refinement for the representative product 3n

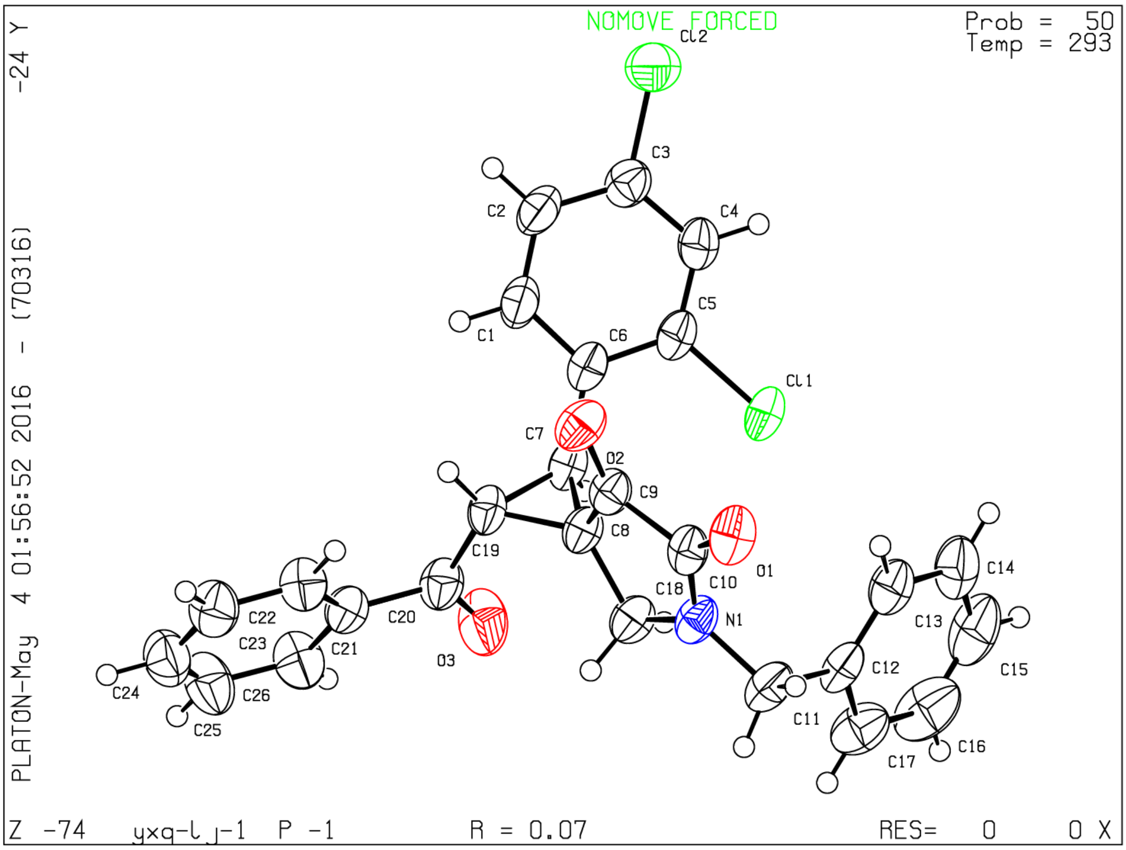

Identification code	3n
Empirical formula	$C_{26}H_{19}Cl_2NO_3$
Formula weight	464.32
Temperature/K	292.7(8)
Crystal system	triclinic
Space group	P-1
a/Å	9.5083(6)
b/Å	10.9485(6)
c/Å	11.7032(6)
α/°	76.171(5)
β/°	72.435(5)
γ/°	80.124(5)
Volume/Å^3	1121.35(12)
Z	2
ρ_{calc}g/cm^3	1.375
μ/mm^{-1}	2.839
F(000)	480.0
Crystal size/mm^3	0.8 × 0.7 × 0.2
Radiation	CuKα (λ = 1.54184)
2Θ range for data collection/ °	9.814 to 134.142
Index ranges	-11 ≤ h ≤ 11, -11 ≤ k ≤ 13, -13 ≤ l ≤ 13
Reflections collected	19516
Independent reflections	3998 [R_{int} = 0.0811, R_{sigma} = 0.0420]
Data/restraints/parameters	3998/0/289
Goodness-of-fit on F^2	1.054
Final R indexes [I>=2σ (I)]	R_1 = 0.0685, wR_2 = 0.1862

Final R indexes [all data]	$R_1 = 0.0716$, $wR_2 = 0.1930$
Largest diff. peak/hole / e Å^{-3}	0.30/-0.63

6. Crystal data and structure refinement for the representative product 6e

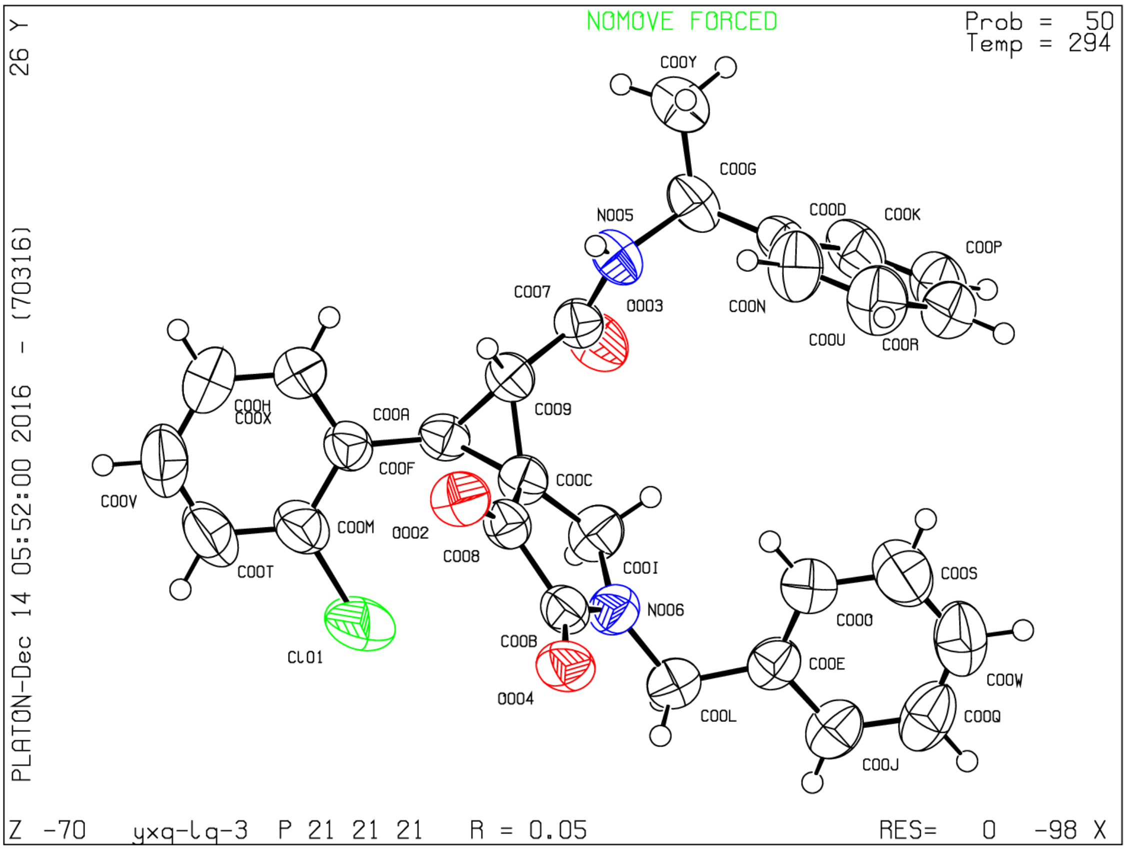

Identification code	6e
Empirical formula	$C_{28}H_{25}ClN_2O_3$
Formula weight	472.95
Temperature/K	294.08(10)
Crystal system	orthorhombic
Space group	$P2_12_12_1$
a/Å	10.14653(19)
b/Å	14.7197(4)
c/Å	17.0048(6)

α/°	90
β/°	90
γ/°	90
Volume/Å^3	2539.74(12)
Z	4
ρ_{calc}g/cm^3	1.237
μ/mm^{-1}	1.580
F(000)	992.0
Crystal size/mm^3	0.6 × 0.4 × 0.3
Radiation	CuKα (λ = 1.54184)
2Θ range for data collection/°	10.152 to 134.1
Index ranges	$-8 \leq h \leq 12$, $-17 \leq k \leq 17$, $-18 \leq l \leq 20$
Reflections collected	16542
Independent reflections	4344 [R_{int} = 0.0313, R_{sigma} = 0.0211]
Data/restraints/parameters	4344/0/308
Goodness-of-fit on F^2	1.031
Final R indexes [I>=2σ (I)]	R_1 = 0.0471, wR_2 = 0.1291
Final R indexes [all data]	R_1 = 0.0499, wR_2 = 0.1331
Largest diff. peak/hole / e Å^{-3}	0.25/-0.26
Flack parameter	0.008(7)

7. References and notes

1. Krell, E. *Handbook of Laboratory Distillation*; Elseriver Publishing Company, Amsterdam London-New York, 1963.

2. Rosengart, M. J. *The Technique of Distillation and Rectification in the Laboratory*; VEB Verlag Technik: Berlin, Germany, 1954.

3. Stage, H. *Columns for laboratory distillation*, *Angew. Chem.*, **1947**, *B19*, 175.

4. Southwick, P. L.; Barnas, E. F. *J. Org. Chem.* **1962**, *27*, 98-106. [CrossRef]

8. NMR Spectra of the multi-substituted spirocyclopropanes

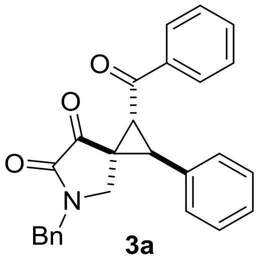

3a

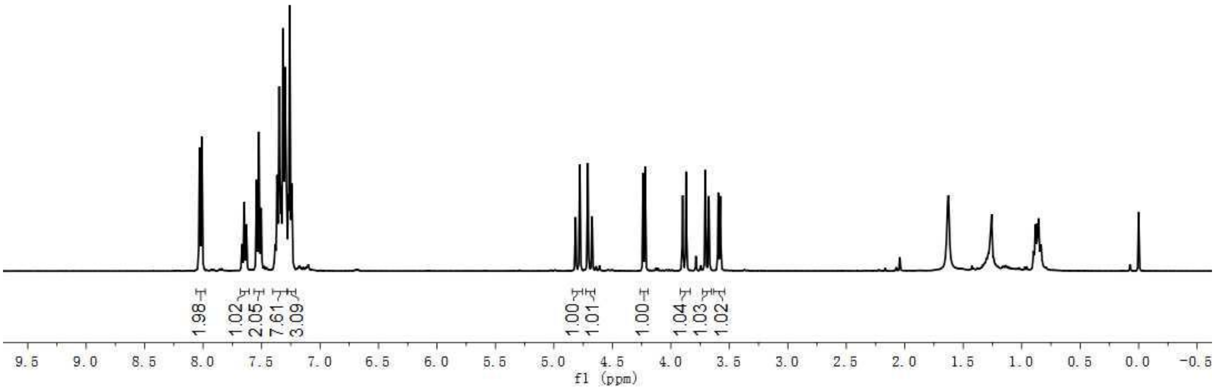

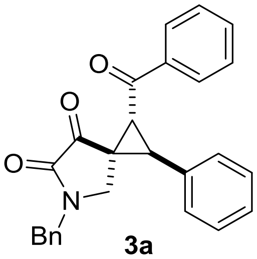

3a

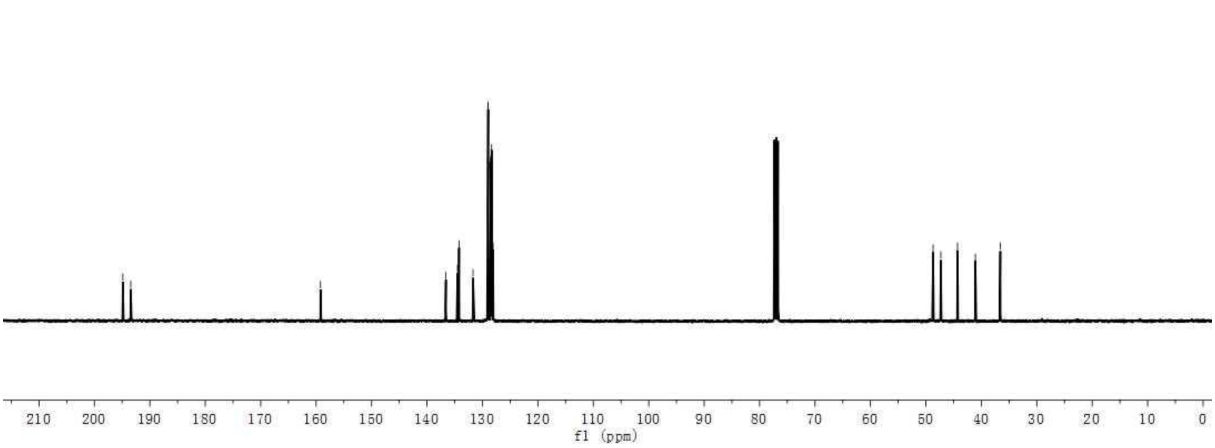

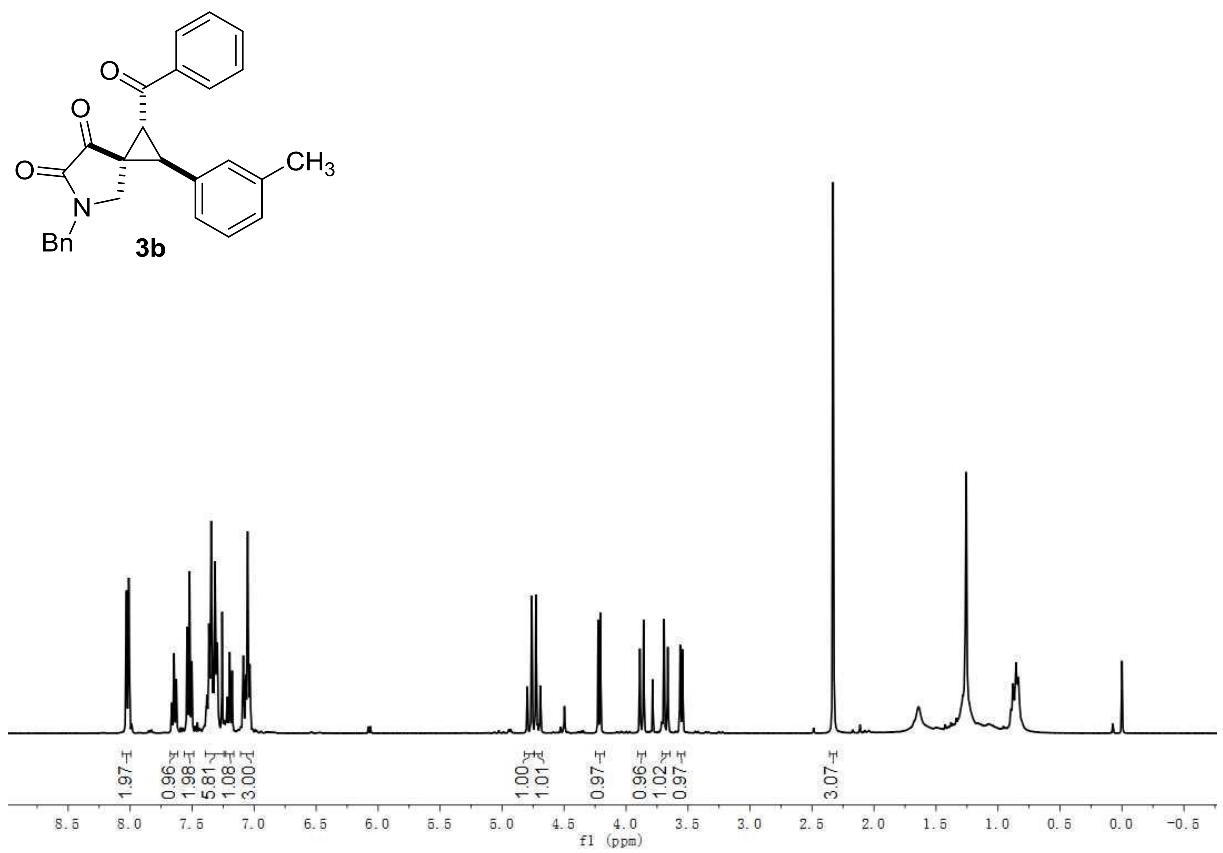
O
O
O
N
Bn
CH3
3b
1.97
0.96
1.98
5.81
1.08
3.00
1.00
1.01
0.97
0.96
1.02
0.97
3.07
8.5 8.0 7.5 7.0 6.5 6.0 5.5 5.0 4.5 4.0 3.5 3.0 2.5 2.0 1.5 1.0 0.5 0.0 -0.5
f1 (ppm)

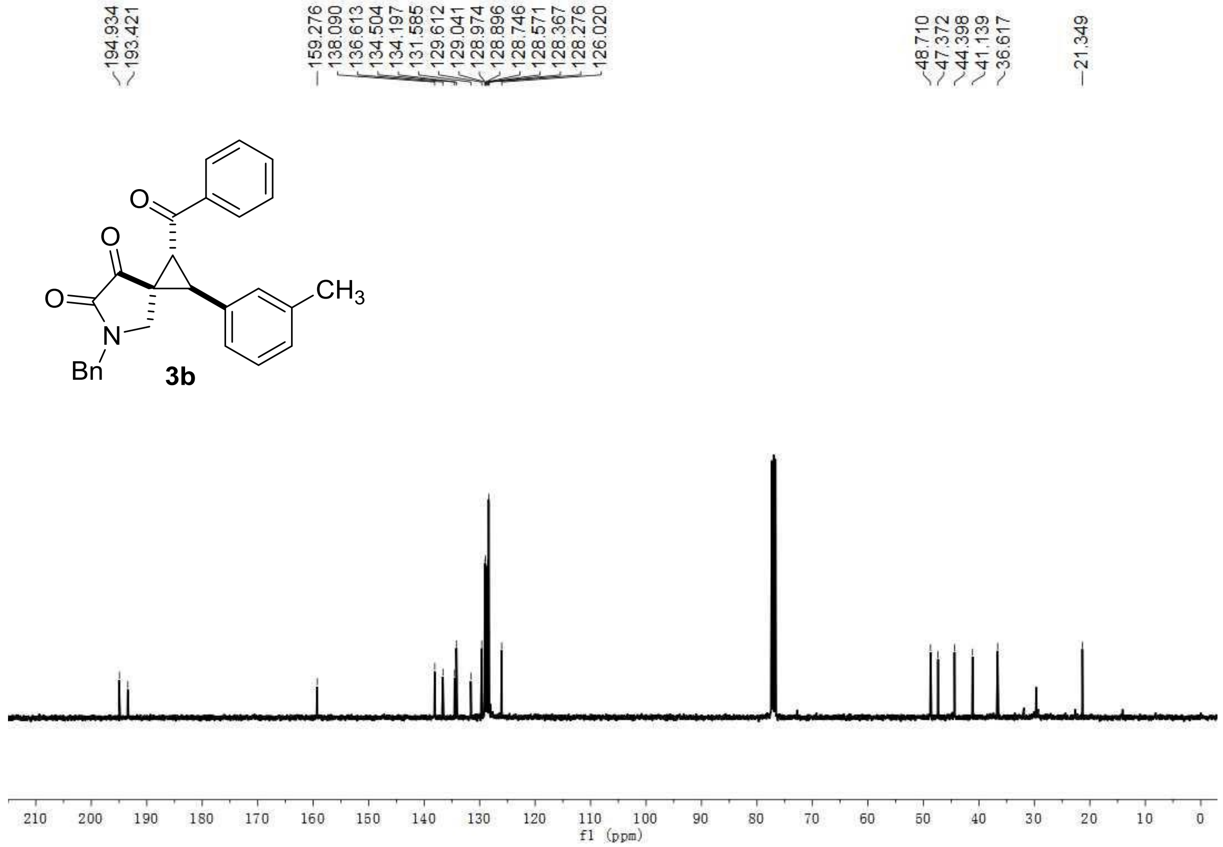
194.934
193.421
159.276
138.090
136.613
134.504
134.197
131.585
129.612
129.041
128.974
128.896
128.746
128.571
128.367
128.276
126.020
48.710
47.372
44.398
41.139
36.617
21.349
O
O
O
N
Bn
CH3
3b
210 200 190 180 170 160 150 140 130 120 110 100 90 80 70 60 50 40 30 20 10 0
f1 (ppm)

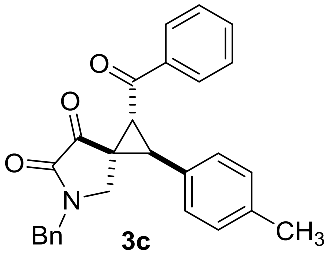
O
O
O
N
Bn
CH3

3c

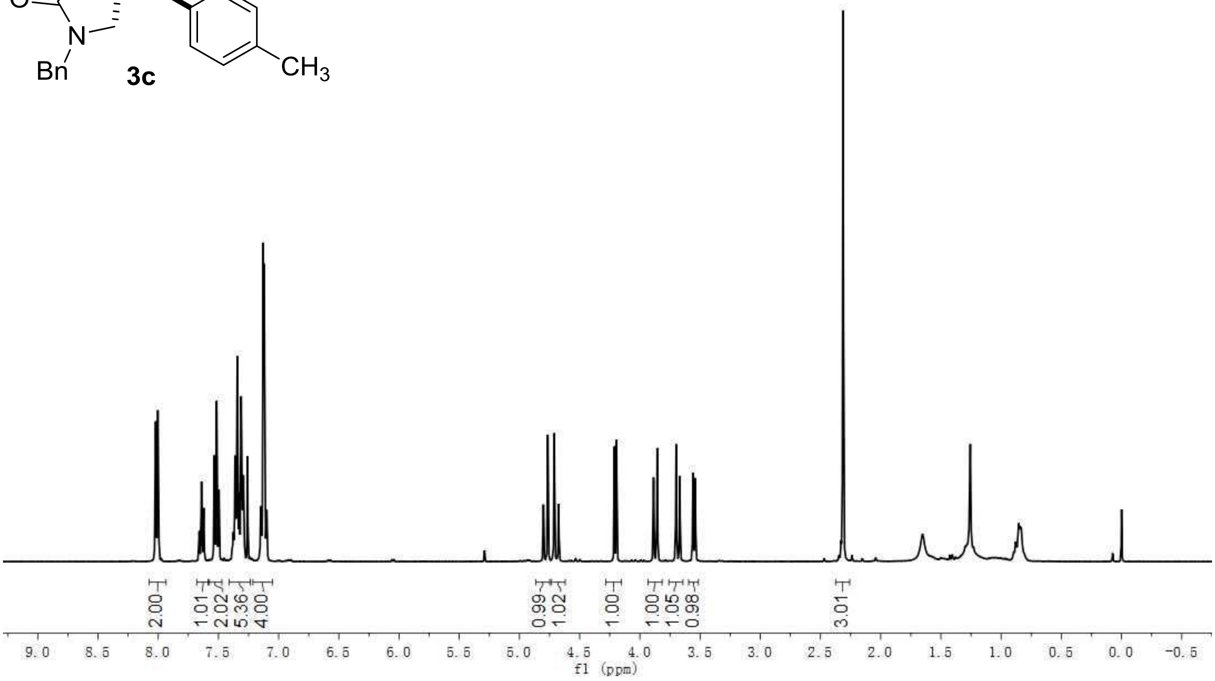
2.00
1.01
2.02
5.36
4.00
0.99
1.02
1.00
1.00
1.05
0.98
3.01
9.0 8.5 8.0 7.5 7.0 6.5 6.0 5.5 5.0 4.5 4.0 3.5 3.0 2.5 2.0 1.5 1.0 0.5 0.0 -0.5
f1 (ppm)

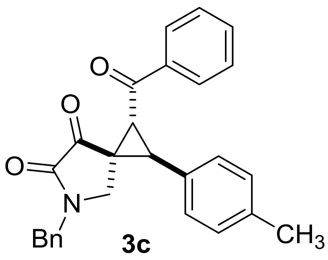
O
O
O
N
Bn
CH3

3c

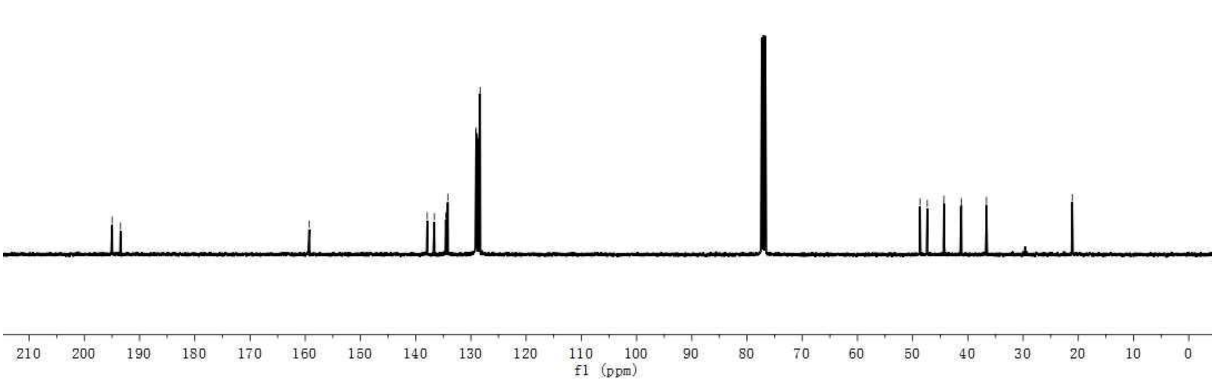
194.976
193.449
159.261
137.900
136.628
134.536
134.178
129.108
129.038
128.966
128.816
128.662
128.615
128.551
128.349
48.687
47.348
44.316
41.197
36.650
21.100
210 200 190 180 170 160 150 140 130 120 110 100 90 80 70 60 50 40 30 20 10 0
f1 (ppm)

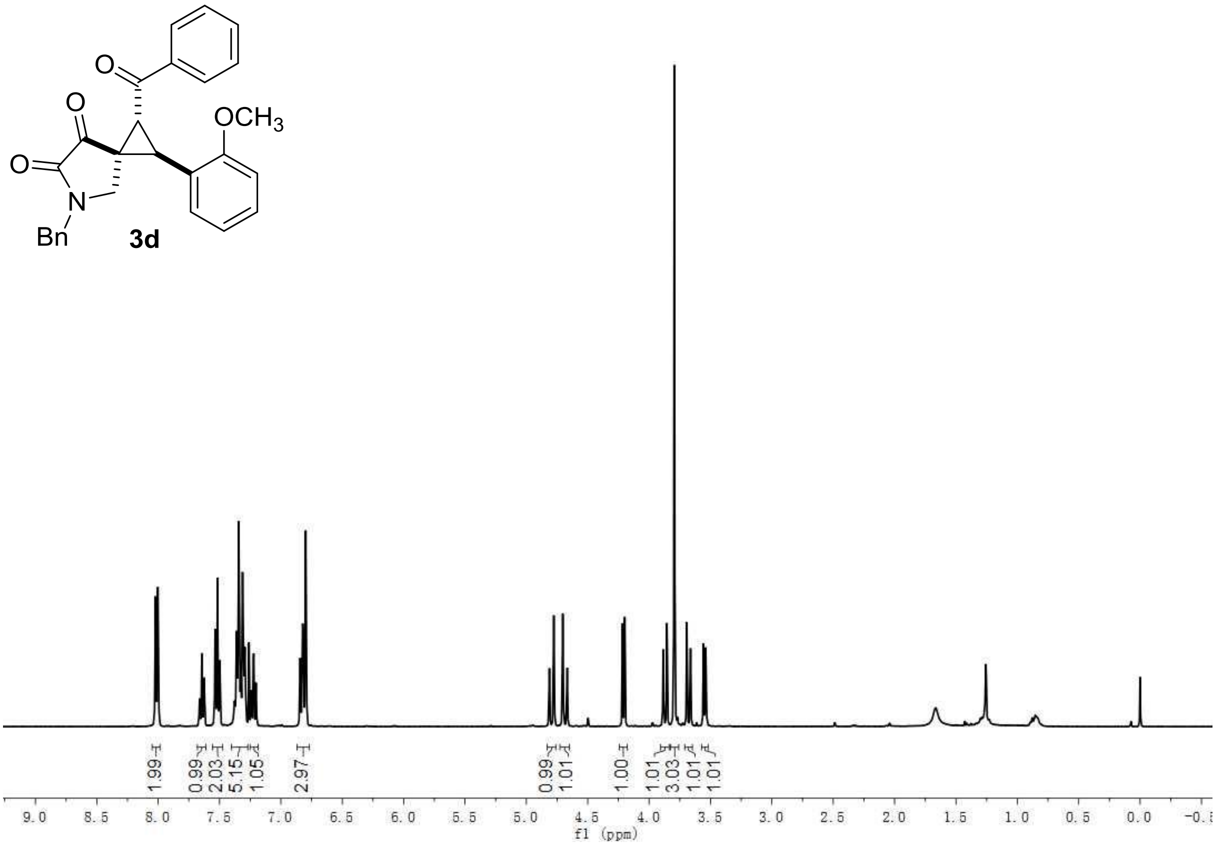
OCH3
O
O
O
N
Bn
1.99
0.99
2.03
5.15
1.05
2.97
0.99
1.01
1.00
1.01
3.03
1.01
1.01
9.0 8.5 8.0 7.5 7.0 6.5 6.0 5.5 5.0 4.5 4.0 3.5 3.0 2.5 2.0 1.5 1.0 0.5 0.0
f1 (ppm)

3d

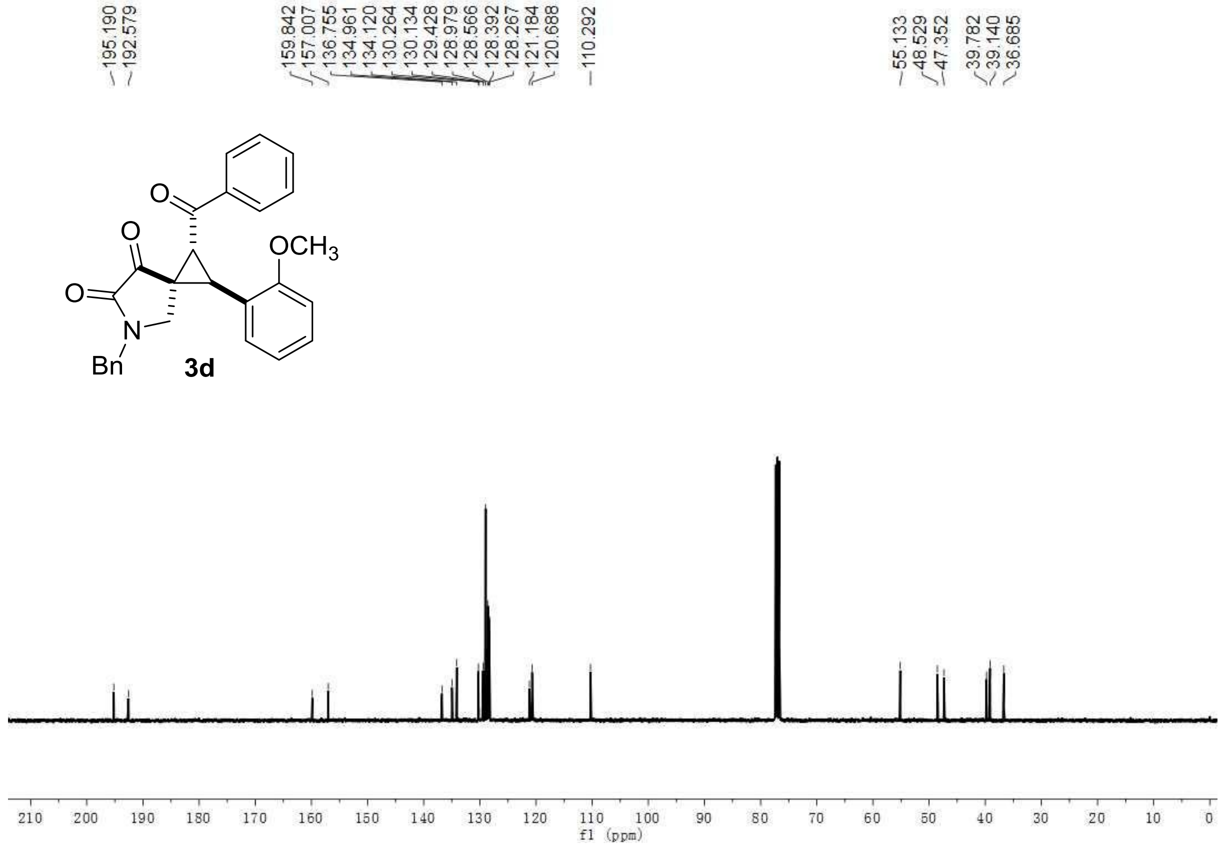
195.190
192.579
159.842
157.007
136.755
134.961
134.120
130.264
130.134
129.428
128.979
128.566
128.392
128.267
121.184
120.688
110.292
55.133
48.529
47.352
39.782
39.140
36.685
OCH3
O
O
O
N
Bn
210 200 190 180 170 160 150 140 130 120 110 100 90 80 70 60 50 40 30 20 10 0
f1 (ppm)

3d

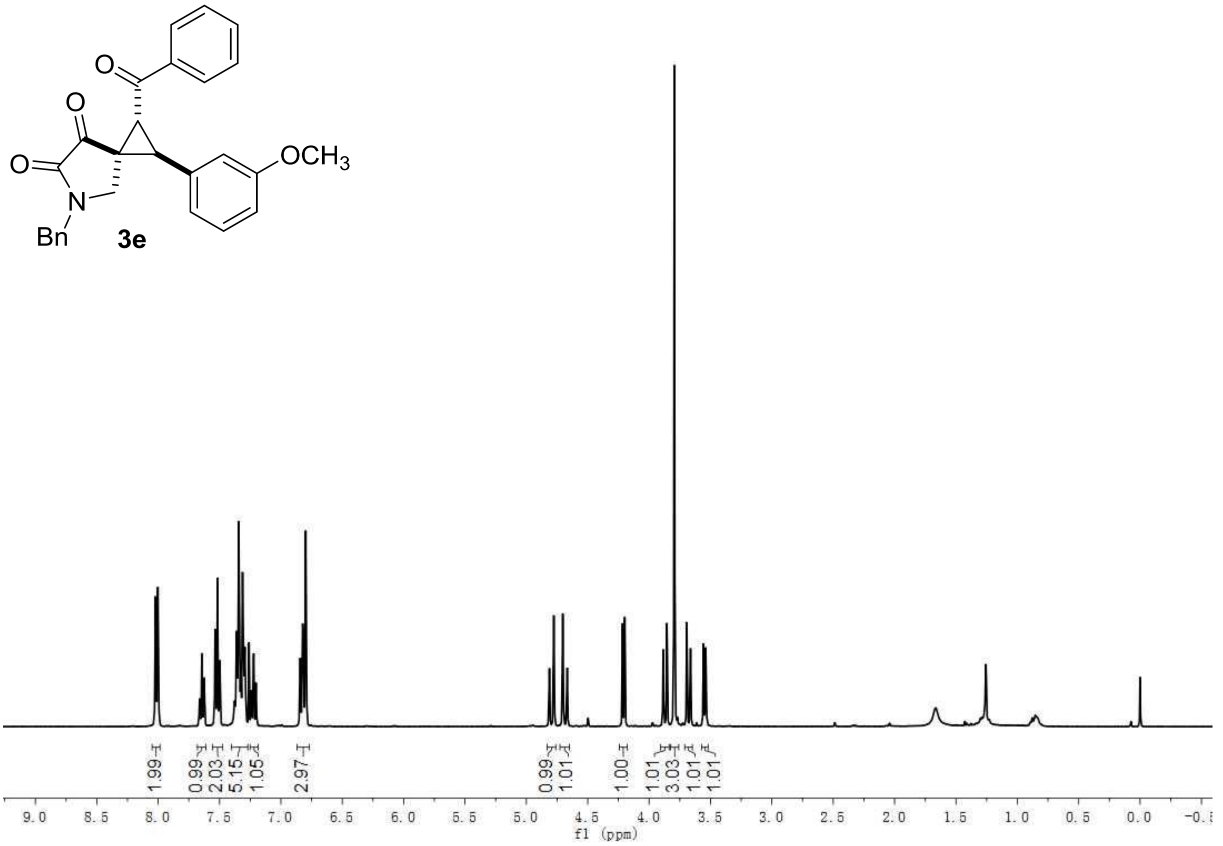
3e
OCH3
Bn
1.99
0.99
2.03
5.15
1.05
2.97
0.99
1.01
1.00
1.01
3.03
1.01
1.01
f1 (ppm)

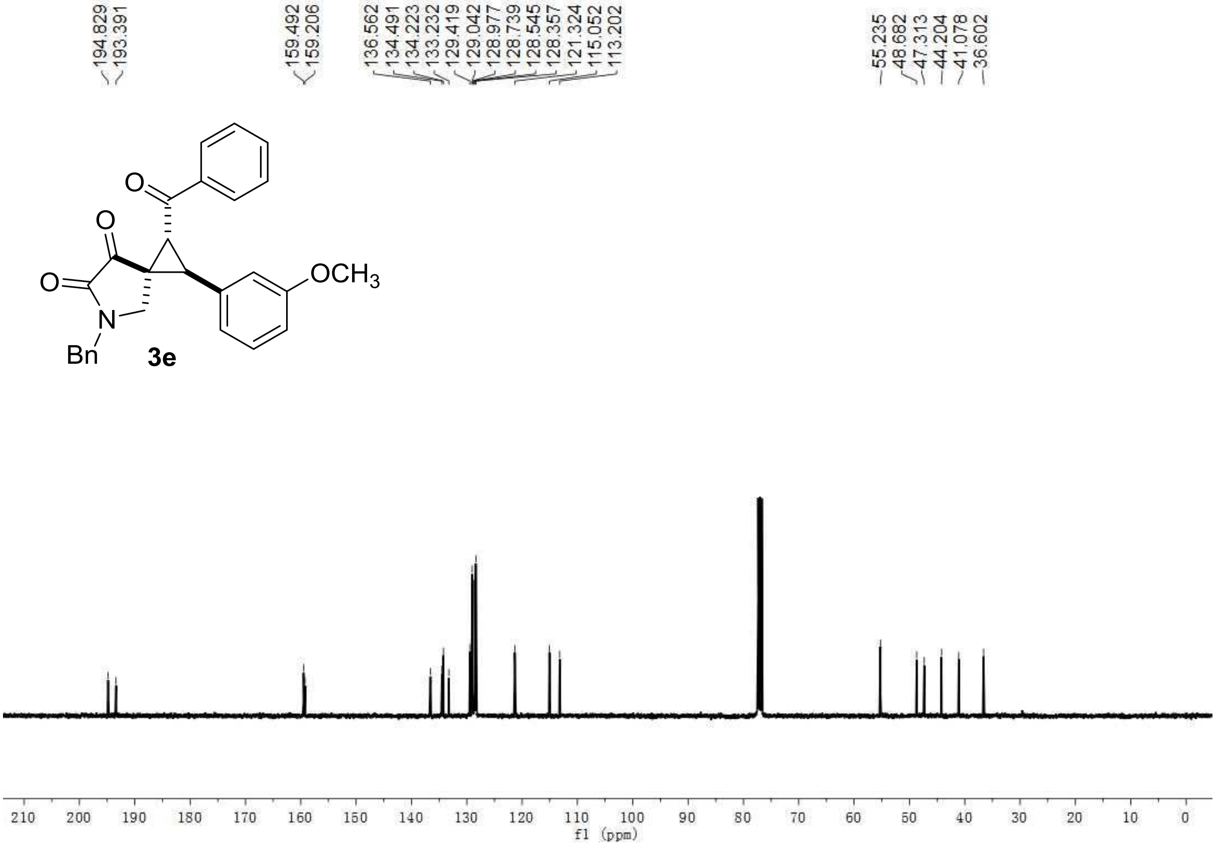
194.829
193.391
159.492
159.206
136.562
134.491
134.223
133.232
129.419
129.042
128.977
128.739
128.545
128.357
121.324
115.052
113.202
55.235
48.682
47.313
44.204
41.078
36.602
3e
OCH3
Bn
f1 (ppm)

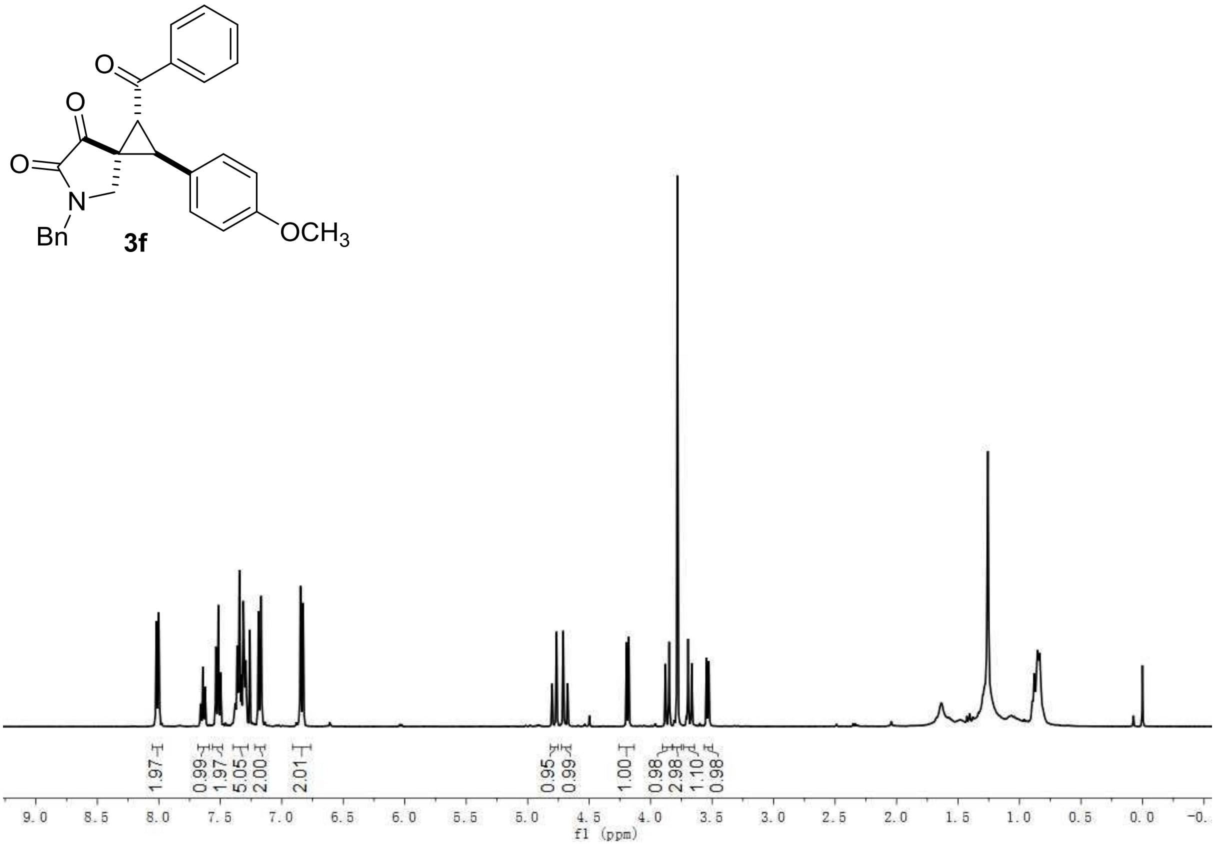
O
O
O
N
Bn
3f
OCH3
1.97
0.99
1.97
5.05
2.00
2.01
0.95
0.99
1.00
0.98
2.98
1.10
0.98
9.0 8.5 8.0 7.5 7.0 6.5 6.0 5.5 5.0 4.5 4.0 3.5 3.0 2.5 2.0 1.5 1.0 0.5 0.0
f1 (ppm)

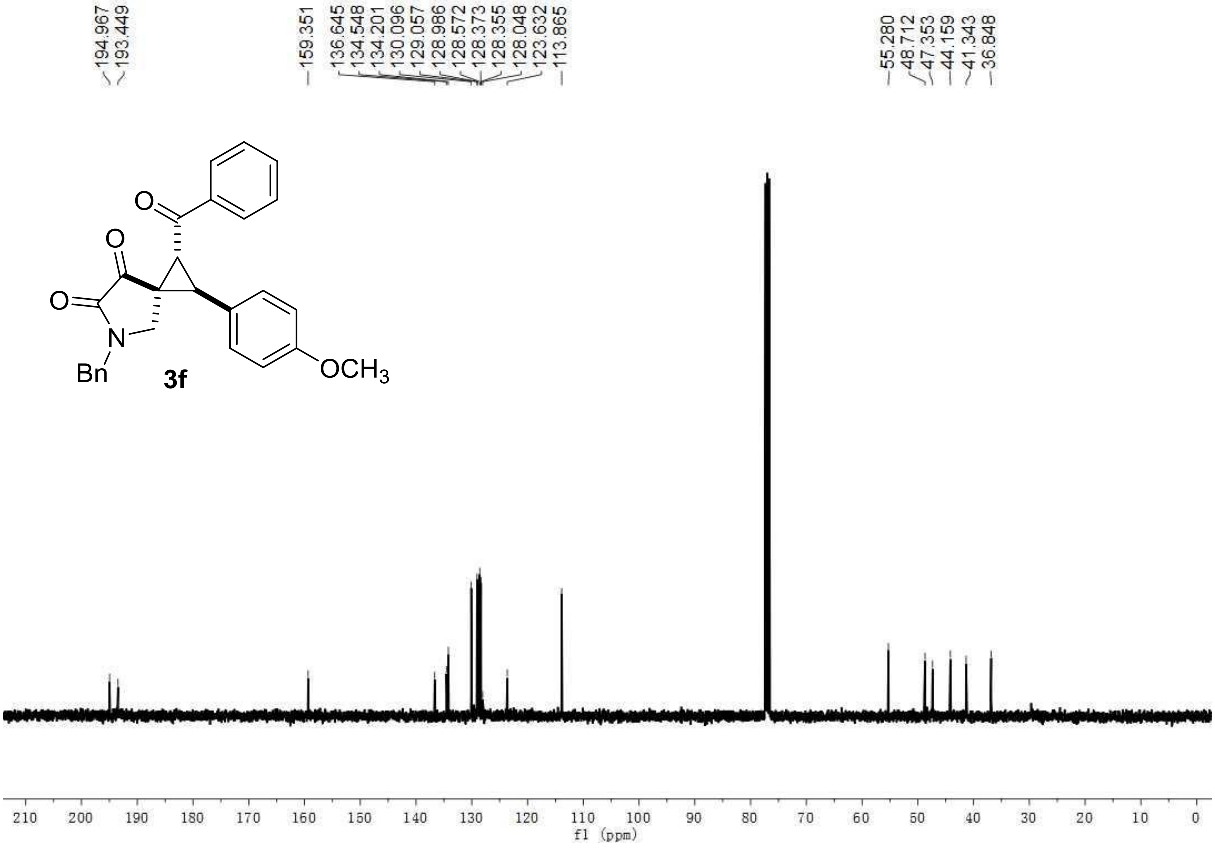
194.967
193.449
159.351
136.645
134.548
134.201
130.096
129.057
128.986
128.572
128.373
128.355
128.048
123.632
113.865
55.280
48.712
47.353
44.159
41.343
36.848
O
O
O
N
Bn
3f
OCH3
210 200 190 180 170 160 150 140 130 120 110 100 90 80 70 60 50 40 30 20 10 0
f1 (ppm)

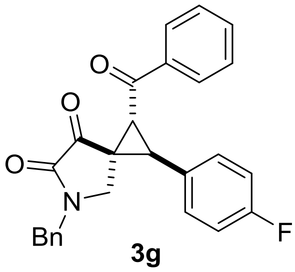
O
O
O
N
Bn
F

3g

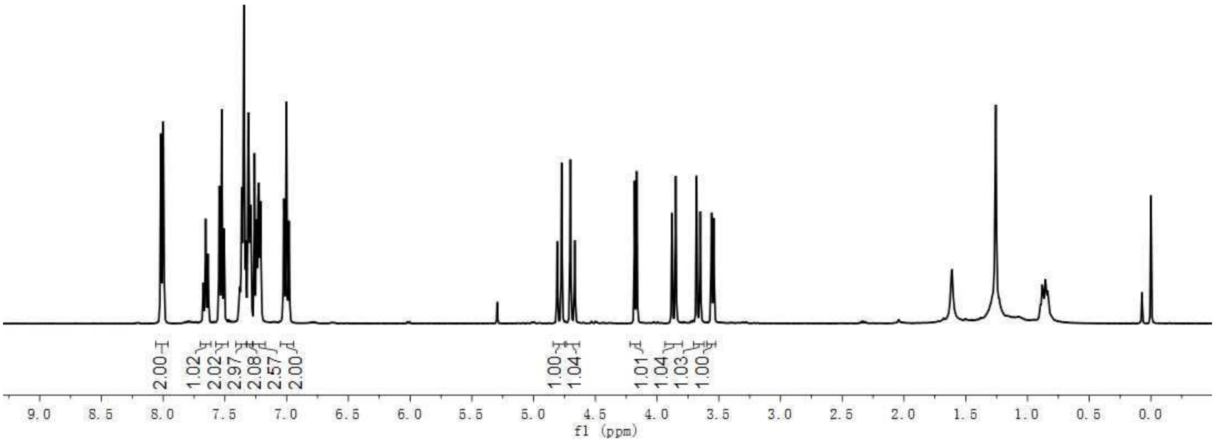
2.00
1.02
2.02
2.97
2.08
2.57
2.00
1.00
1.04
1.01
1.04
1.03
1.00
9.0 8.5 8.0 7.5 7.0 6.5 6.0 5.5 5.0 4.5 4.0 3.5 3.0 2.5 2.0 1.5 1.0 0.5 0.0
f1 (ppm)

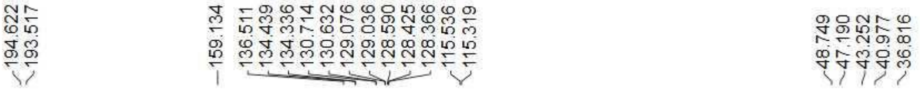
194.622
193.517
159.134
136.511
134.439
134.336
130.714
130.632
129.076
129.036
128.590
128.425
128.366
115.536
115.319
48.749
47.190
43.252
40.977
36.816

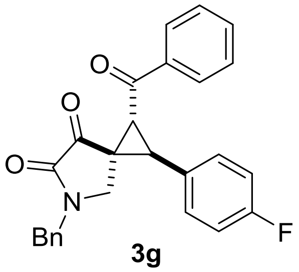
O
O
O
N
Bn
F

3g

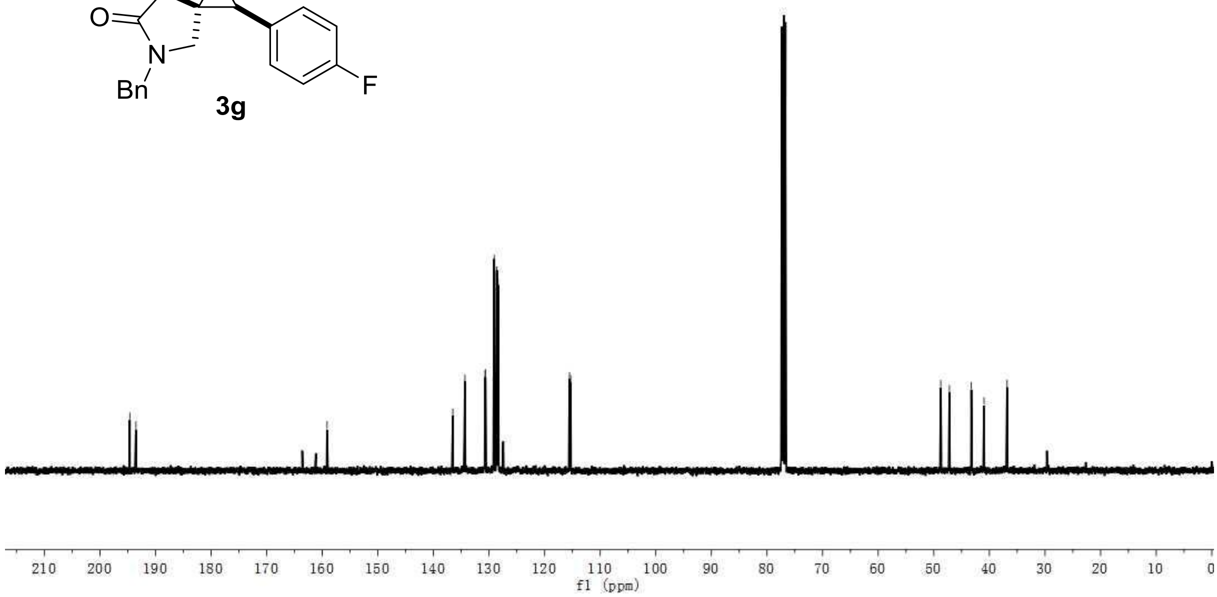
210 200 190 180 170 160 150 140 130 120 110 100 90 80 70 60 50 40 30 20 10 0
f1 (ppm)

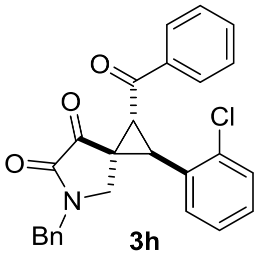
O
O
O
Cl
N
Bn

3h

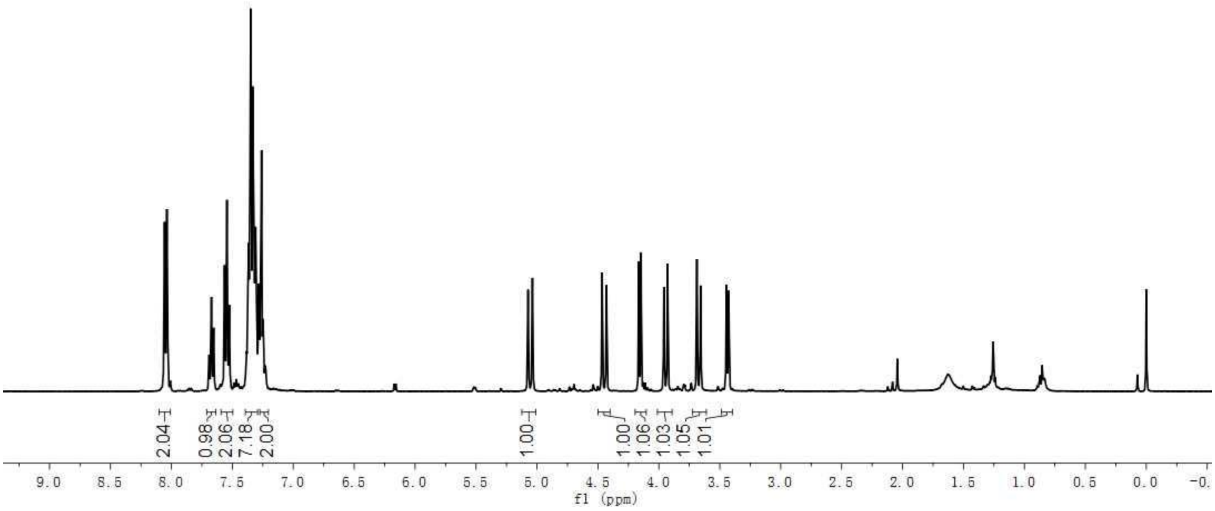
2.04
0.98
2.06
7.18
2.00
1.00
1.00
1.06
1.03
1.05
1.01
9.0 8.5 8.0 7.5 7.0 6.5 6.0 5.5 5.0 4.5 4.0 3.5 3.0 2.5 2.0 1.5 1.0 0.5 0.0
f1 (ppm)

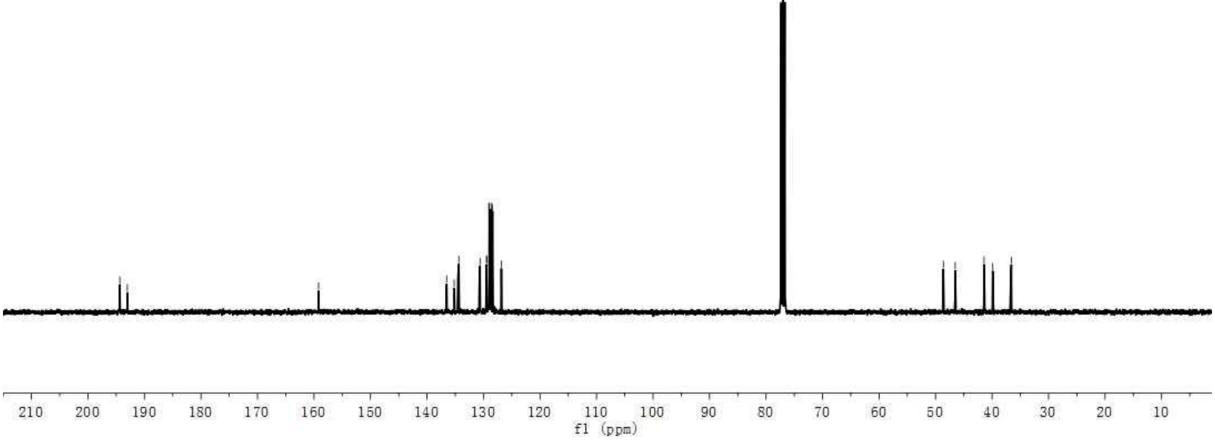
194.371
193.036
159.196
136.520
135.192
134.527
134.370
130.729
130.625
129.489
129.377
129.073
128.968
128.658
128.412
128.380
126.847
48.617
46.525
41.410
39.883
36.597
210 200 190 180 170 160 150 140 130 120 110 100 90 80 70 60 50 40 30 20 10
f1 (ppm)

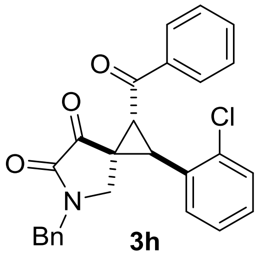
O
O
O
Cl
N
Bn

3h

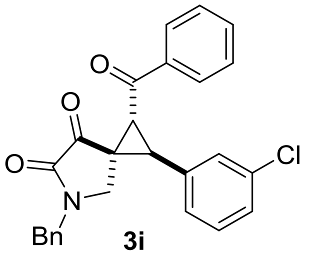

3i

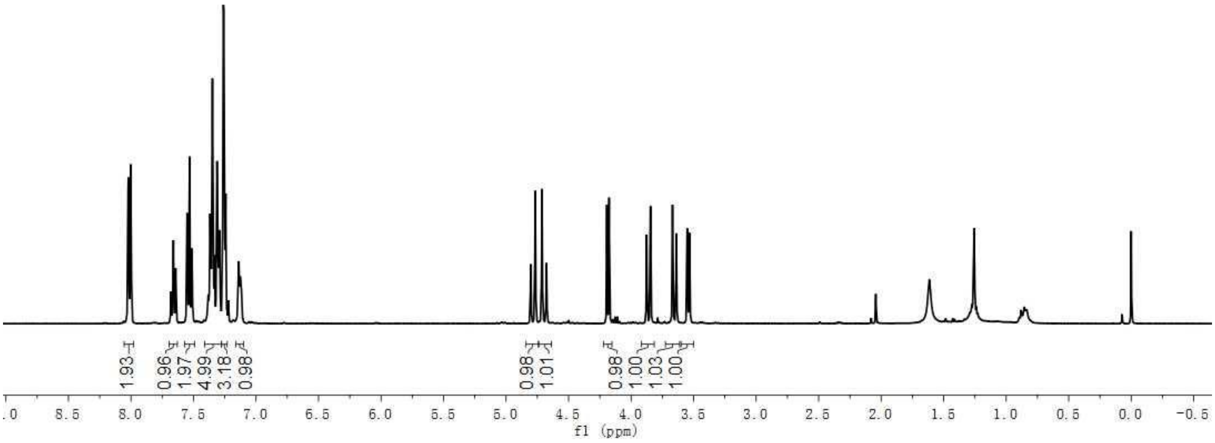
1.93
0.96
1.97
4.99
3.18
0.98
0.98
1.01
0.98
1.00
1.03
1.00
.0 8.5 8.0 7.5 7.0 6.5 6.0 5.5 5.0 4.5 4.0 3.5 3.0 2.5 2.0 1.5 1.0 0.5 0.0 -0.5
f1 (ppm)

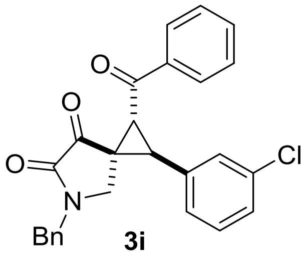

3i

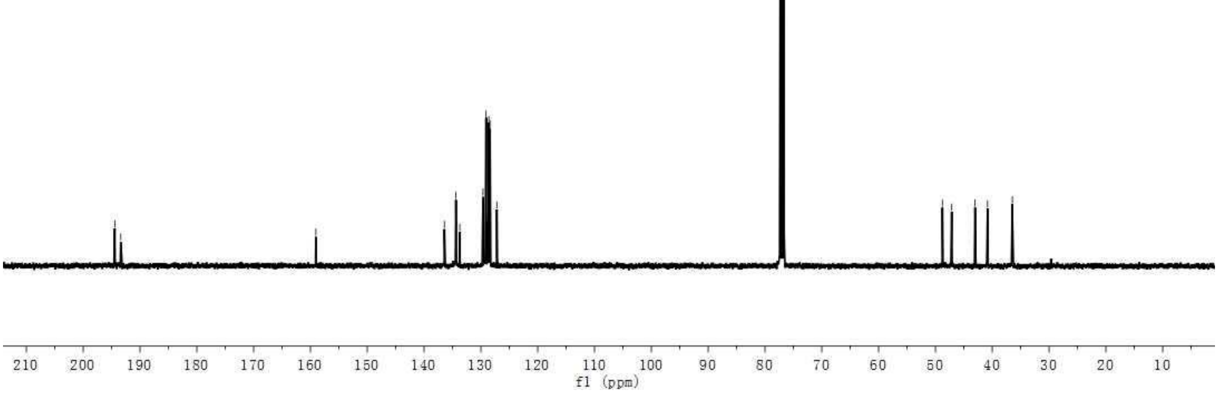
194.442
193.390
159.045
136.428
134.398
134.378
134.332
133.719
129.632
129.129
129.090
129.057
128.605
128.446
128.407
128.346
127.198
48.773
47.135
43.023
40.823
36.460
210 200 190 180 170 160 150 140 130 120 110 100 90 80 70 60 50 40 30 20 10
f1 (ppm)

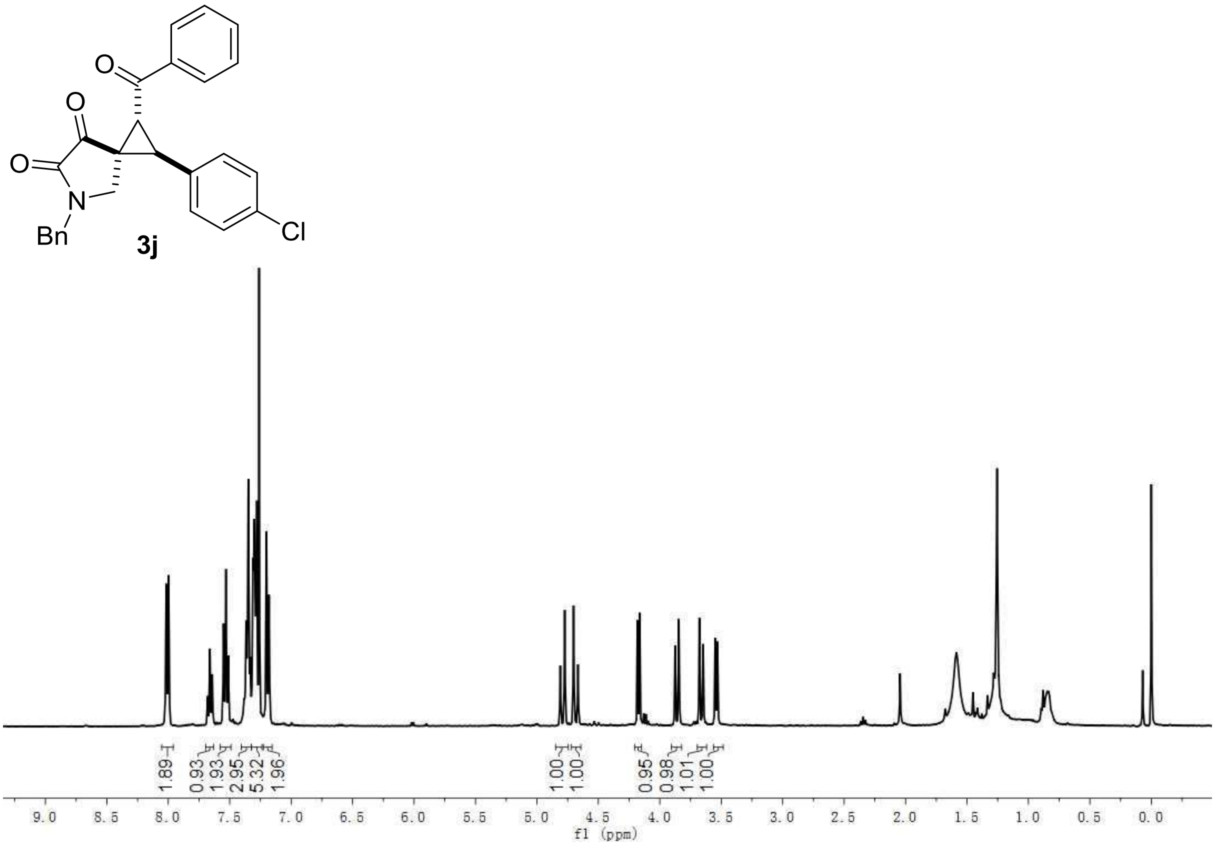
O
O
O
N
Bn
Cl
3j
1.89
0.93
1.93
2.95
5.32
1.96
1.00
1.00
0.95
0.98
1.01
1.00
9.0 8.5 8.0 7.5 7.0 6.5 6.0 5.5 5.0 4.5 4.0 3.5 3.0 2.5 2.0 1.5 1.0 0.5 0.0
f1 (ppm)

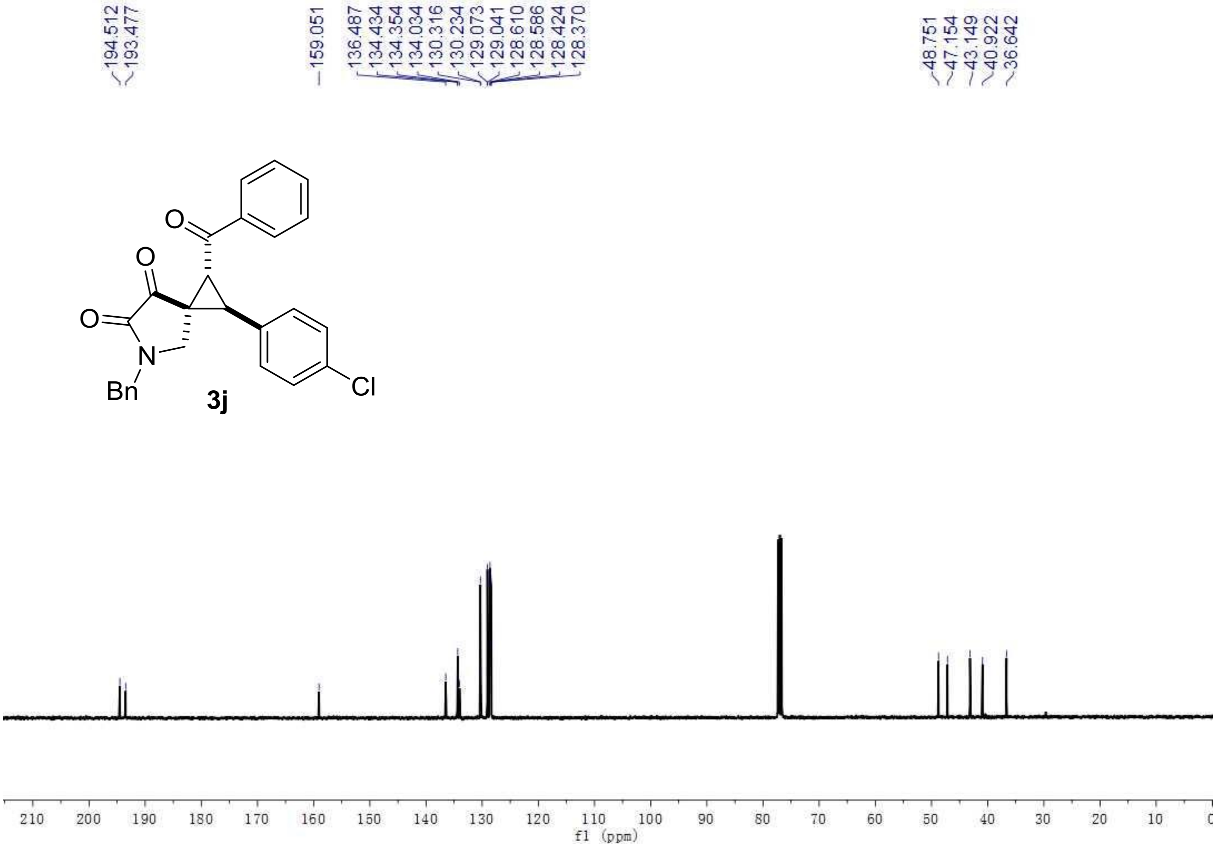
194.512
193.477
159.051
136.487
134.434
134.354
134.034
130.316
130.234
129.073
129.041
128.610
128.586
128.424
128.370
48.751
47.154
43.149
40.922
36.642
O
O
O
N
Bn
Cl
3j
210 200 190 180 170 160 150 140 130 120 110 100 90 80 70 60 50 40 30 20 10 0
f1 (ppm)

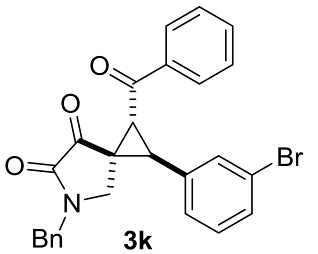
O
O
O
Br
N
Bn

3k

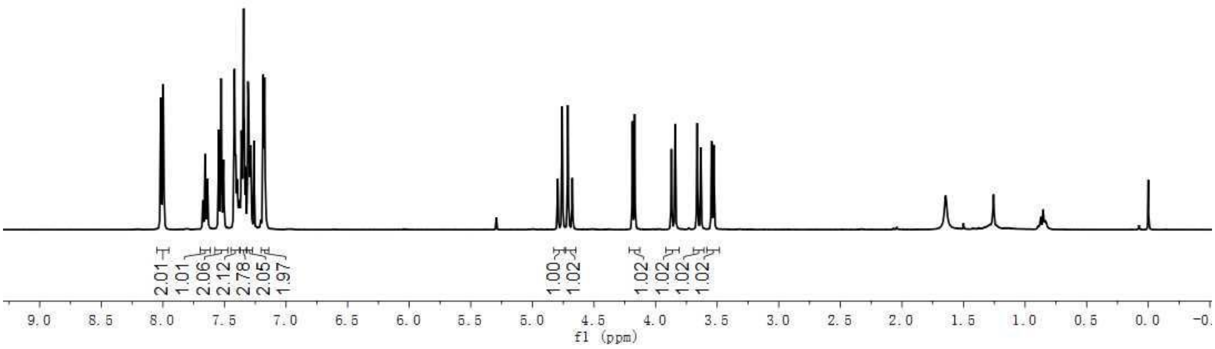
2.01
1.01
2.06
2.12
2.78
2.05
1.97
1.00
1.02
1.02
1.02
1.02
1.02
9.0 8.5 8.0 7.5 7.0 6.5 6.0 5.5 5.0 4.5 4.0 3.5 3.0 2.5 2.0 1.5 1.0 0.5 0.0
f1 (ppm)

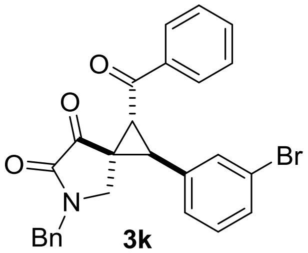
O
O
O
Br
N
Bn

3k

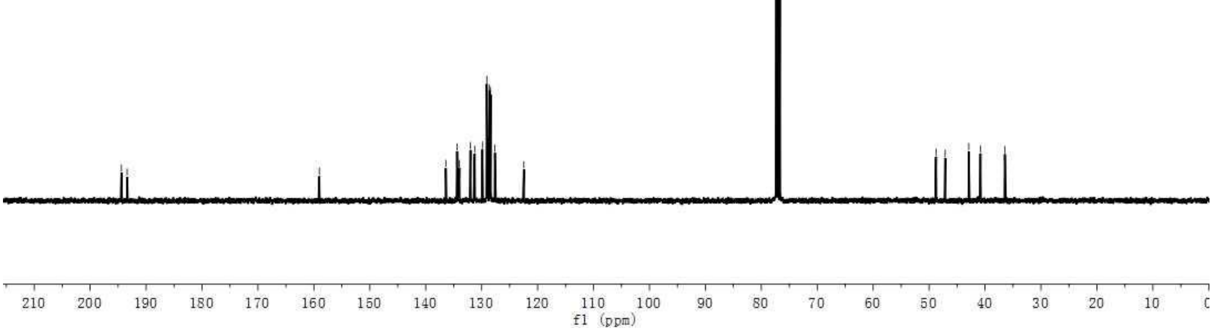
194.403
193.382
159.032
136.411
134.389
134.367
133.986
131.994
131.252
129.869
129.079
129.046
128.599
128.434
128.404
127.638
122.448
48.765
47.117
42.912
40.826
36.451
210 200 190 180 170 160 150 140 130 120 110 100 90 80 70 60 50 40 30 20 10
f1 (ppm)

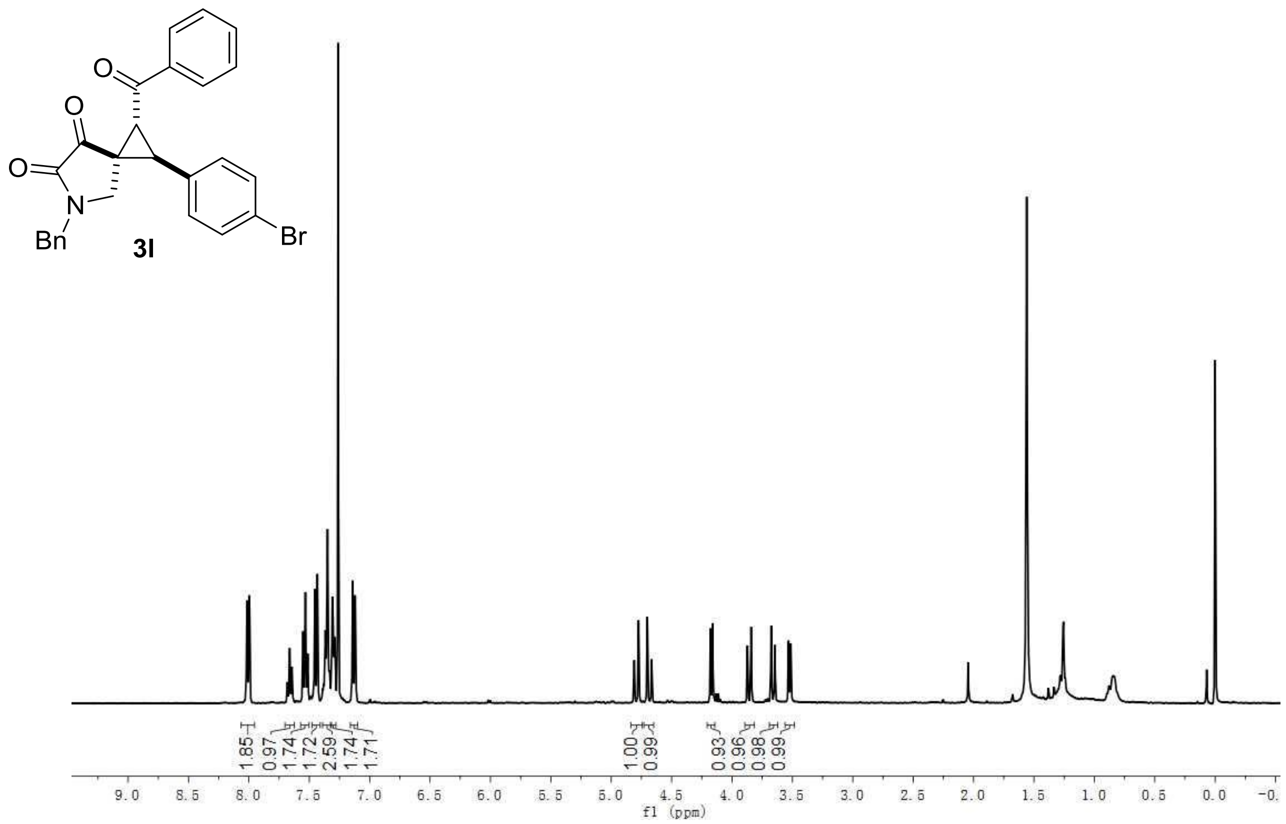
O
O
O
N
Bn
3l
Br
1.85
0.97
1.74
1.72
2.59
1.74
1.71
1.00
0.99
0.93
0.96
0.98
0.99
f1 (ppm)

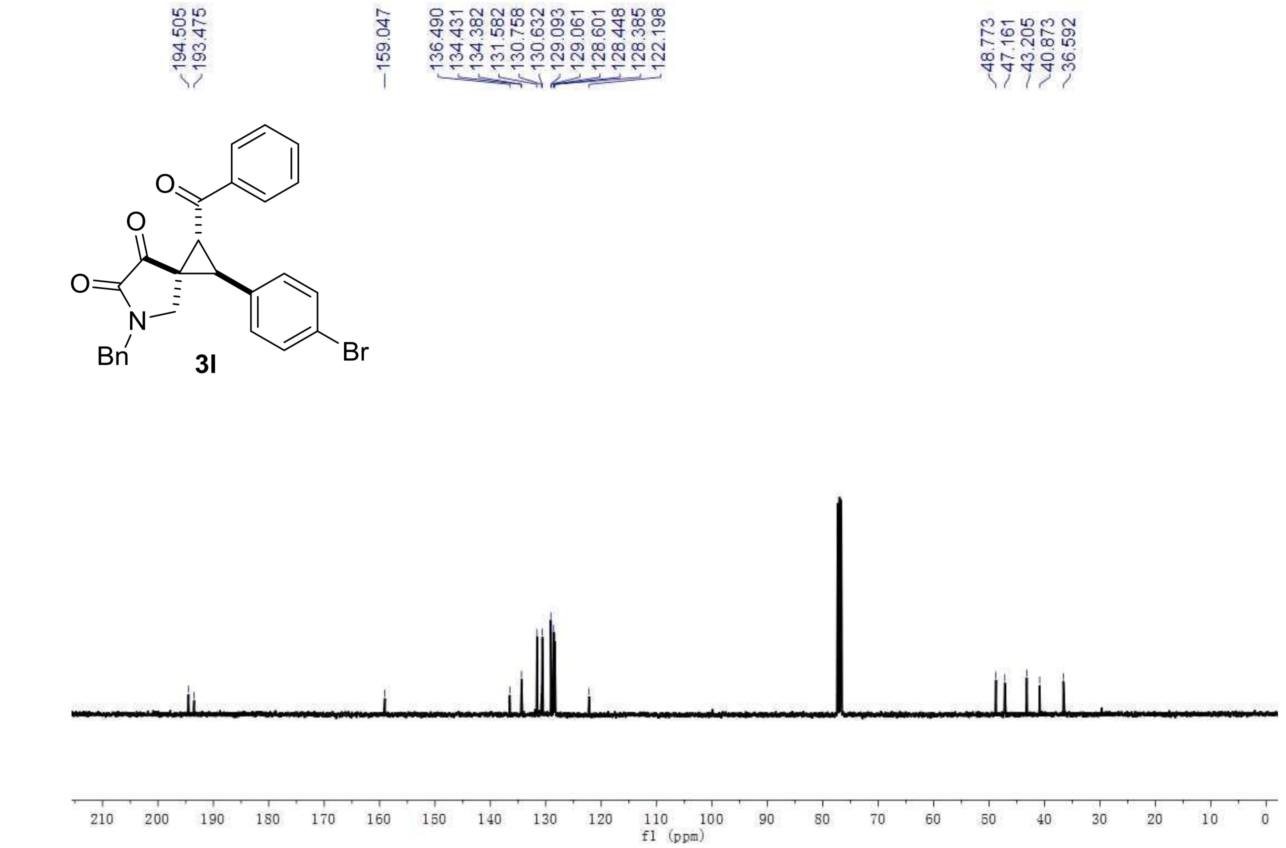
194.505
193.475
159.047
136.490
134.431
134.382
131.582
130.758
130.632
129.093
129.061
128.601
128.448
128.385
122.198
48.773
47.161
43.205
40.873
36.592
O
O
O
N
Bn
3l
Br
f1 (ppm)

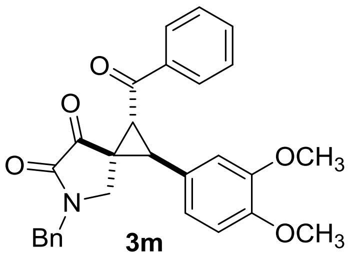
O
O
O
N
Bn
OCH3
OCH3

3m

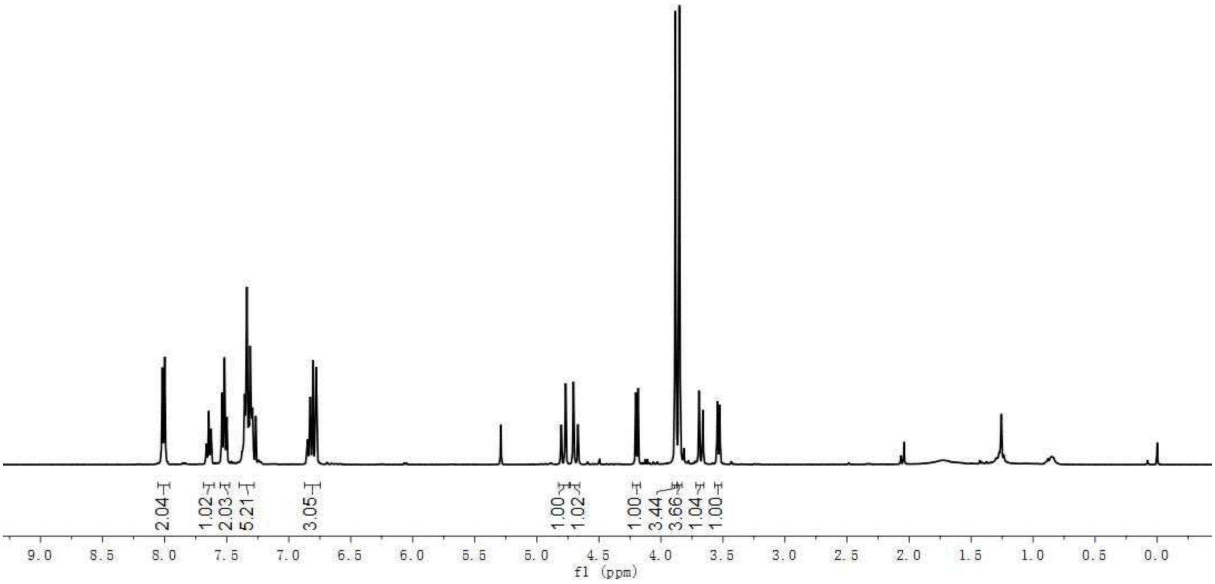
2.04
1.02
2.03
5.21
3.05
1.00
1.02
1.00
3.44
3.66
1.04
1.00
9.0 8.5 8.0 7.5 7.0 6.5 6.0 5.5 5.0 4.5 4.0 3.5 3.0 2.5 2.0 1.5 1.0 0.5 0.0
f1 (ppm)

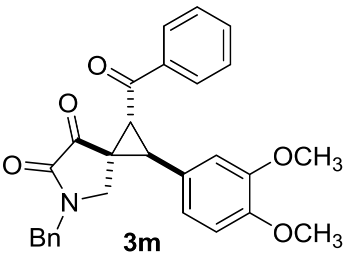
O
O
O
N
Bn
OCH3
OCH3

3m

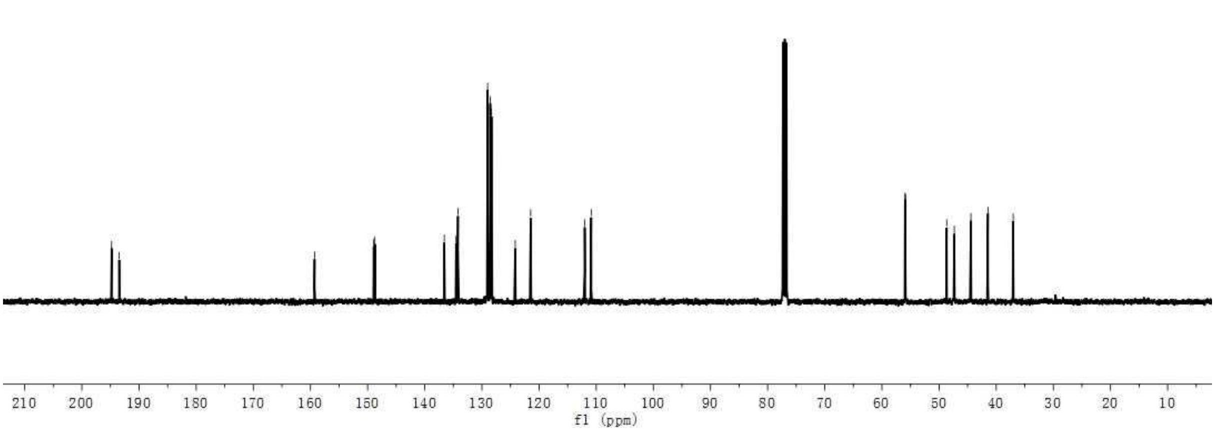
194.802
193.472
159.279
148.925
148.728
136.583
134.523
134.191
129.001
128.962
128.541
128.345
128.319
124.165
121.468
112.007
110.873
55.958
55.863
48.661
47.338
44.427
41.444
37.031
210 200 190 180 170 160 150 140 130 120 110 100 90 80 70 60 50 40 30 20 10
f1 (ppm)

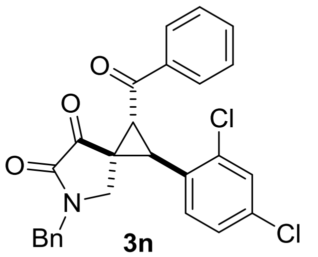
O
O
O
Cl
N
Bn
Cl
3n

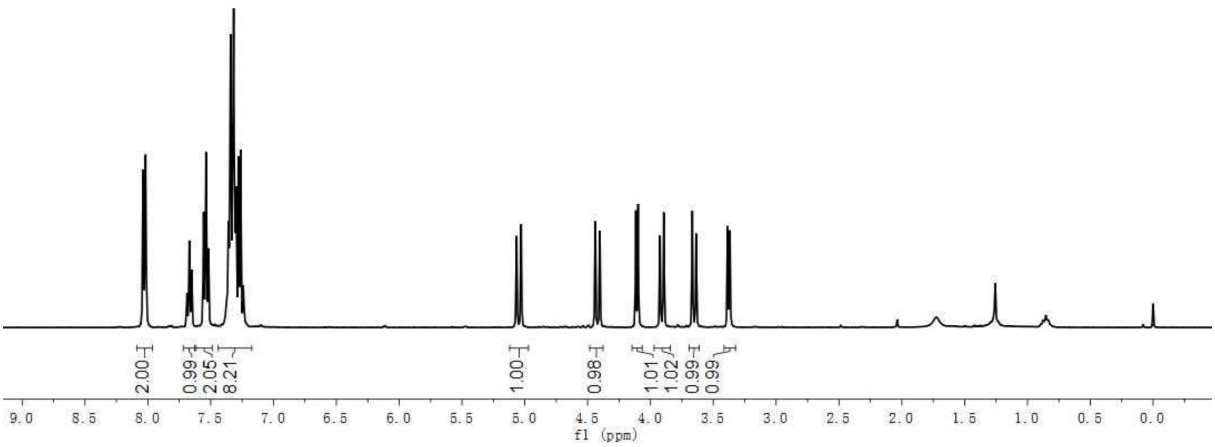
2.00
0.99
2.05
8.21
1.00
0.98
1.01
1.02
0.99
0.99
9.0 8.5 8.0 7.5 7.0 6.5 6.0 5.5 5.0 4.5 4.0 3.5 3.0 2.5 2.0 1.5 1.0 0.5 0.0
f1 (ppm)

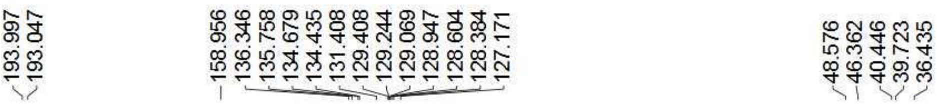
193.997
193.047
158.956
136.346
135.758
134.679
134.435
131.408
129.408
129.244
129.069
128.947
128.604
128.384
127.171
48.576
46.362
40.446
39.723
36.435

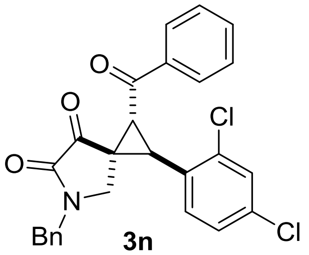
O
O
O
Cl
N
Bn
Cl
3n

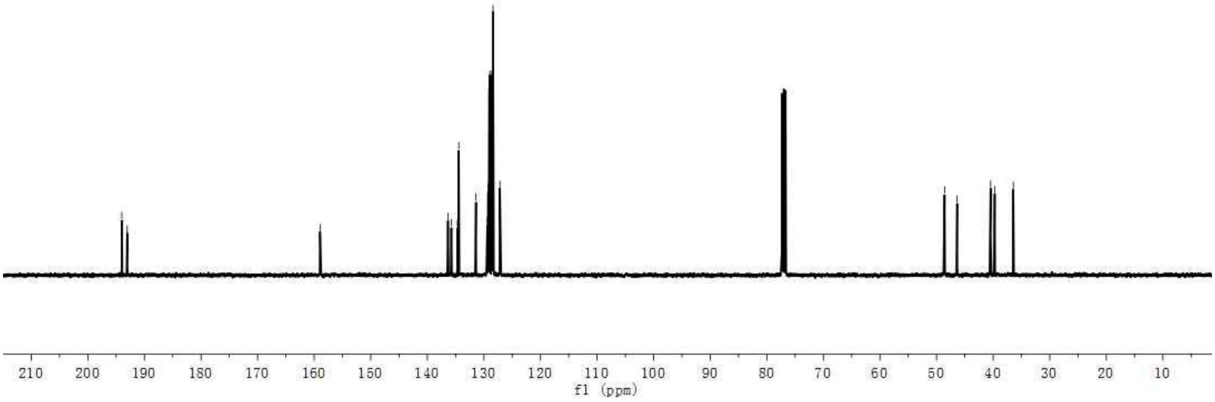
210 200 190 180 170 160 150 140 130 120 110 100 90 80 70 60 50 40 30 20 10
f1 (ppm)

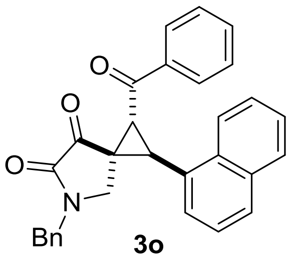

3o

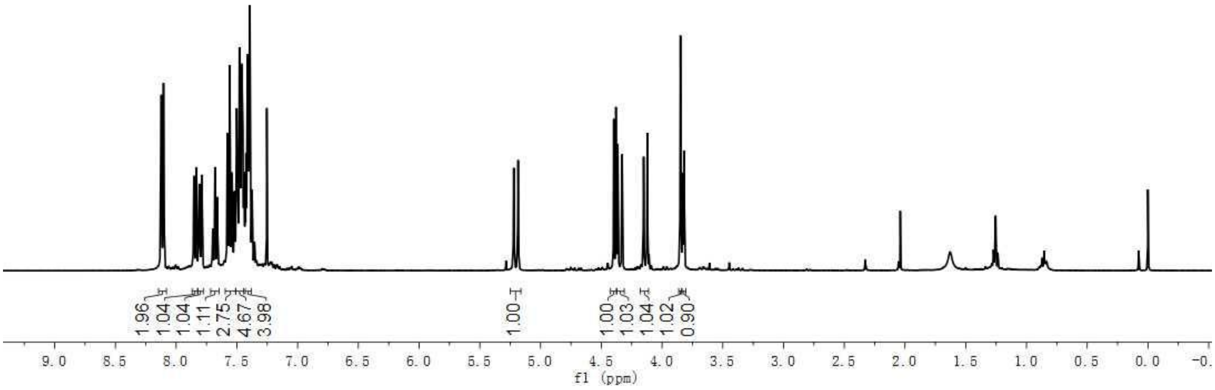
1.96
1.04
1.04
1.11
2.75
4.67
3.98
1.00
1.00
1.03
1.04
1.02
0.90
9.0 8.5 8.0 7.5 7.0 6.5 6.0 5.5 5.0 4.5 4.0 3.5 3.0 2.5 2.0 1.5 1.0 0.5 0.0
f1 (ppm)

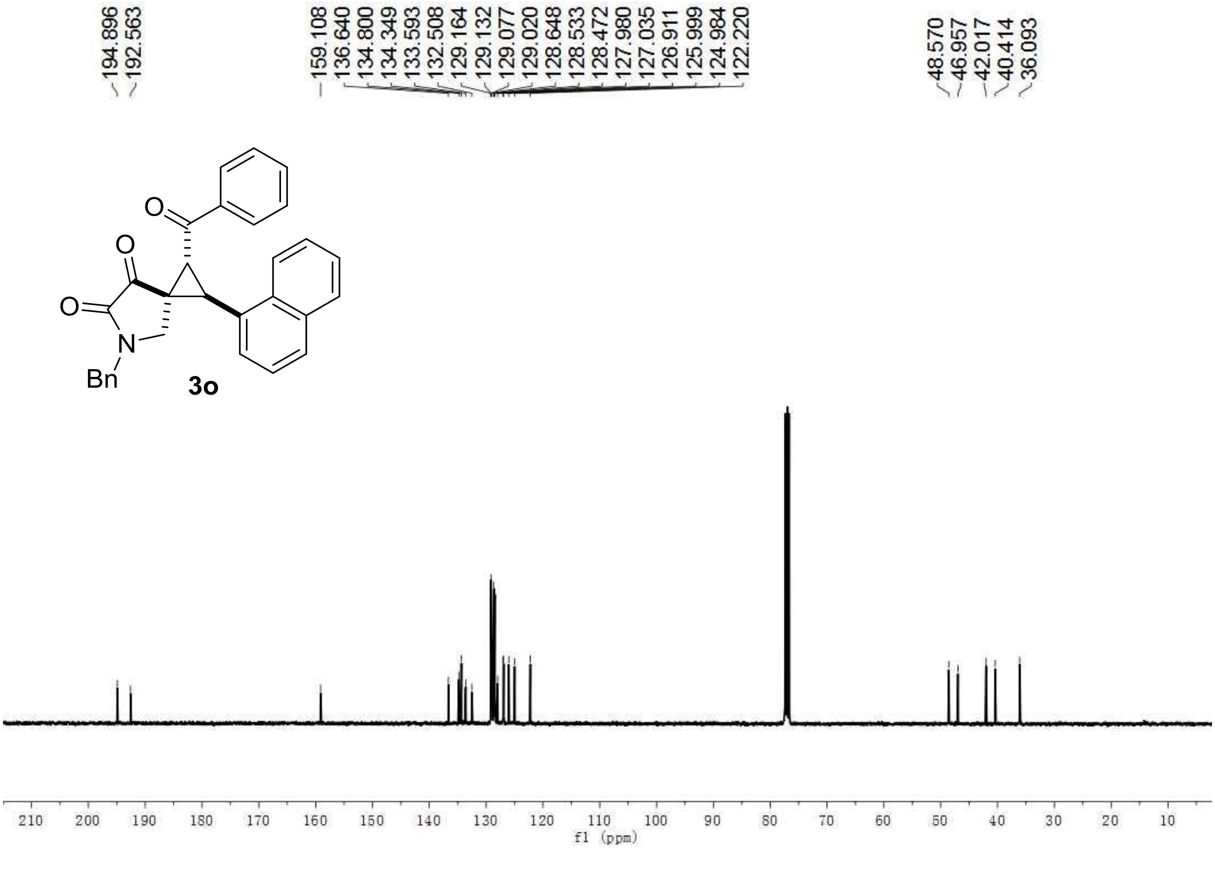
194.896
192.563
159.108
136.640
134.800
134.349
133.593
132.508
129.164
129.132
129.077
129.020
128.648
128.533
128.472
127.980
127.035
126.911
125.999
124.984
122.220
48.570
46.957
42.017
40.414
36.093
210 200 190 180 170 160 150 140 130 120 110 100 90 80 70 60 50 40 30 20 10
f1 (ppm)

3o

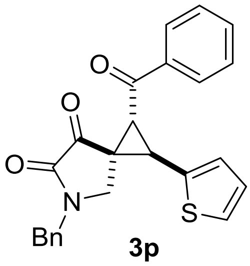
O
O
O
N
Bn
S

3p

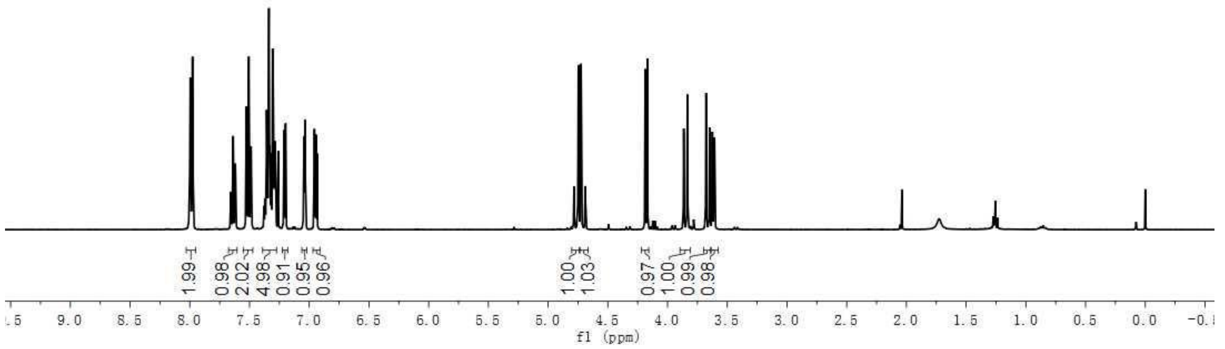
1.99
0.98
2.02
4.98
0.91
0.95
0.96
1.00
1.03
0.97
1.00
0.99
0.98
9.0 8.5 8.0 7.5 7.0 6.5 6.0 5.5 5.0 4.5 4.0 3.5 3.0 2.5 2.0 1.5 1.0 0.5 0.0
f1 (ppm)

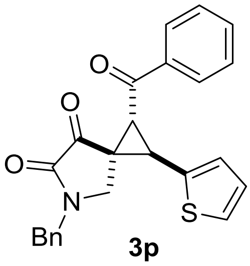
O
O
O
N
Bn
S

3p

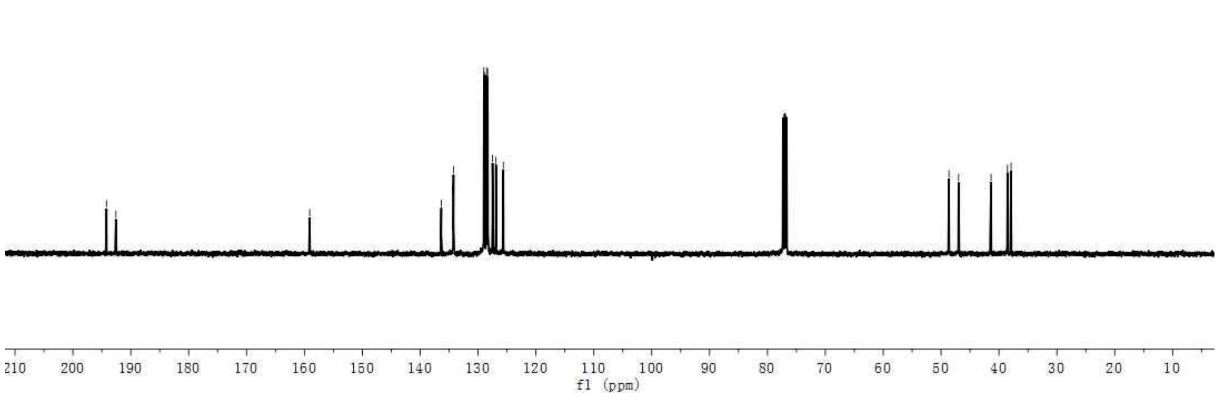
194.191
192.574
159.088
136.359
134.394
134.380
134.249
128.990
128.931
128.515
128.340
128.312
127.501
126.905
125.636
48.640
46.974
41.359
38.532
37.905
210 200 190 180 170 160 150 140 130 120 110 100 90 80 70 60 50 40 30 20 10
f1 (ppm)

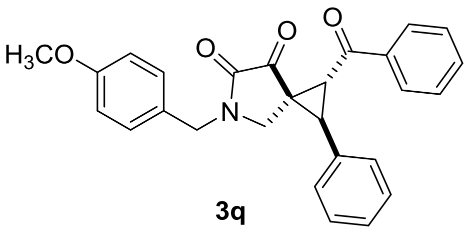

3q

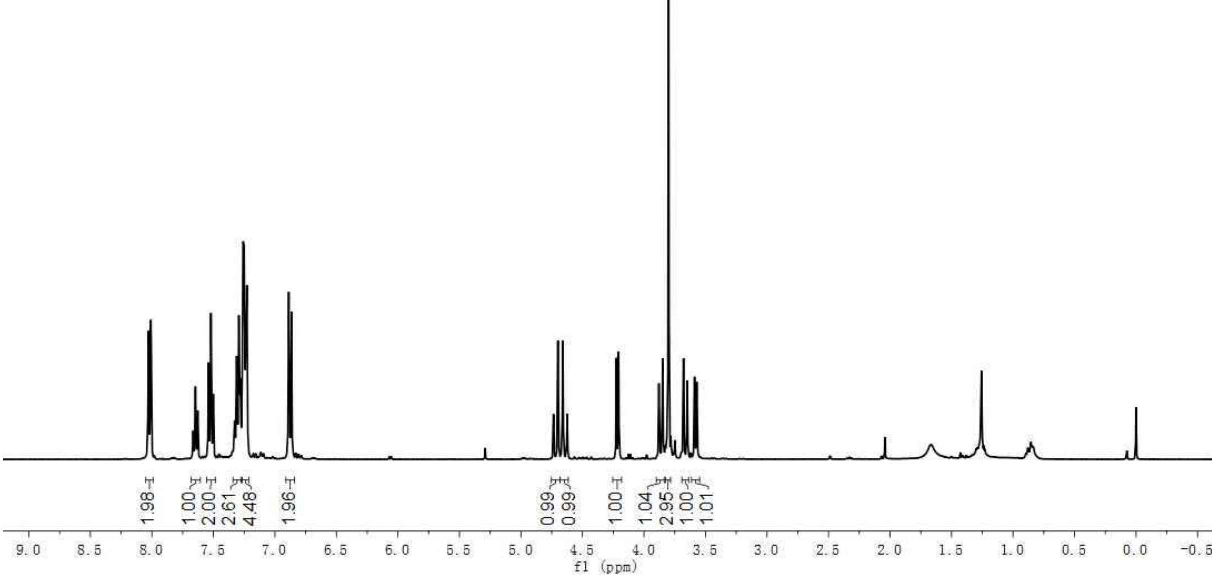
1.98
1.00
2.00
2.61
4.48
1.96
0.99
0.99
1.00
1.04
2.95
1.00
1.01
9.0 8.5 8.0 7.5 7.0 6.5 6.0 5.5 5.0 4.5 4.0 3.5 3.0 2.5 2.0 1.5 1.0 0.5 0.0 -0.5
f1 (ppm)

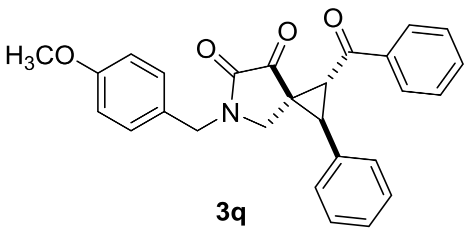

3q

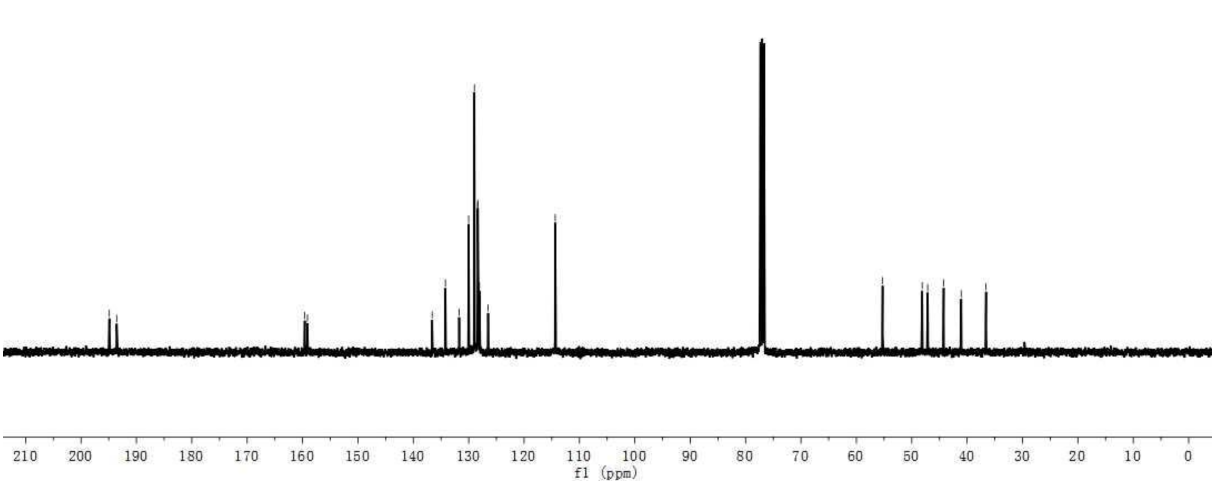
194.899
193.565
159.598
159.118
136.605
134.207
131.725
130.008
128.974
128.400
128.354
128.059
126.504
114.382
55.259
48.115
47.125
44.241
41.071
36.587
210 200 190 180 170 160 150 140 130 120 110 100 90 80 70 60 50 40 30 20 10 0
f1 (ppm)

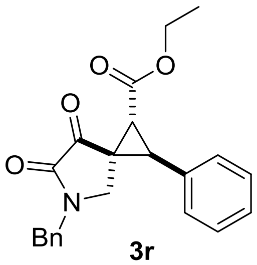
O
O
O
O
N
Bn

3r

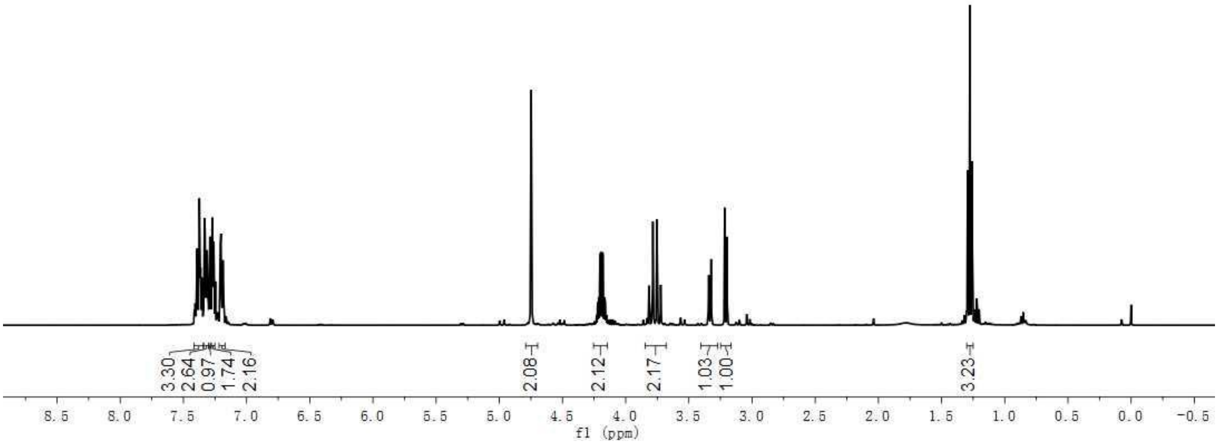
3.30
2.64
0.97
1.74
2.16
2.08
2.12
2.17
1.03
1.00
3.23
8.5 8.0 7.5 7.0 6.5 6.0 5.5 5.0 4.5 4.0 3.5 3.0 2.5 2.0 1.5 1.0 0.5 0.0 -0.5
f1 (ppm)

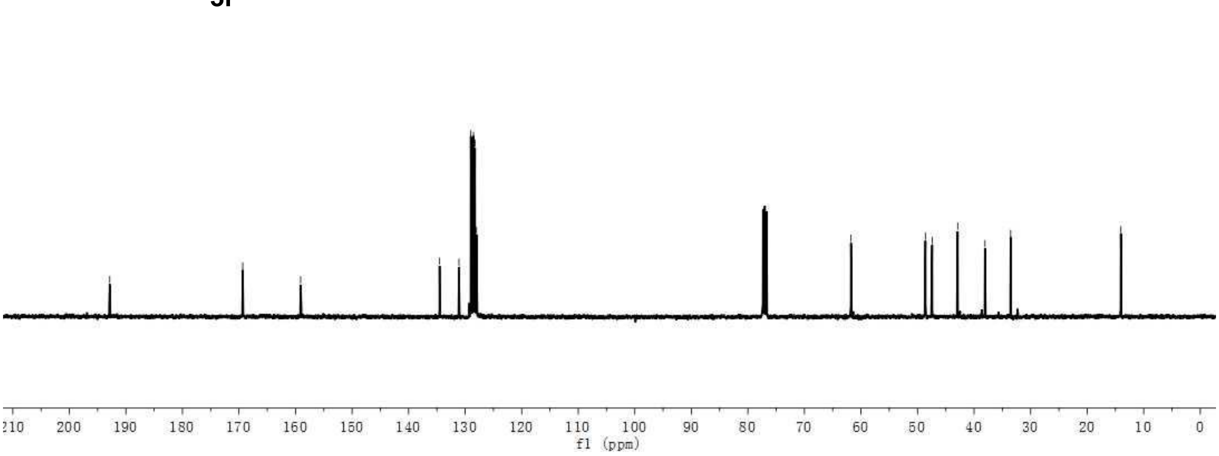
192.857
169.336
159.098
134.523
131.096
128.992
128.874
128.527
128.318
128.269
127.996
61.761
48.600
47.416
42.895
38.072
33.529
14.038
210 200 190 180 170 160 150 140 130 120 110 100 90 80 70 60 50 40 30 20 10 0
f1 (ppm)

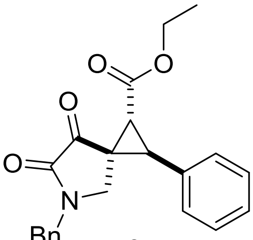
O
O
O
O
N
Bn

3r

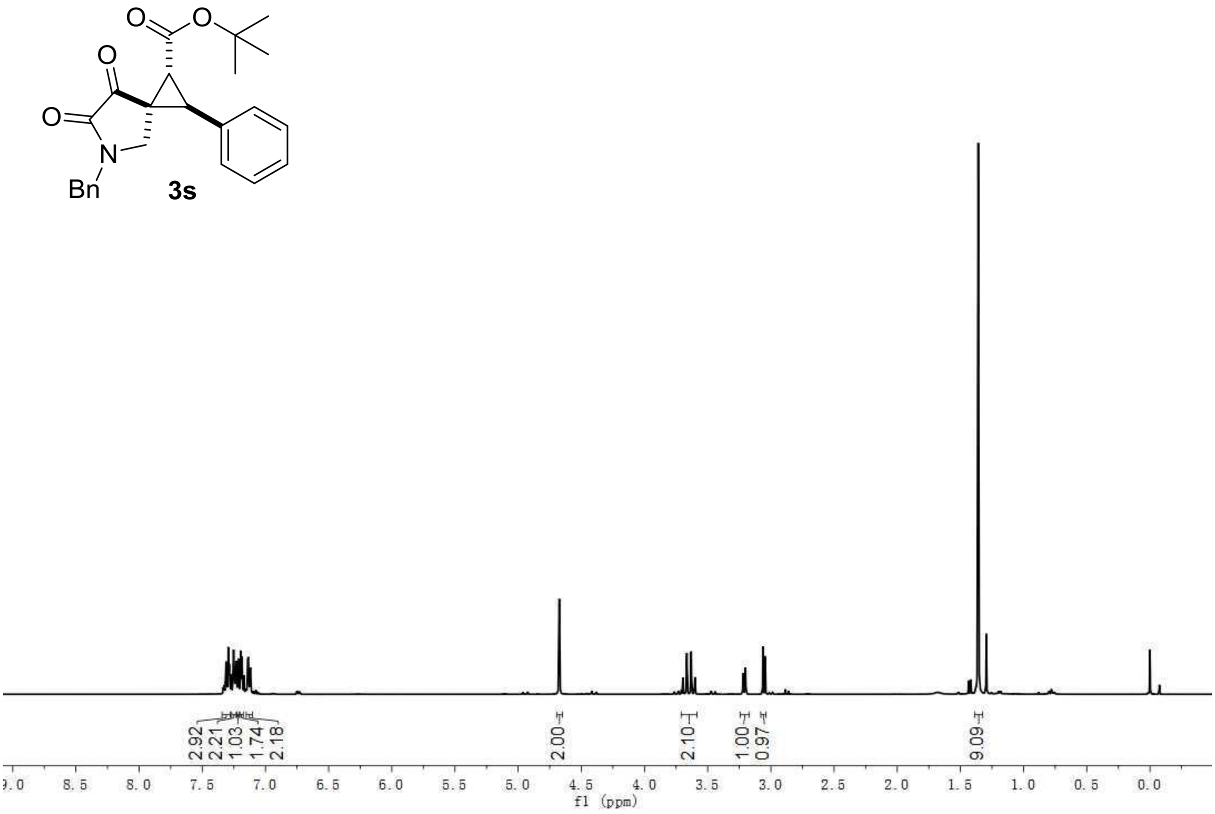

Bn
3s
2.92
2.21
1.03
1.74
2.18
2.00
2.10
1.00
0.97
9.09
f1 (ppm)

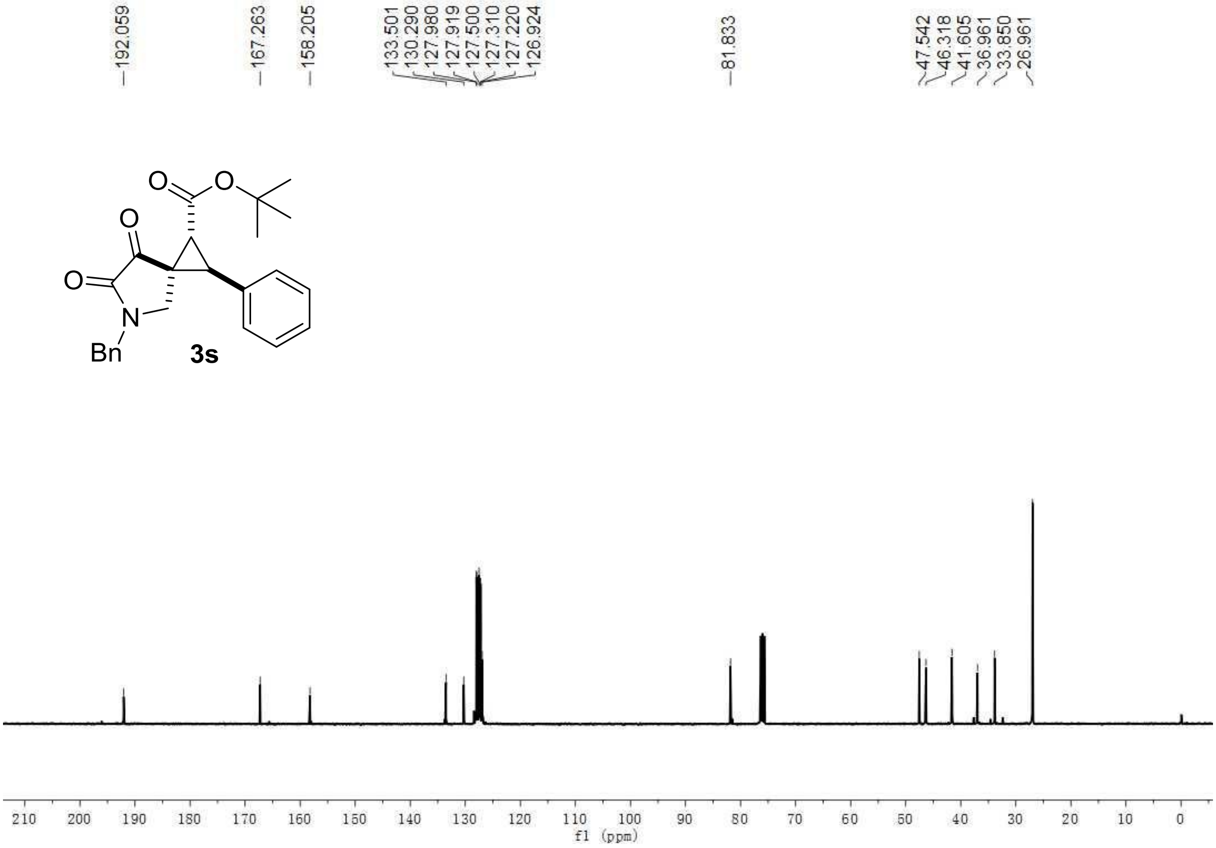

192.059
167.263
158.205
133.501
130.290
127.980
127.919
127.500
127.310
127.220
126.924
81.833
47.542
46.318
41.605
36.961
33.850
26.961
Bn
3s
f1 (ppm)

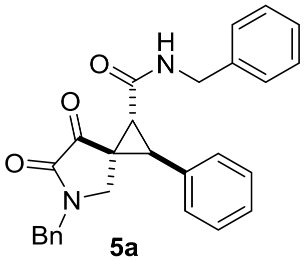
O
H
N
O
O
N
Bn

5a

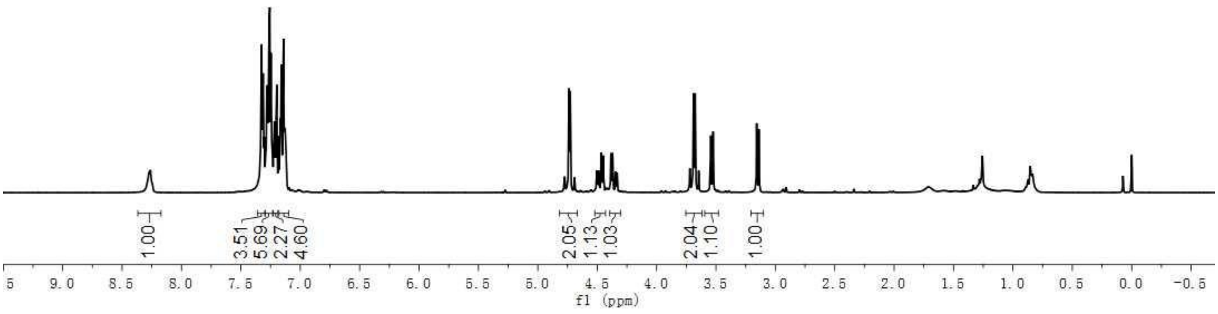
1.00
3.51
5.69
2.27
4.60
2.05
1.13
1.03
2.04
1.10
1.00
9.0 8.5 8.0 7.5 7.0 6.5 6.0 5.5 5.0 4.5 4.0 3.5 3.0 2.5 2.0 1.5 1.0 0.5 0.0 -0.5
f1 (ppm)

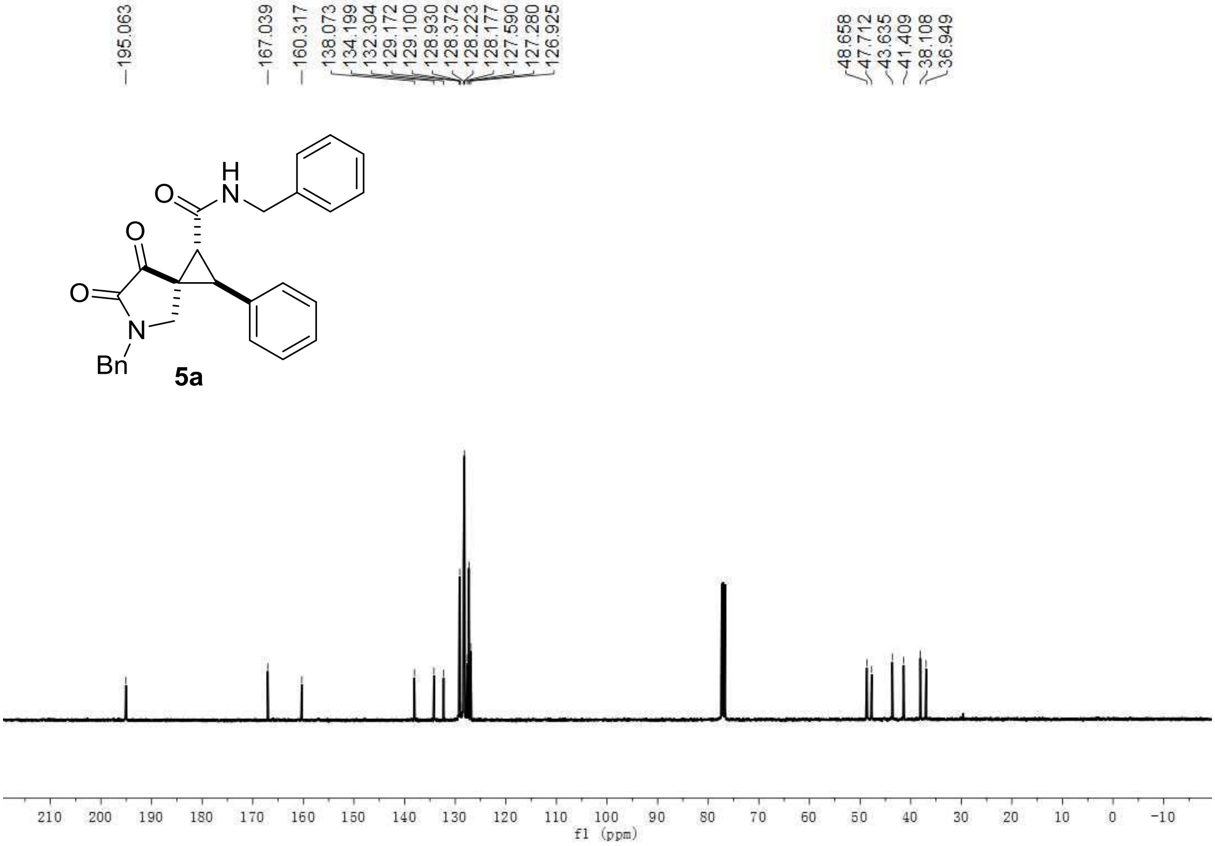
195.063
167.039
160.317
138.073
134.199
132.304
129.172
129.100
128.930
128.372
128.223
128.177
127.590
127.280
126.925
48.658
47.712
43.635
41.409
38.108
36.949
5a
210 200 190 180 170 160 150 140 130 120 110 100 90 80 70 60 50 40 30 20 10 0 -10
f1 (ppm)

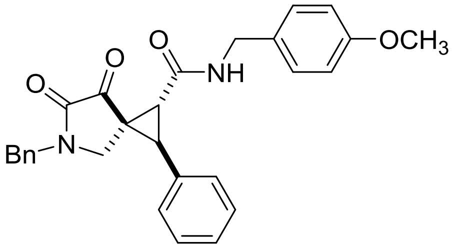
OCH3
NH
O
O
O
Bn−N

5b

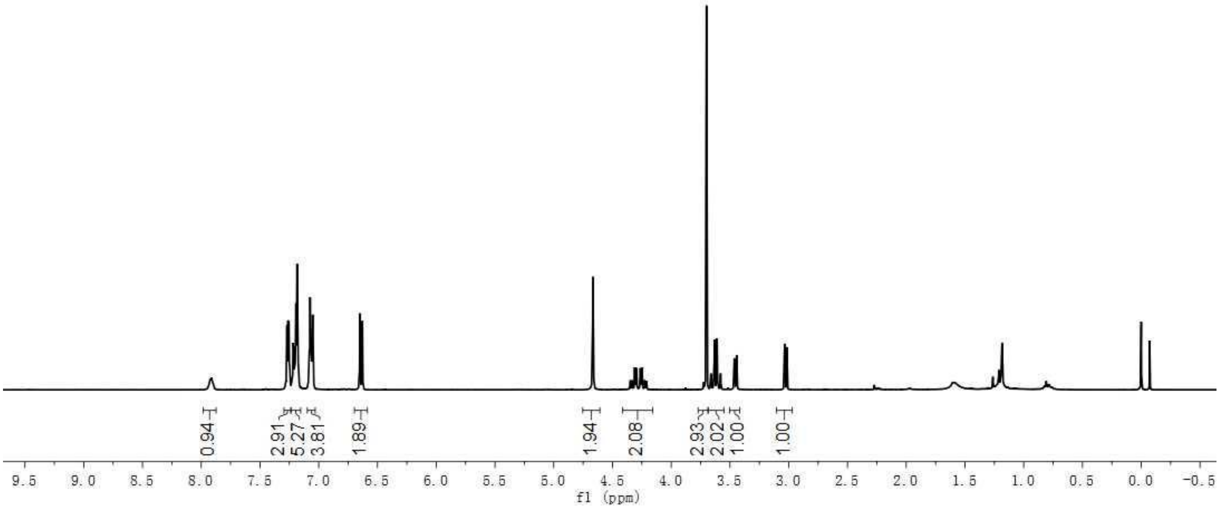
0.94
2.91
5.27
3.81
1.89
1.94
2.08
2.93
2.02
1.00
1.00
f1 (ppm)

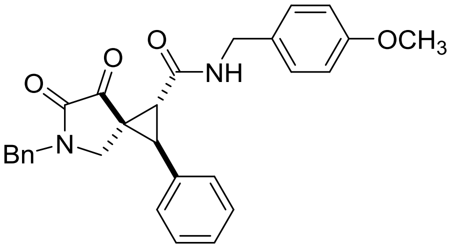
OCH3
NH
O
O
O
Bn−N

5b

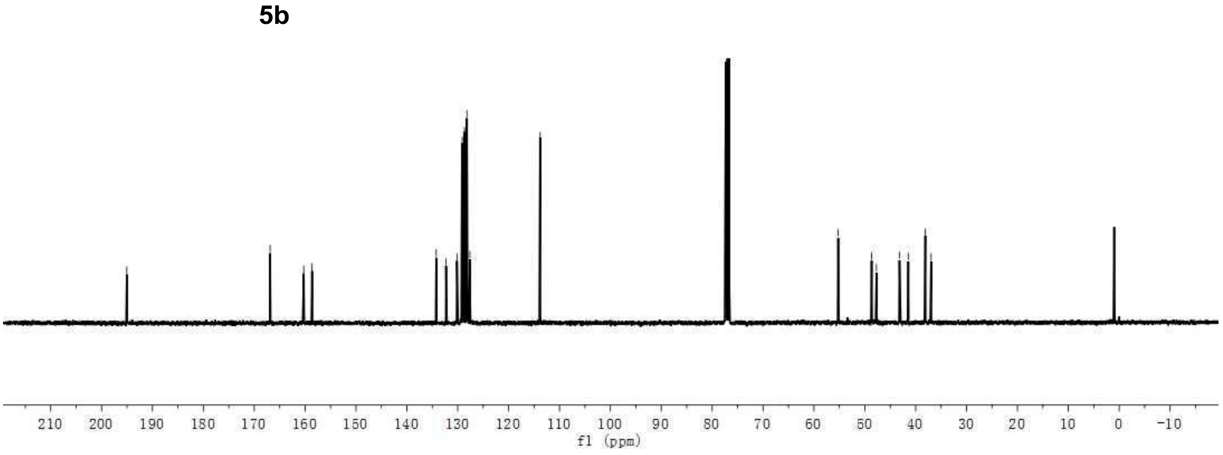
195.047
166.877
160.271
158.634
134.230
132.299
130.153
129.182
129.109
128.673
128.237
128.200
128.175
127.610
113.817
55.229
48.658
47.703
43.180
41.445
38.103
36.964
f1 (ppm)

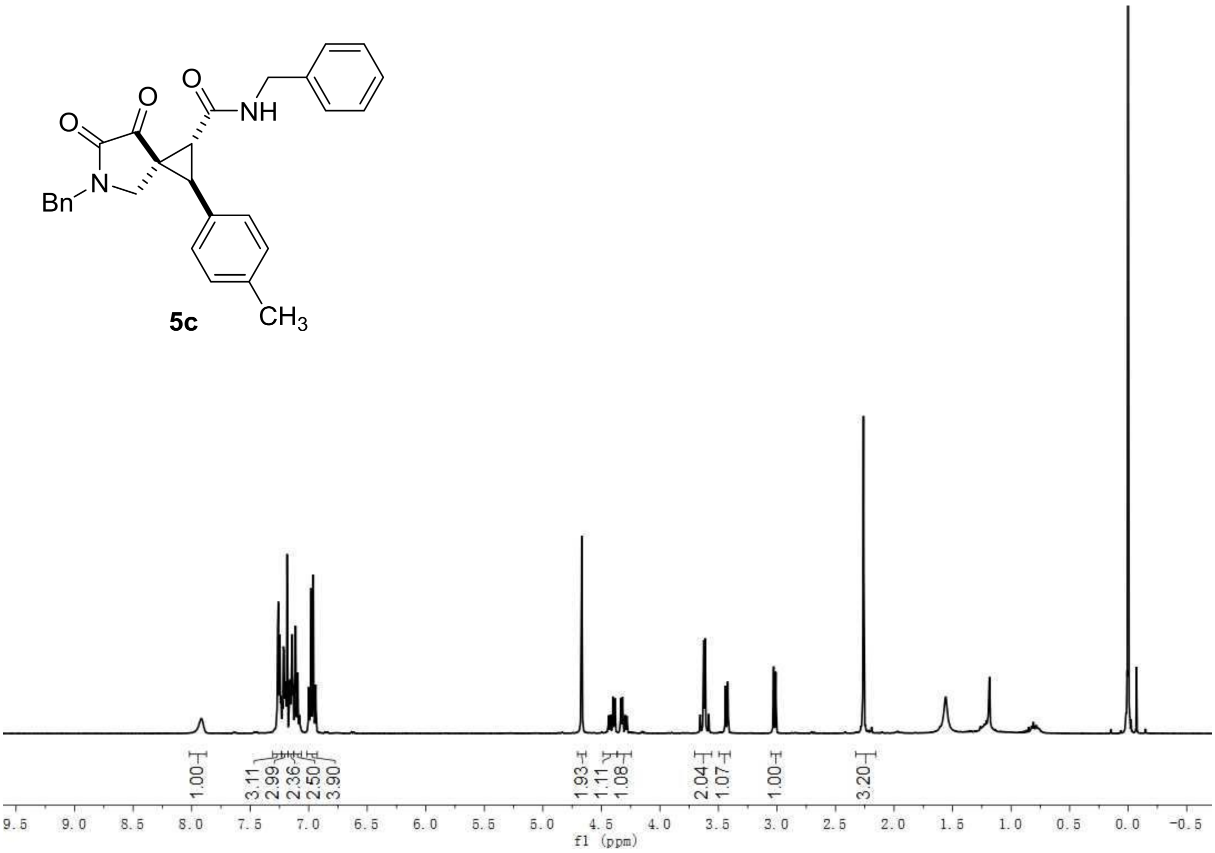

5c
1.00
3.11
2.99
2.36
2.50
3.90
1.93
1.11
1.08
2.04
1.07
1.00
3.20
f1 (ppm)

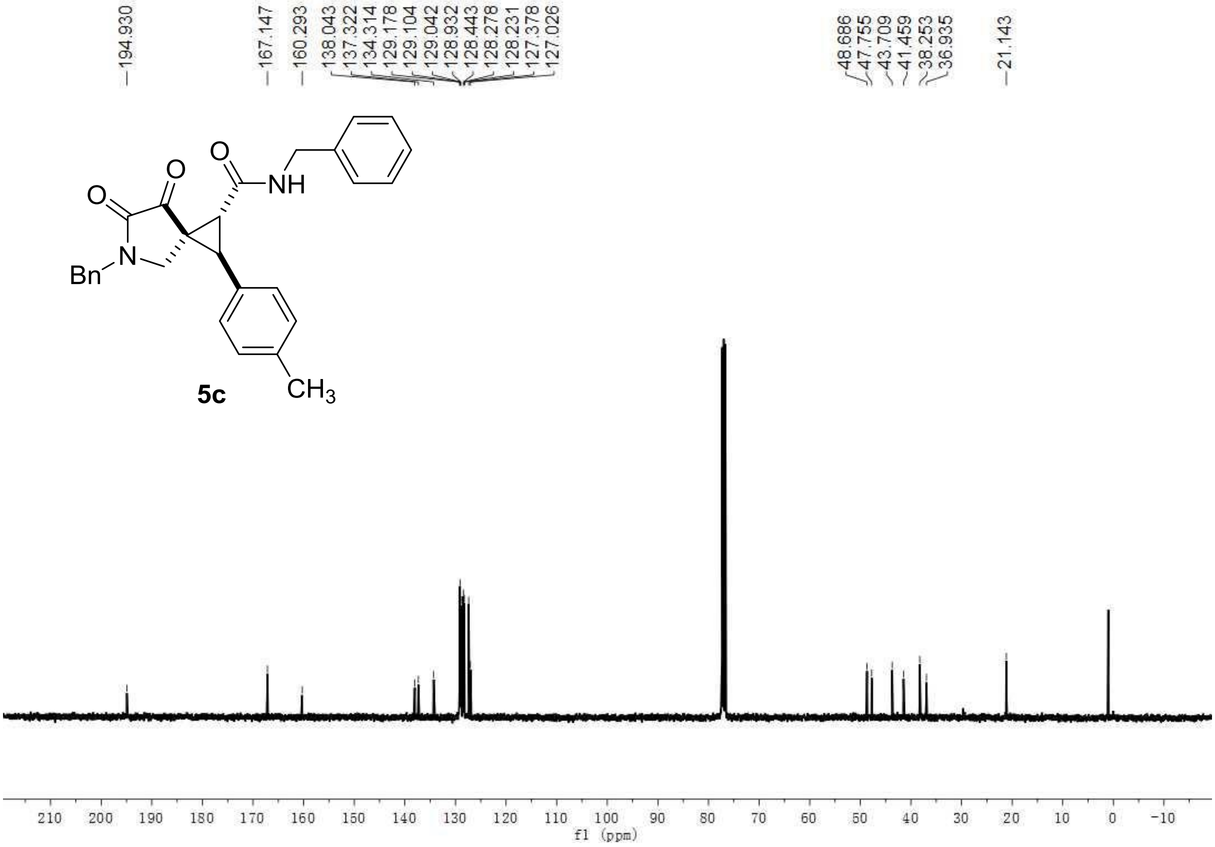

194.930
167.147
160.293
138.043
137.322
134.314
129.178
129.104
129.042
128.932
128.443
128.278
128.231
127.378
127.026
48.686
47.755
43.709
41.459
38.253
36.935
21.143
5c
f1 (ppm)

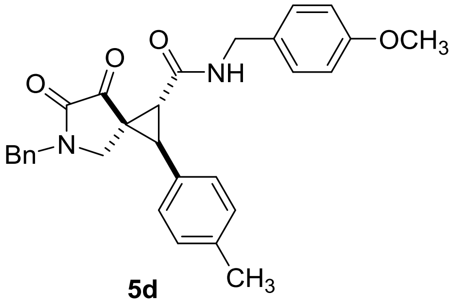
OCH3
NH
O
O
O
Bn
N
CH3

5d

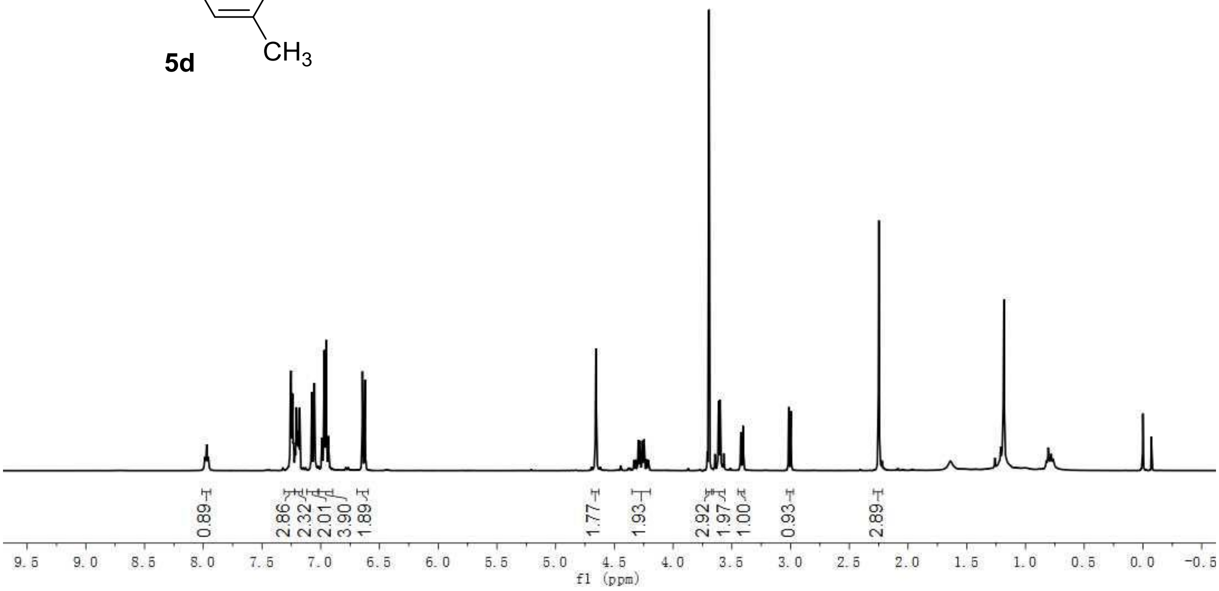
0.89
2.86
2.32
2.01
3.90
1.89
1.77
1.93
2.92
1.97
1.00
0.93
2.89
9.5 9.0 8.5 8.0 7.5 7.0 6.5 6.0 5.5 5.0 4.5 4.0 3.5 3.0 2.5 2.0 1.5 1.0 0.5 0.0 -0.5
f1 (ppm)

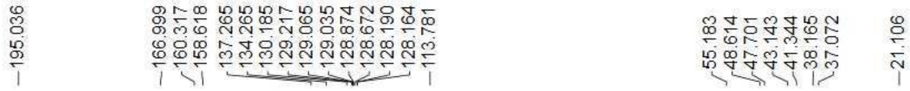
195.036
166.999
160.317
158.618
137.265
134.265
130.185
129.217
129.065
129.035
128.874
128.672
128.190
128.164
113.781
55.183
48.614
47.701
43.143
41.344
38.165
37.072
21.106

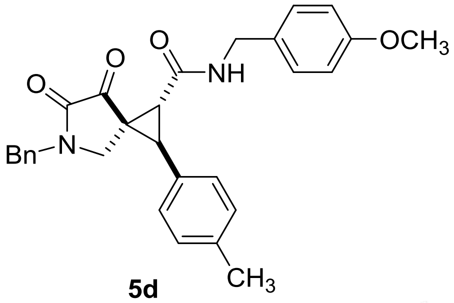
OCH3
NH
O
O
O
Bn
N
CH3

5d

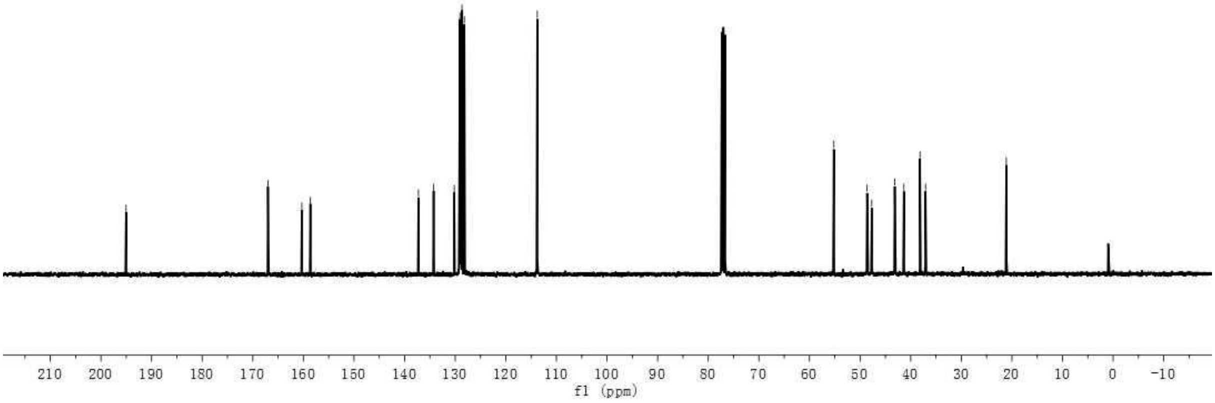
210 200 190 180 170 160 150 140 130 120 110 100 90 80 70 60 50 40 30 20 10 0 -10
f1 (ppm)

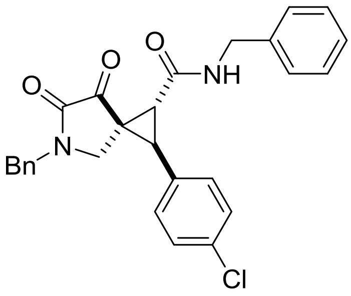

5e

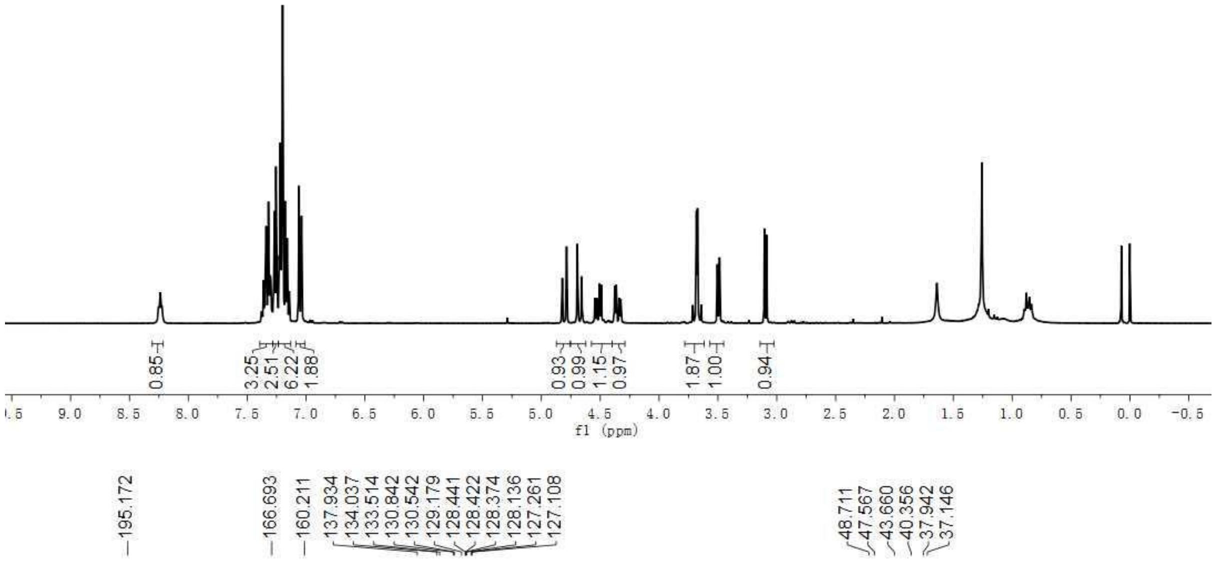

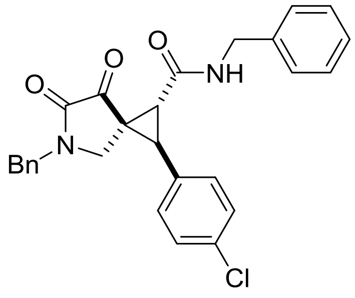

5e

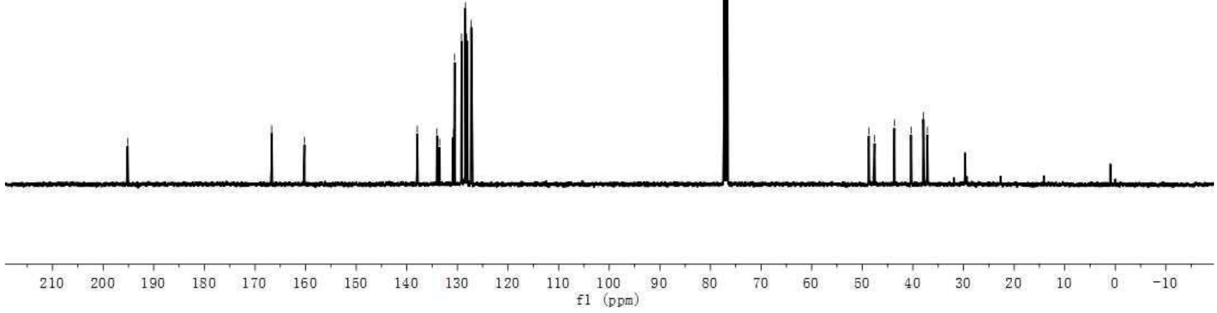

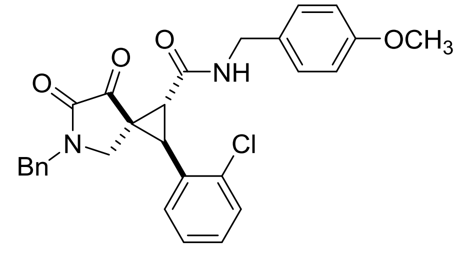

5f

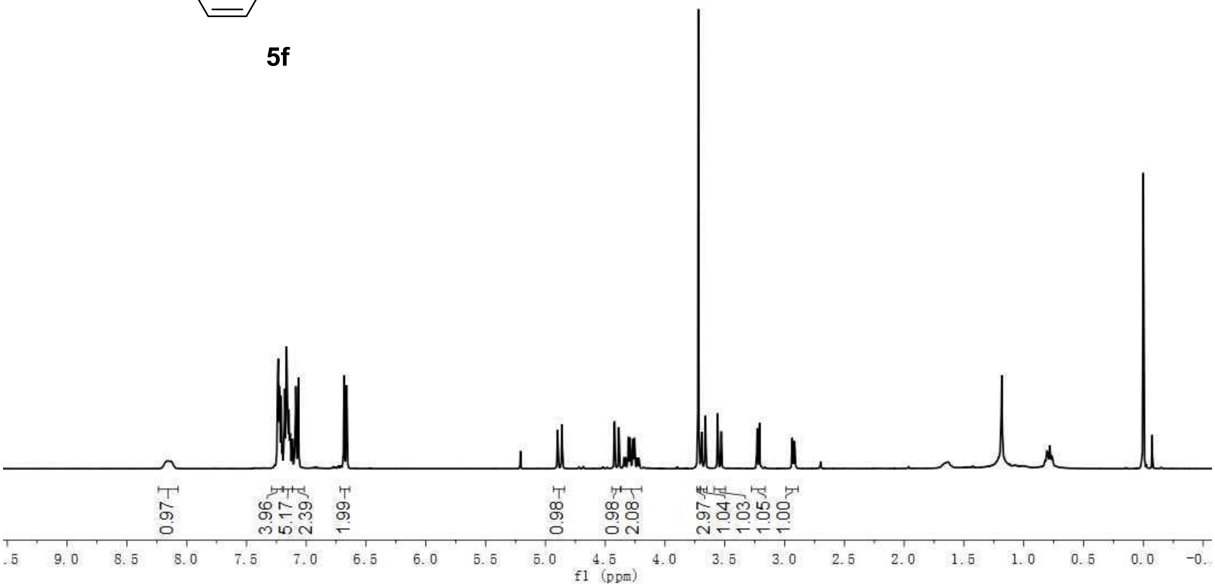
0.97
3.96
5.17
2.39
1.99
0.98
0.98
2.08
2.97
1.04
1.03
1.05
1.00
f1 (ppm)

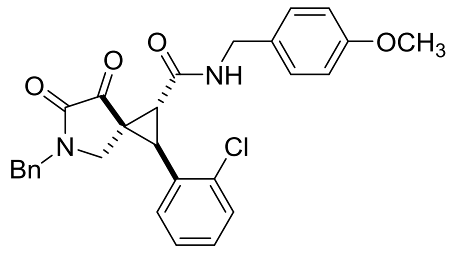

5f

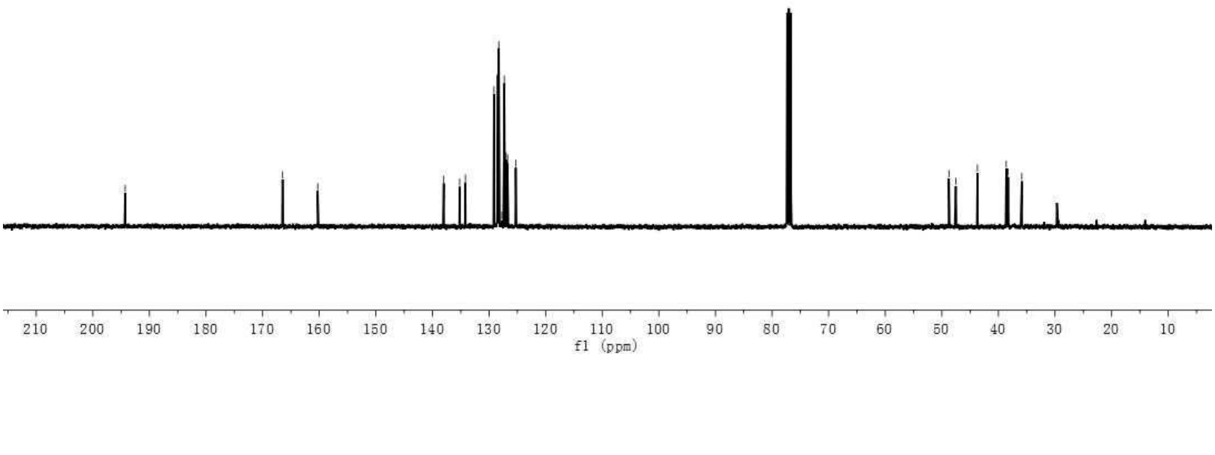
194.291
166.477
160.273
138.000
135.193
134.176
129.124
128.425
128.270
127.732
127.381
127.301
126.996
126.723
125.284
48.748
47.530
43.711
38.623
38.292
35.877
f1 (ppm)

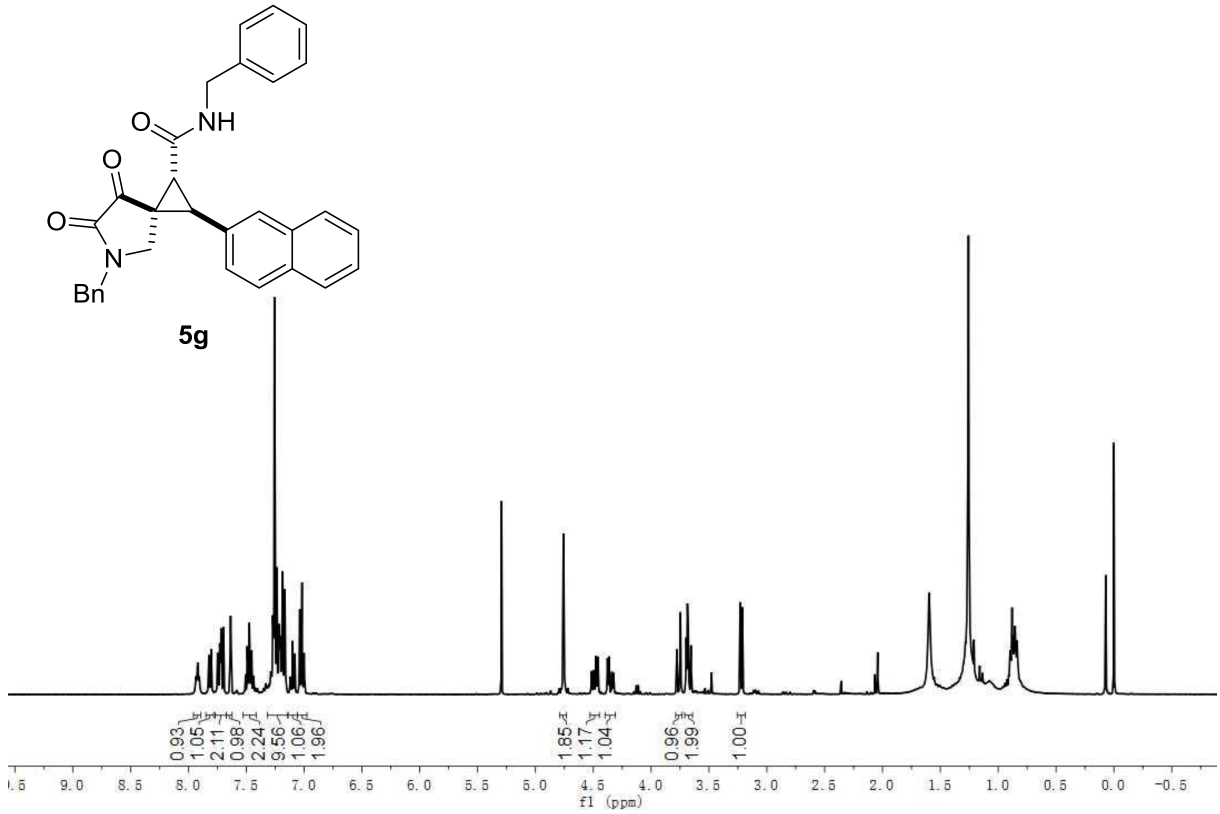
O
NH
O
O
N
Bn
5g
0.93
1.05
2.11
0.98
2.24
9.56
1.06
1.96
1.85
1.17
1.04
0.96
1.99
1.00
9.0 8.5 8.0 7.5 7.0 6.5 6.0 5.5 5.0 4.5 4.0 3.5 3.0 2.5 2.0 1.5 1.0 0.5 0.0 -0.5
f1 (ppm)

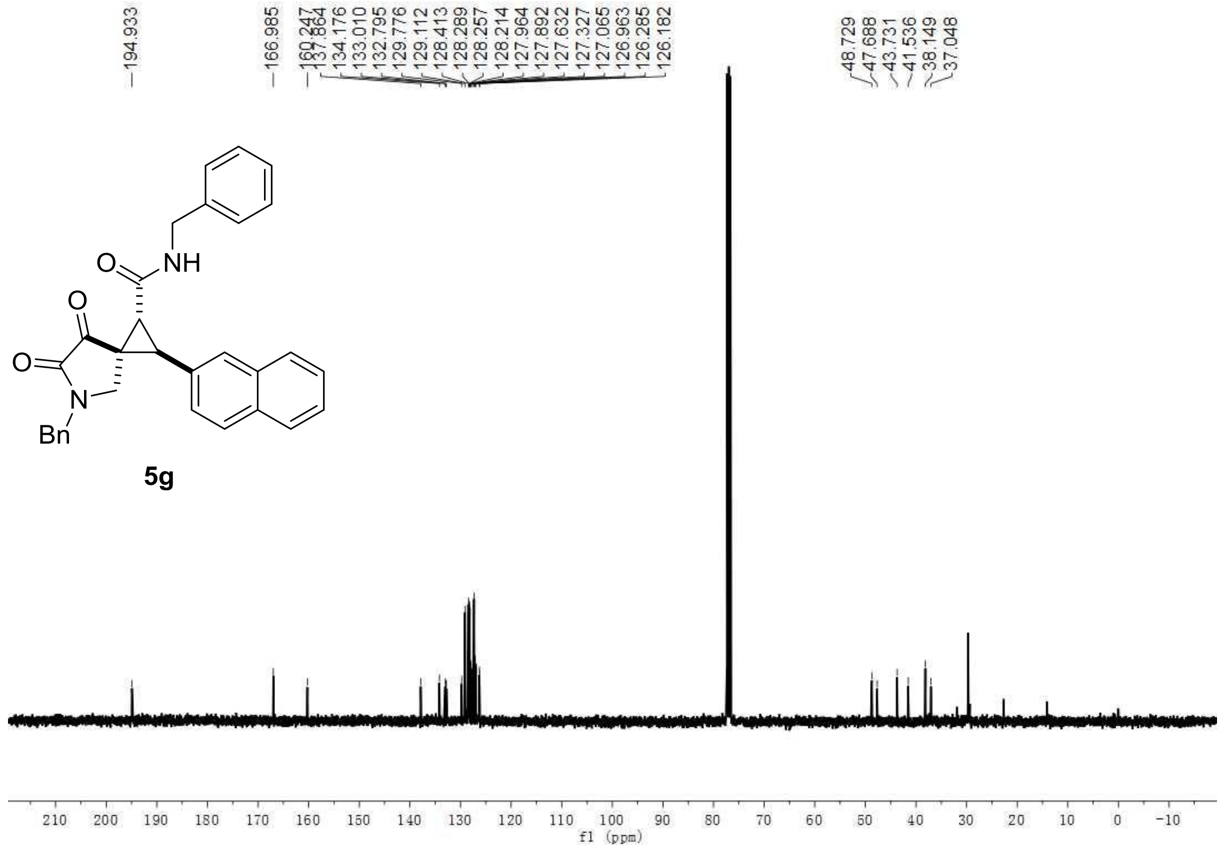
194.933
166.985
160.247
137.864
134.176
133.010
132.795
129.776
129.112
128.413
128.289
128.257
128.214
127.964
127.892
127.632
127.327
127.065
126.963
126.285
126.182
48.729
47.688
43.731
41.536
38.149
37.048
O
NH
O
O
N
Bn
5g
210 200 190 180 170 160 150 140 130 120 110 100 90 80 70 60 50 40 30 20 10 0 -10
f1 (ppm)

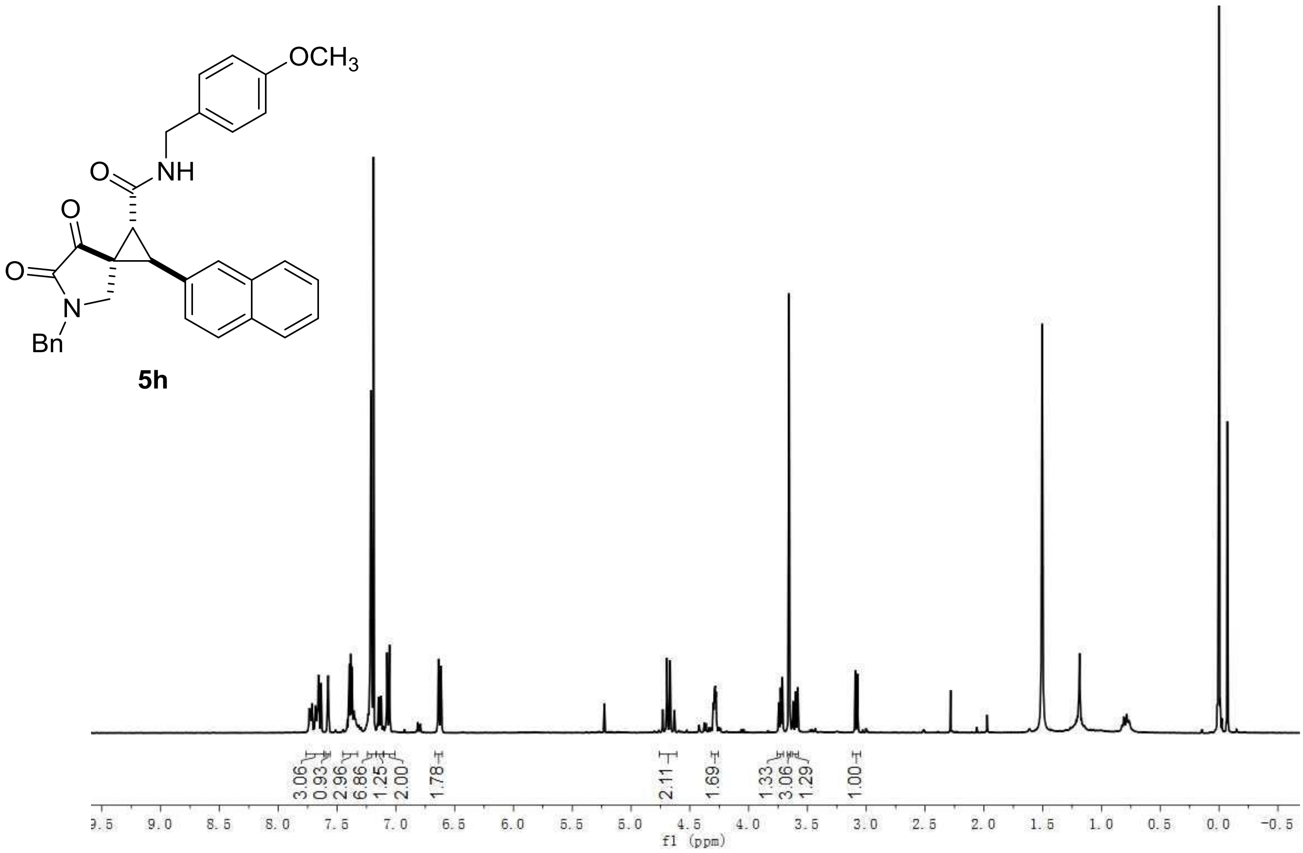
OCH3
NH
O
O
O
N
Bn
5h
3.06
0.93
2.96
6.86
1.25
2.00
1.78
2.11
1.69
1.33
3.06
1.29
1.00
9.5 9.0 8.5 8.0 7.5 7.0 6.5 6.0 5.5 5.0 4.5 4.0 3.5 3.0 2.5 2.0 1.5 1.0 0.5 0.0 -0.5
f1 (ppm)

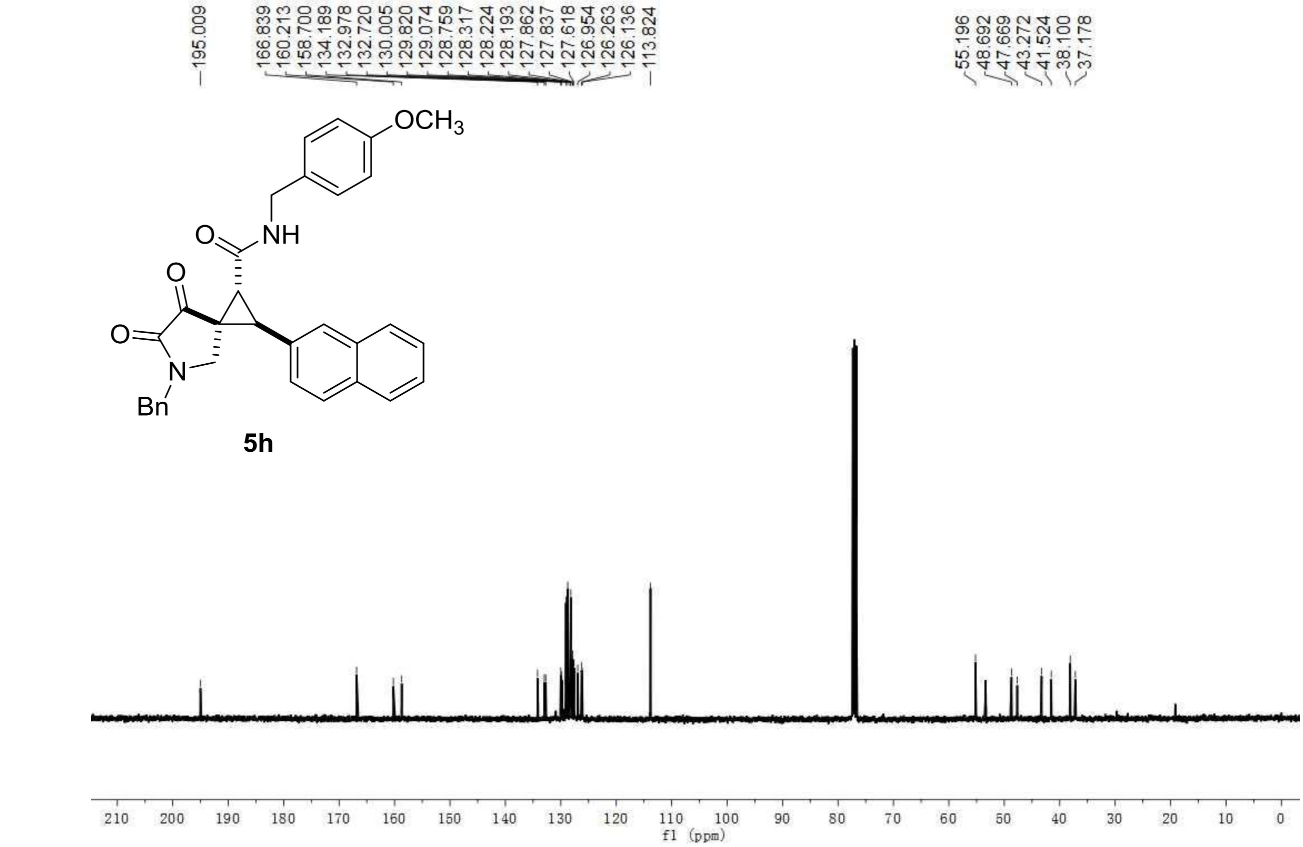
195.009
166.839
160.213
158.700
134.189
132.978
132.720
130.005
129.820
129.074
128.759
128.317
128.224
128.193
127.862
127.837
127.618
126.954
126.263
126.136
113.824
55.196
48.692
47.669
43.272
41.524
38.100
37.178
OCH3
NH
O
O
O
N
Bn
5h
210 200 190 180 170 160 150 140 130 120 110 100 90 80 70 60 50 40 30 20 10 0
f1 (ppm)

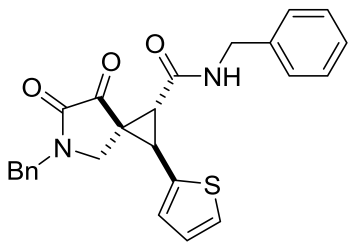
O
O
O
NH
N
Bn
S

5i

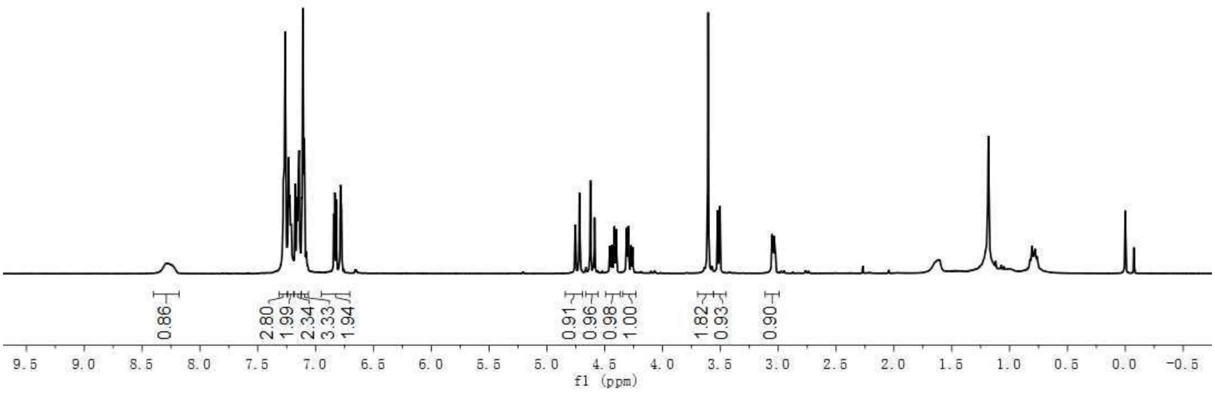
0.86
2.80
1.99
2.34
3.33
1.94
0.91
0.96
0.98
1.00
1.82
0.93
0.90
f1 (ppm)

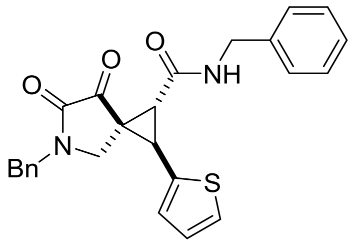
O
O
O
NH
N
Bn
S

5i

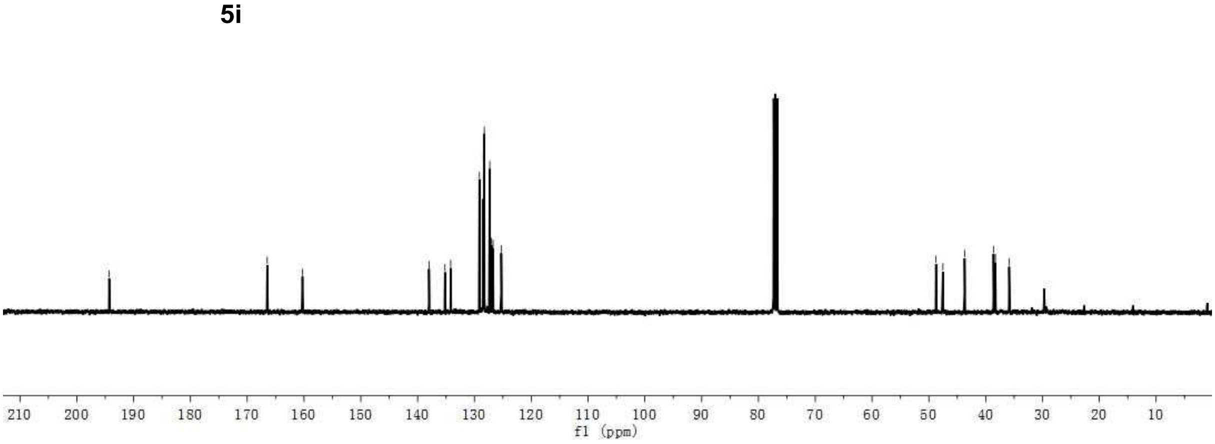
194.291
166.477
160.273
138.000
135.193
134.176
129.124
128.425
128.270
127.381
127.301
126.996
126.723
125.284
48.748
47.530
43.711
38.623
38.292
35.877
f1 (ppm)

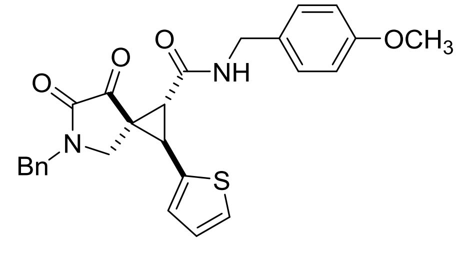
O
O
O
NH
OCH3
N
Bn
S

5j

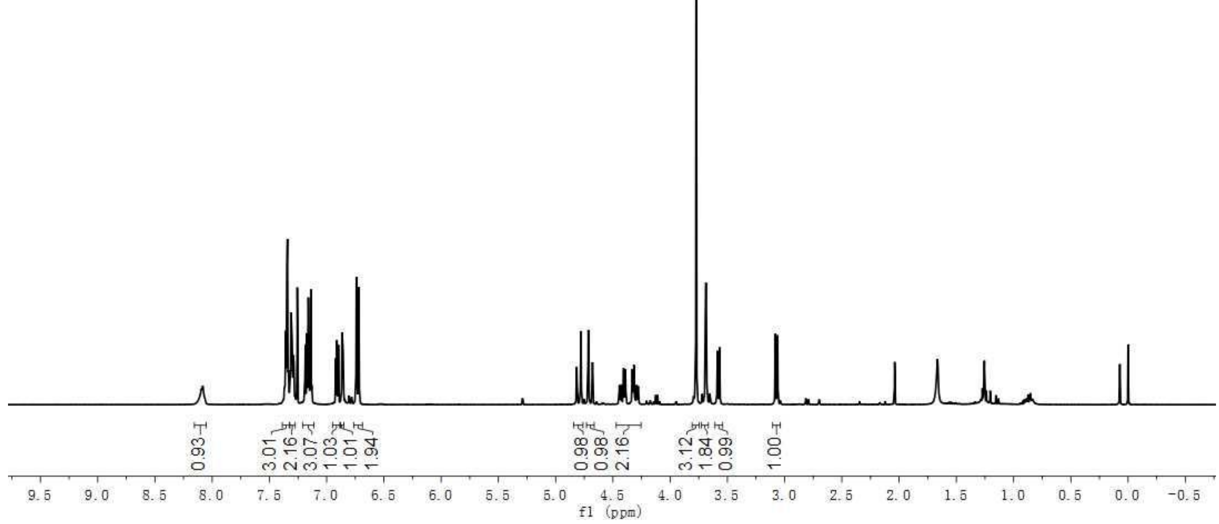
0.93
3.01
2.16
3.07
1.03
1.01
1.94
0.98
0.98
2.16
3.12
1.84
0.99
1.00
f1 (ppm)

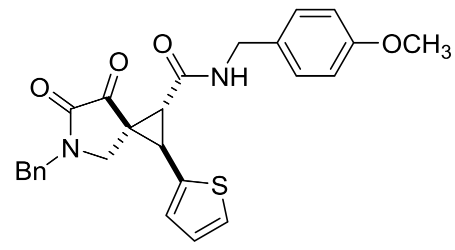
O
O
O
NH
OCH3
N
Bn
S

5j

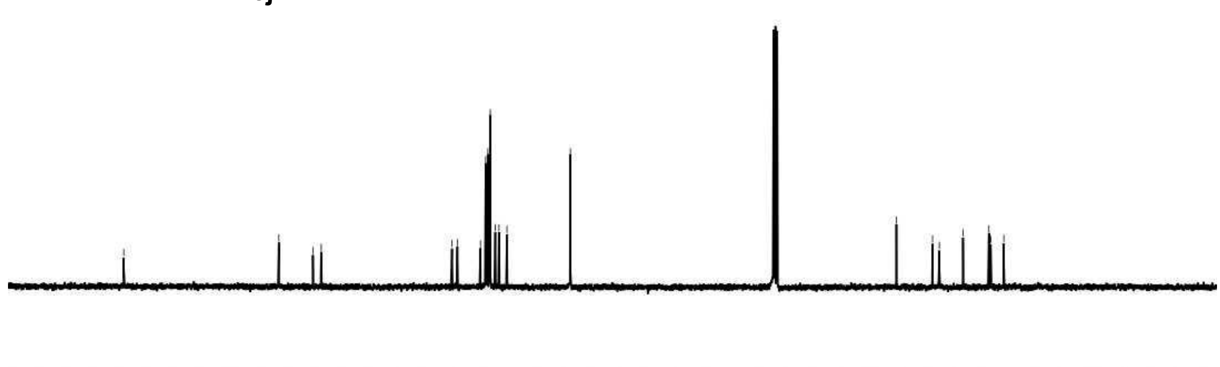
194.219
166.320
160.193
158.701
135.178
134.213
130.053
129.117
128.719
128.282
127.390
126.726
125.288
113.879
55.241
48.734
47.511
43.256
38.598
38.282
35.917
f1 (ppm)

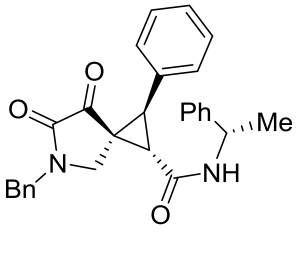
O
O
Ph
Me
N
NH
Bn
O

6a

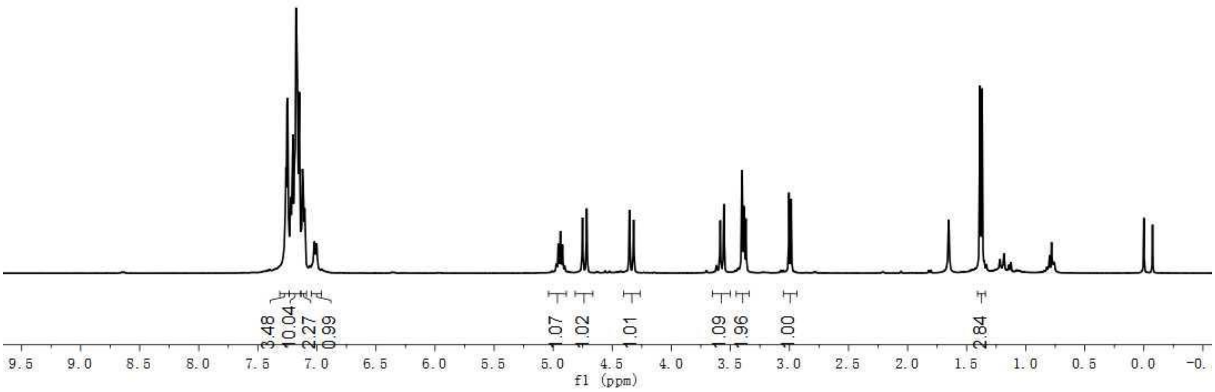
3.48
10.04
2.27
0.99
1.07
1.02
1.01
1.09
1.96
1.00
2.84
fl (ppm)

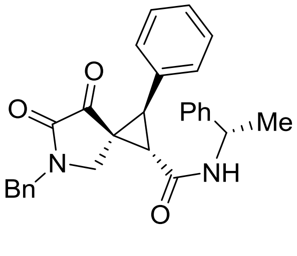
O
O
Ph
Me
N
NH
Bn
O

6a

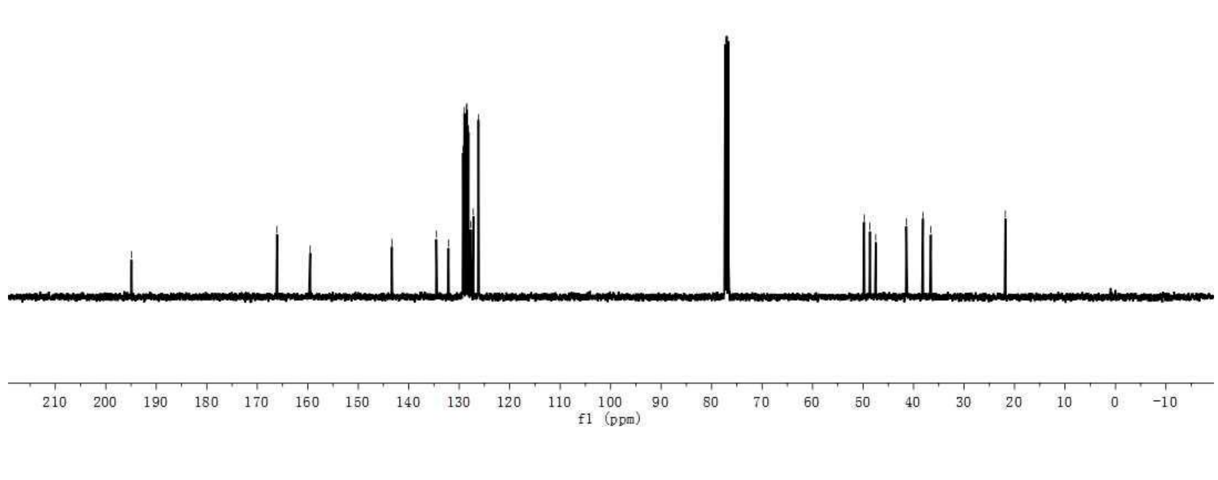
194.911
166.089
159.511
143.327
134.543
132.119
129.249
128.980
128.551
128.496
128.242
128.161
127.710
127.209
126.185
49.786
48.650
47.486
41.439
38.134
36.589
21.816
fl (ppm)

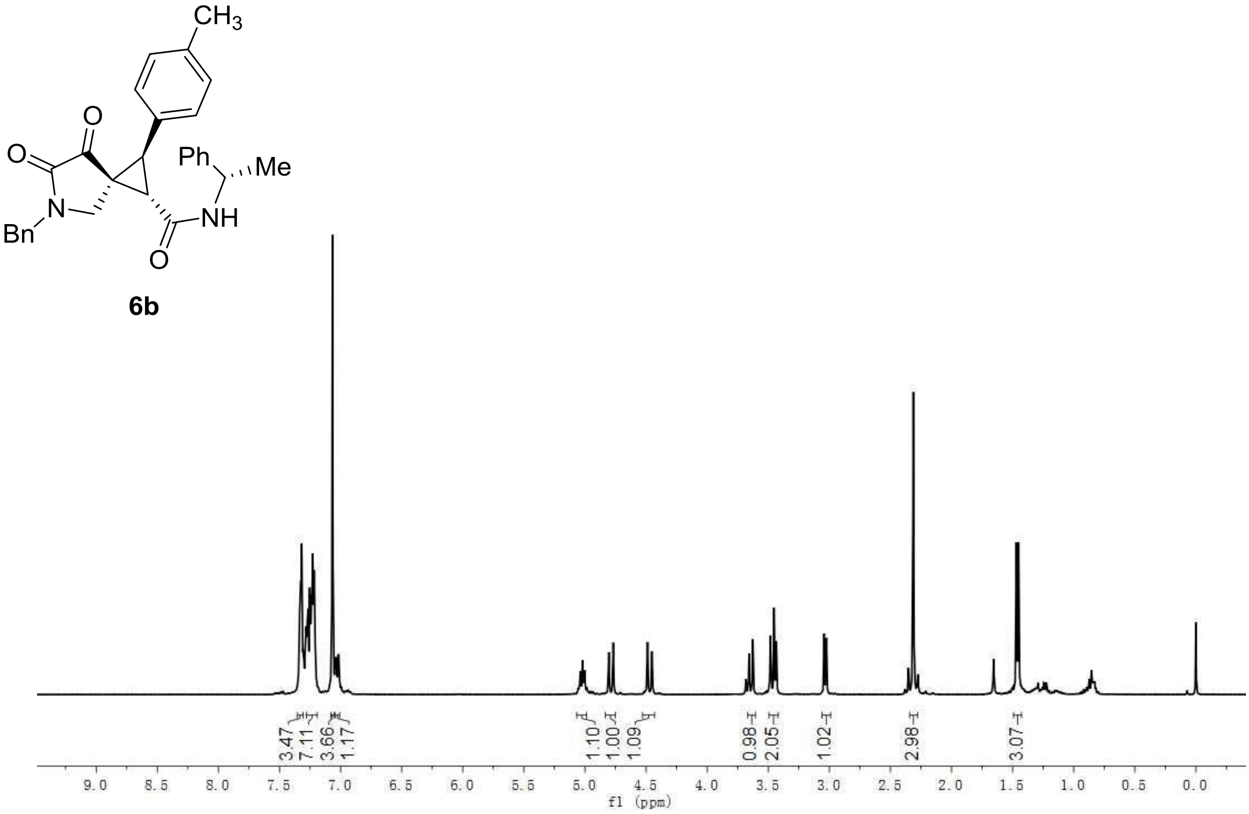
CH3
O
O
Ph
Me
N
NH
Bn
O
3.47
7.11
3.66
1.17
1.10
1.00
1.09
0.98
2.05
1.02
2.98
3.07
9.0 8.5 8.0 7.5 7.0 6.5 6.0 5.5 5.0 4.5 4.0 3.5 3.0 2.5 2.0 1.5 1.0 0.5 0.0
f1 (ppm)

6b

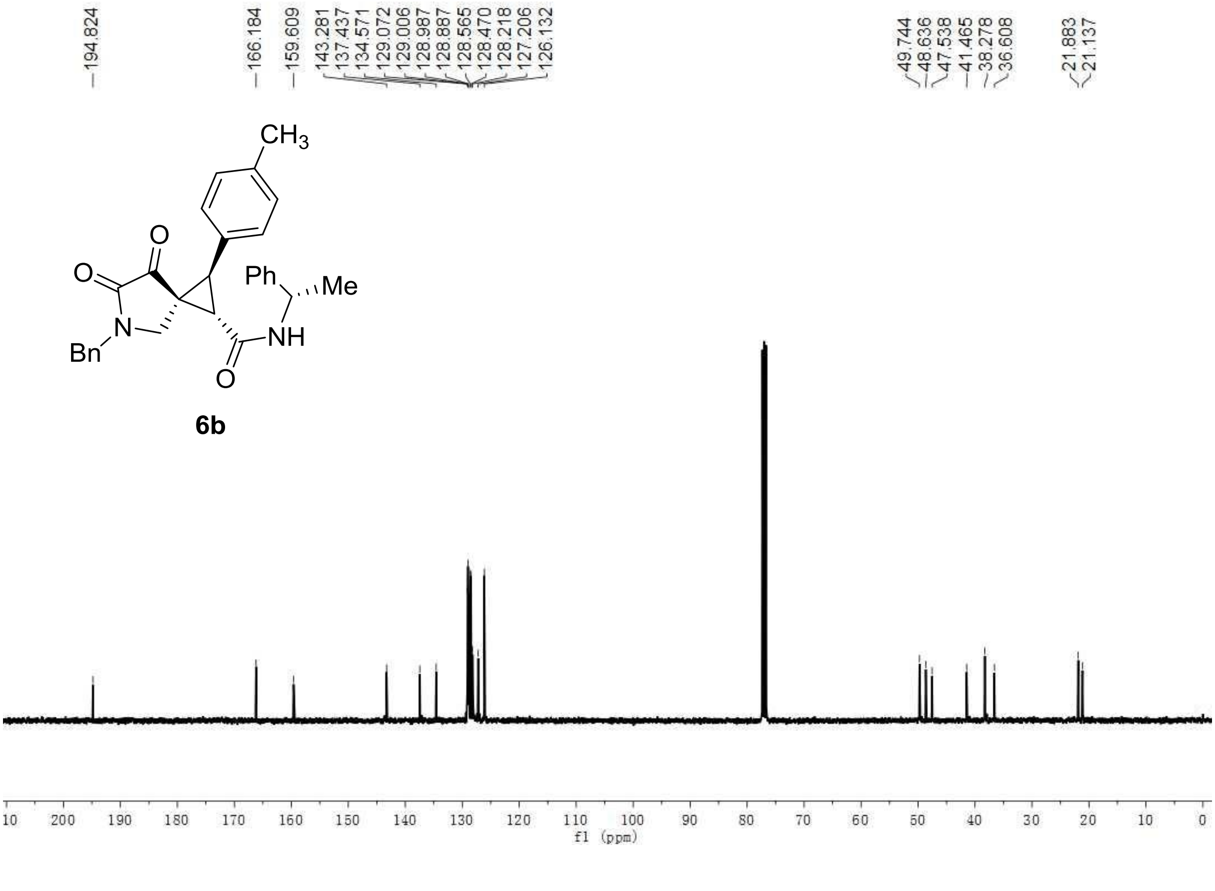
194.824
166.184
159.609
143.281
137.437
134.571
129.072
129.006
128.987
128.887
128.565
128.470
128.218
127.206
126.132
49.744
48.636
47.538
41.465
38.278
36.608
21.883
21.137
CH3
O
O
Ph
Me
N
NH
Bn
O
10 200 190 180 170 160 150 140 130 120 110 100 90 80 70 60 50 40 30 20 10 0
f1 (ppm)

6b

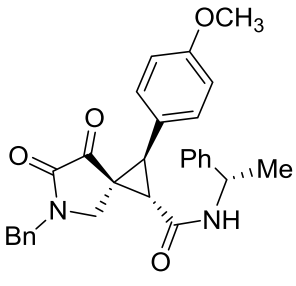

6c

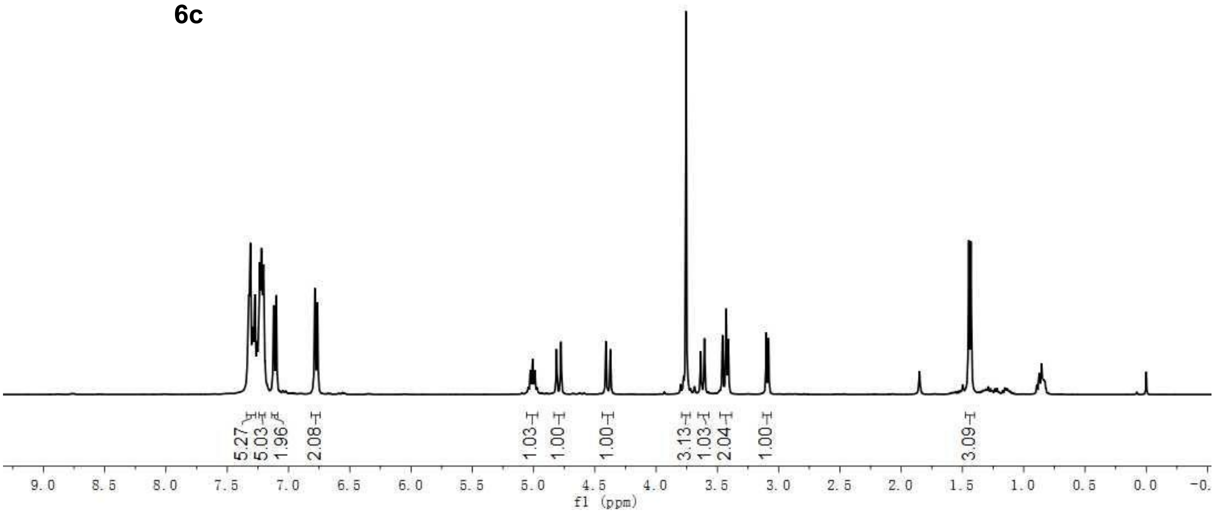

5.27
5.03
1.96
2.08
1.03
1.00
1.00
3.13
1.03
2.04
1.00
3.09
9.0 8.5 8.0 7.5 7.0 6.5 6.0 5.5 5.0 4.5 4.0 3.5 3.0 2.5 2.0 1.5 1.0 0.5 0.0
f1 (ppm)

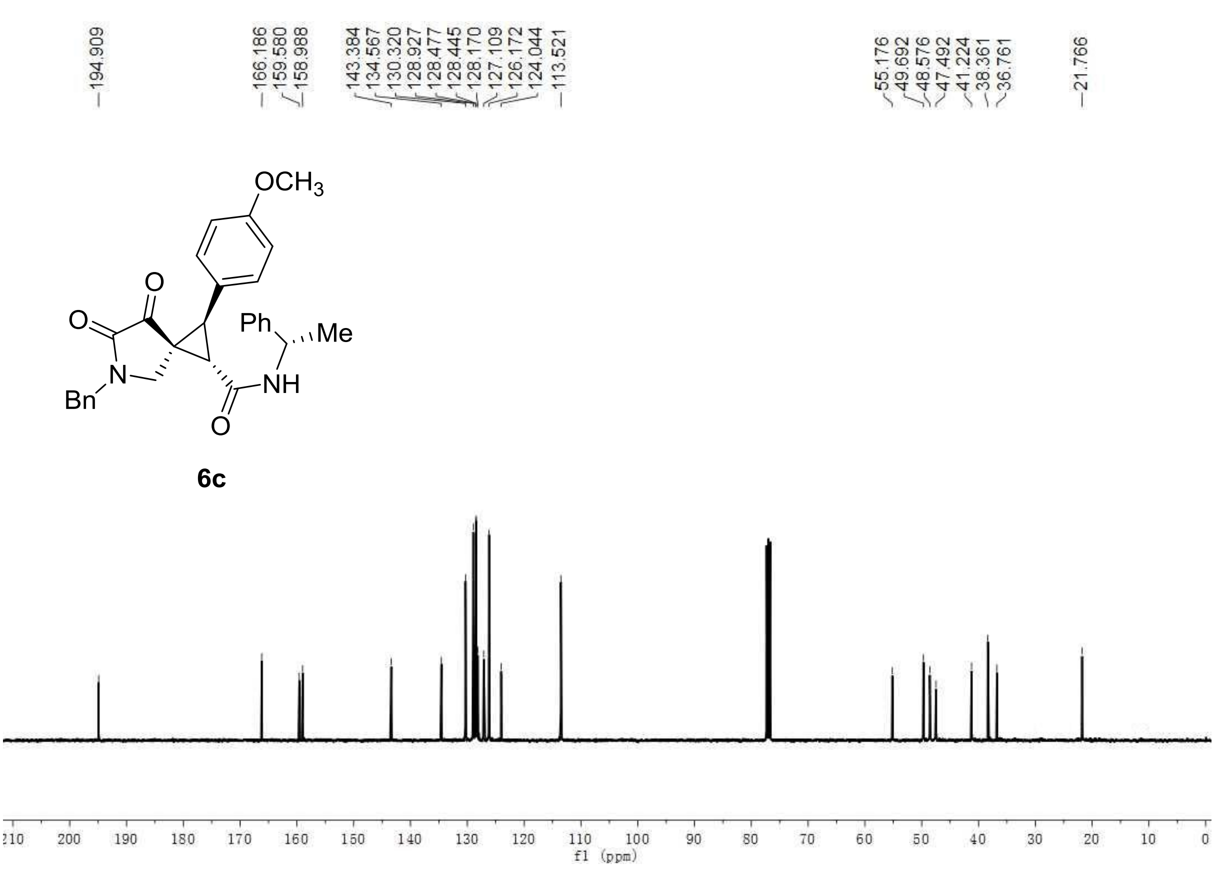

194.909
166.186
159.580
158.988
143.384
134.567
130.320
128.927
128.477
128.445
128.170
127.109
126.172
124.044
113.521
55.176
49.692
48.576
47.492
41.224
38.361
36.761
21.766
210 200 190 180 170 160 150 140 130 120 110 100 90 80 70 60 50 40 30 20 10 0
f1 (ppm)

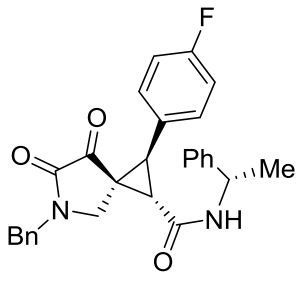
F
O
O
Ph
Me
N
NH
Bn
O

6d

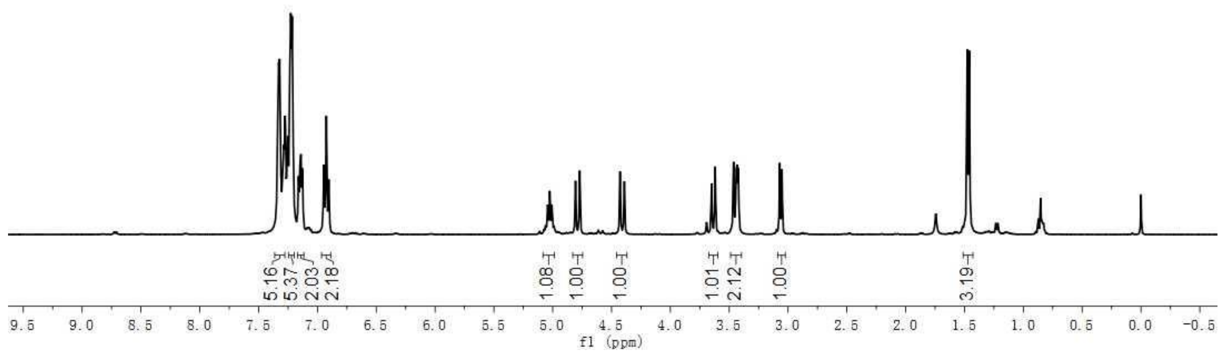
5.16
5.37
2.03
2.18
1.08
1.00
1.00
1.01
2.12
1.00
3.19
f1 (ppm)

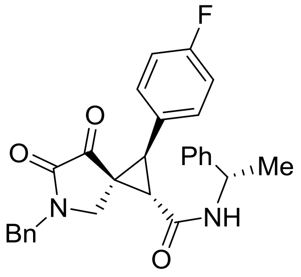
F
O
O
Ph
Me
N
NH
Bn
O

6d

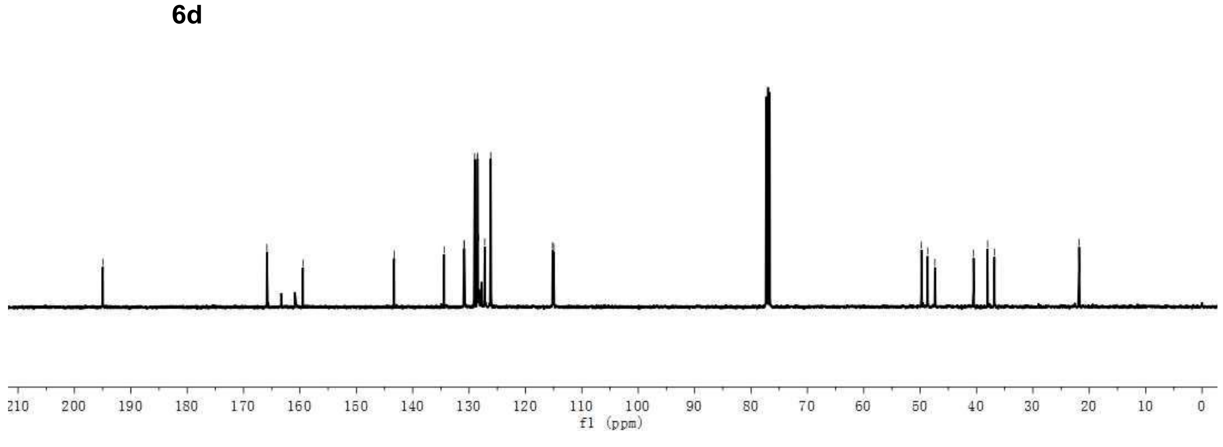
194.960
165.876
159.469
143.298
134.427
130.917
130.835
129.007
128.542
128.468
128.301
127.225
126.162
115.199
114.984
49.785
48.668
47.384
40.523
38.048
36.826
21.805
f1 (ppm)

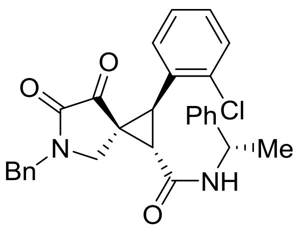
O
O
Ph
Cl
Me
N
Bn
NH
O

6e

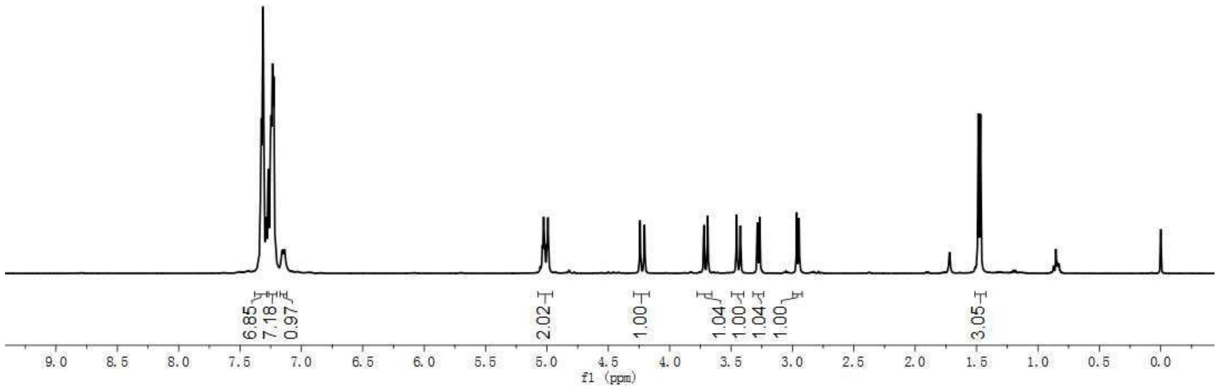
6.85
7.18
0.97
2.02
1.00
1.04
1.00
1.04
1.00
3.05
f1 (ppm)

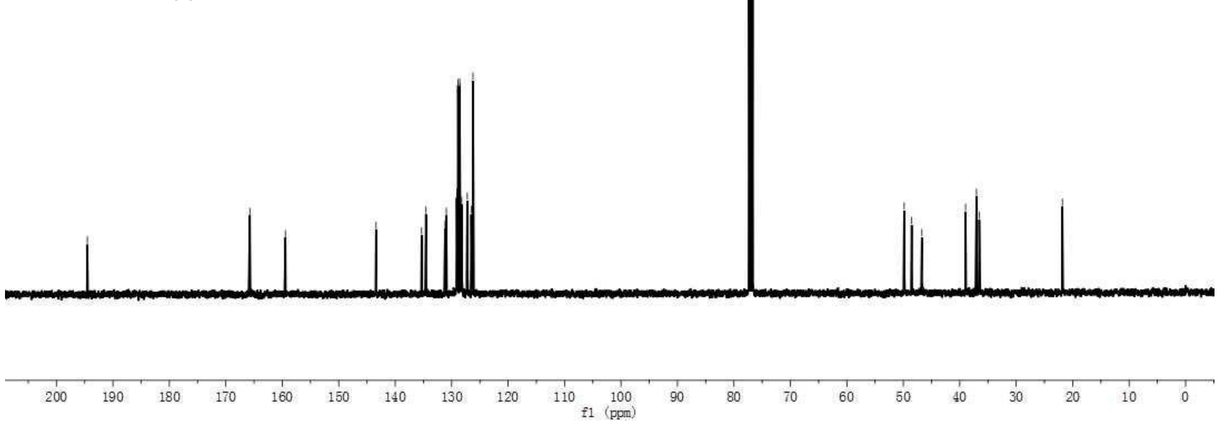
194.534
165.740
159.447
143.390
135.321
134.546
131.163
130.945
129.092
129.068
128.866
128.576
128.478
128.221
127.233
126.526
126.223
49.847
48.509
48.477
46.714
38.970
37.036
36.538
21.821
f1 (ppm)

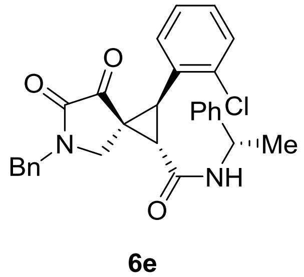
O
O
Ph
Cl
Me
N
Bn
NH
O

6e

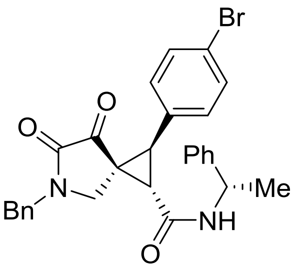
Br
O
O
N
Bn
Ph
Me
NH
O

6f

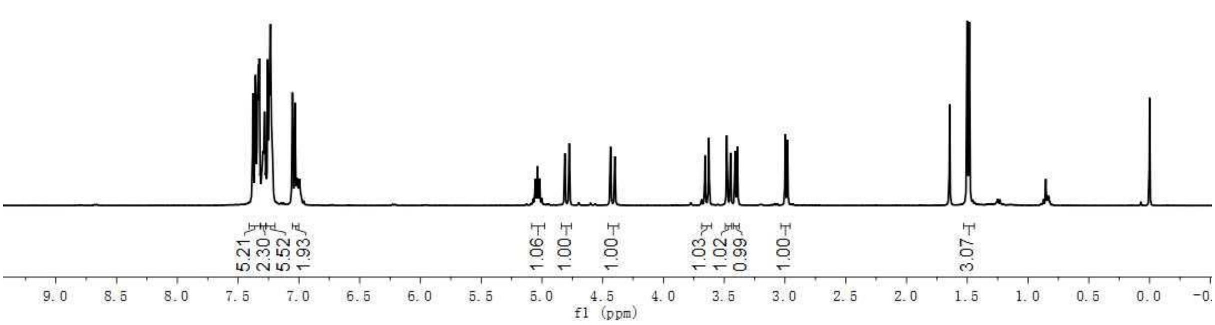
5.21
2.30
5.52
1.93
1.06
1.00
1.00
1.03
1.02
0.99
1.00
3.07
9.0 8.5 8.0 7.5 7.0 6.5 6.0 5.5 5.0 4.5 4.0 3.5 3.0 2.5 2.0 1.5 1.0 0.5 0.0 -0.
f1 (ppm)

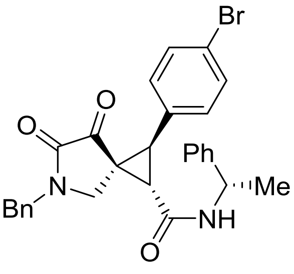
Br
O
O
N
Bn
Ph
Me
NH
O

6f

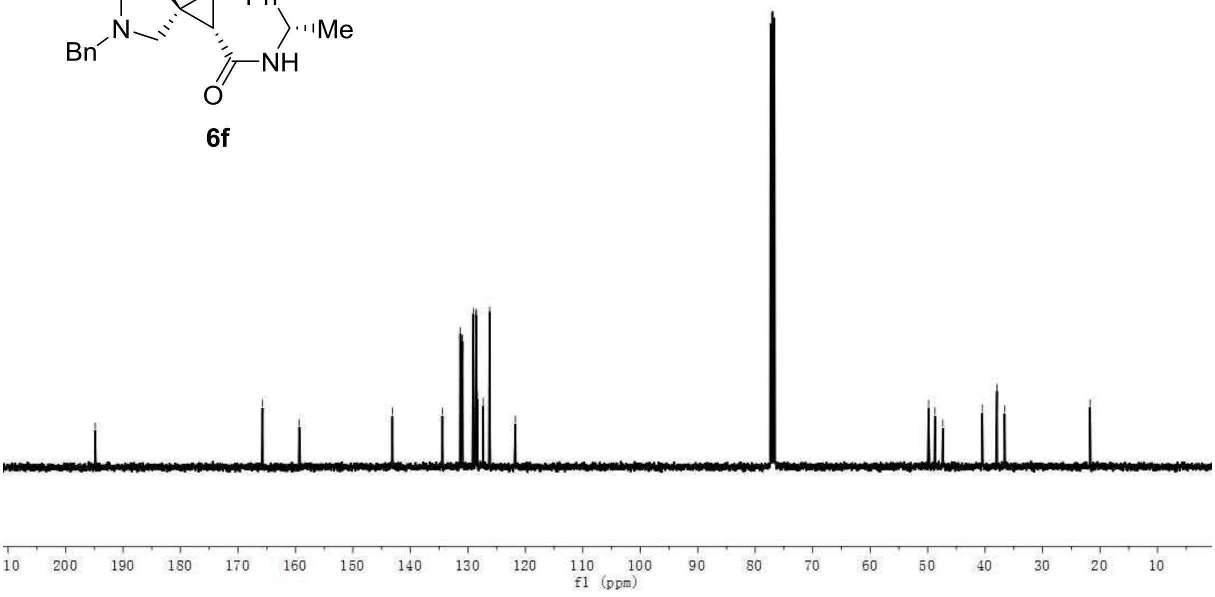
194.844
165.747
159.345
143.142
134.432
131.310
131.104
130.944
129.035
128.607
128.532
128.346
127.337
126.207
121.748
49.826
48.717
47.353
40.500
37.936
36.613
21.750
10 200 190 180 170 160 150 140 130 120 110 100 90 80 70 60 50 40 30 20 10
f1 (ppm)

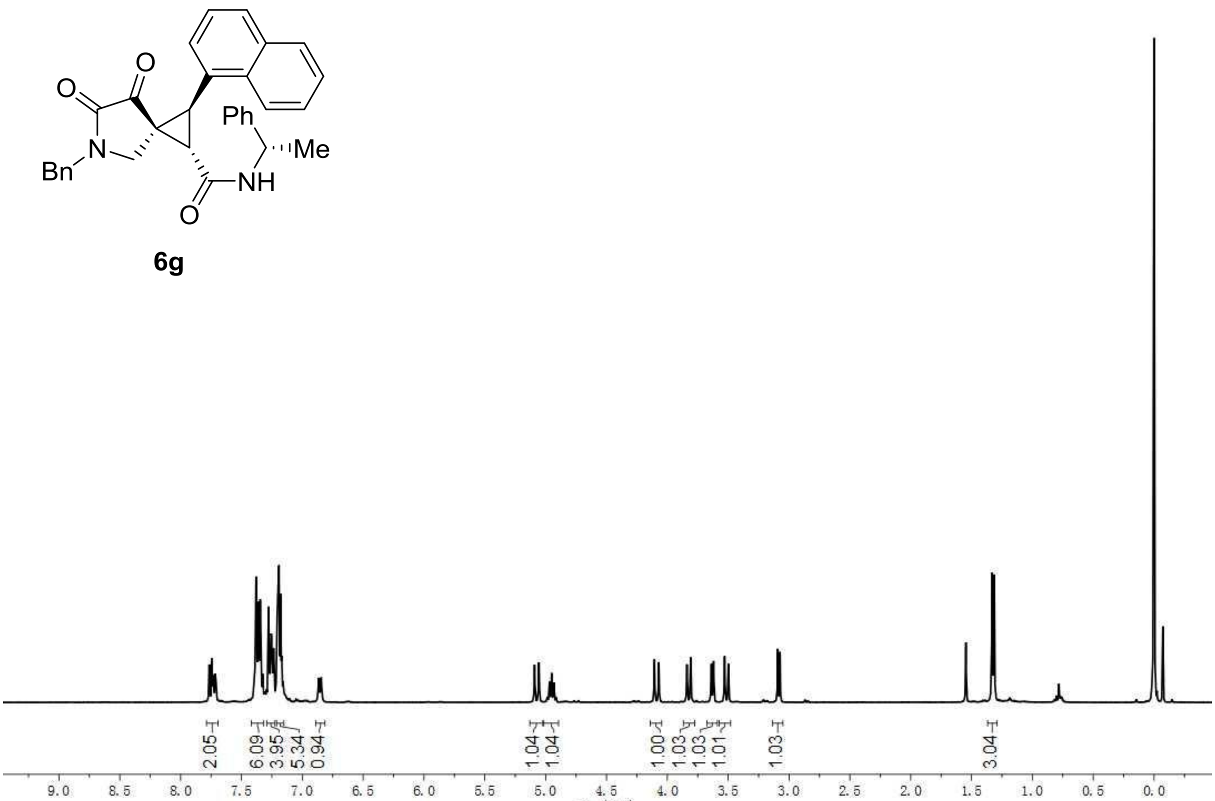
O
O
Ph
Me
N
Bn
NH
O
6g
2.05
6.09
3.95
5.34
0.94
1.04
1.04
1.00
1.03
1.03
1.01
1.03
3.04
9.0 8.5 8.0 7.5 7.0 6.5 6.0 5.5 5.0 4.5 4.0 3.5 3.0 2.5 2.0 1.5 1.0 0.5 0.0
f1 (ppm)

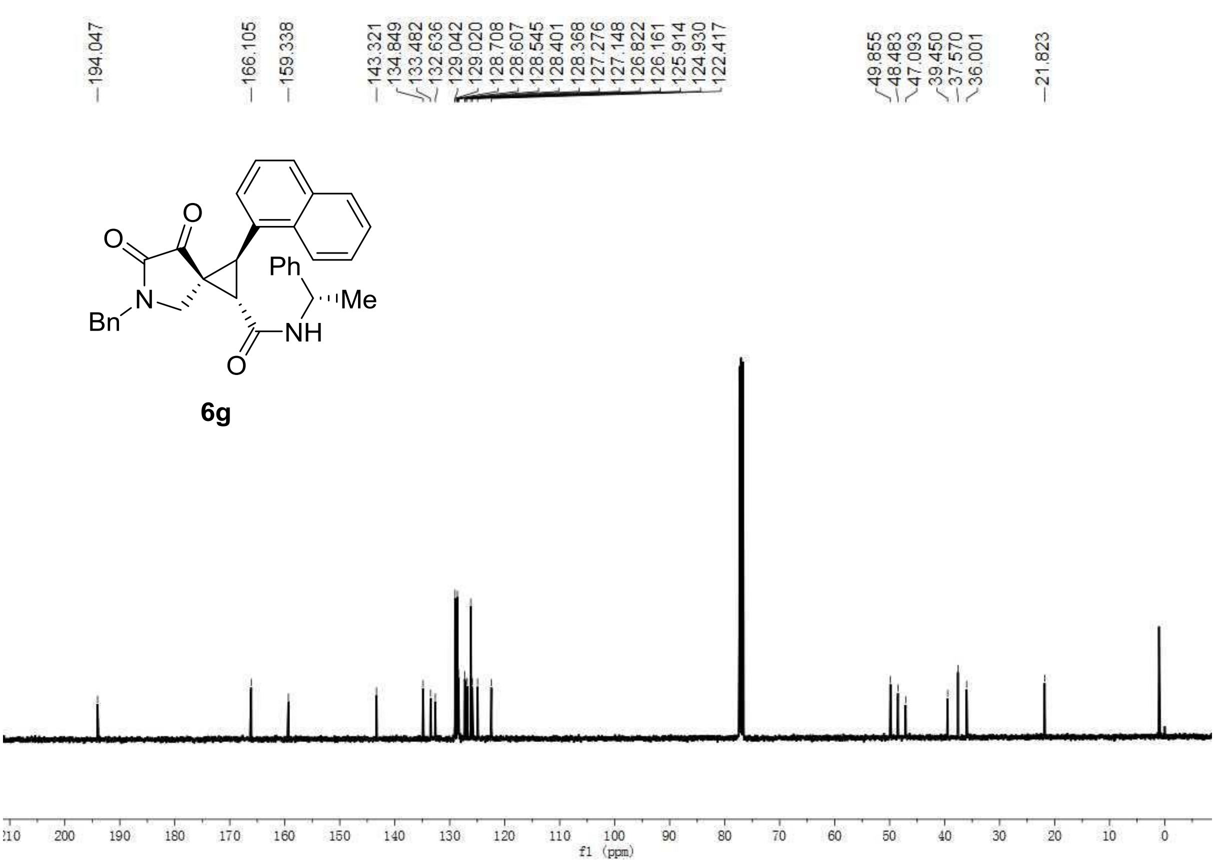
194.047
166.105
159.338
143.321
134.849
133.482
132.636
129.042
129.020
128.708
128.607
128.545
128.401
128.368
127.276
127.148
126.822
126.161
125.914
124.930
122.417
49.855
48.483
47.093
39.450
37.570
36.001
21.823
O
O
Ph
Me
N
Bn
NH
O
6g
210 200 190 180 170 160 150 140 130 120 110 100 90 80 70 60 50 40 30 20 10 0
f1 (ppm)

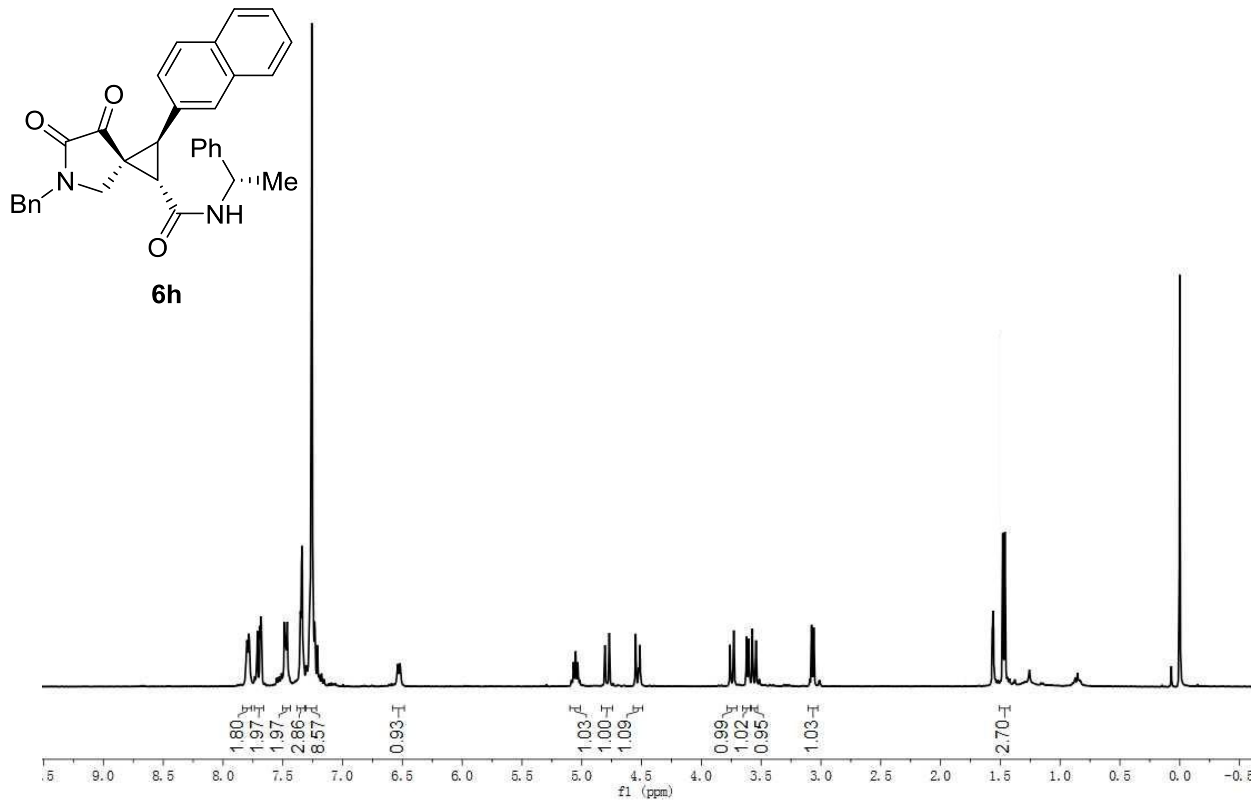

O
O
Ph
N
Me
Bn
NH
O
6h
1.80
1.97
1.97
2.86
8.57
0.93
1.03
1.00
1.09
0.99
1.02
0.95
1.03
2.70
9.0
8.5
8.0
7.5
7.0
6.5
6.0
5.5
5.0
4.5
4.0
3.5
3.0
2.5
2.0
1.5
1.0
0.5
0.0
f1 (ppm)

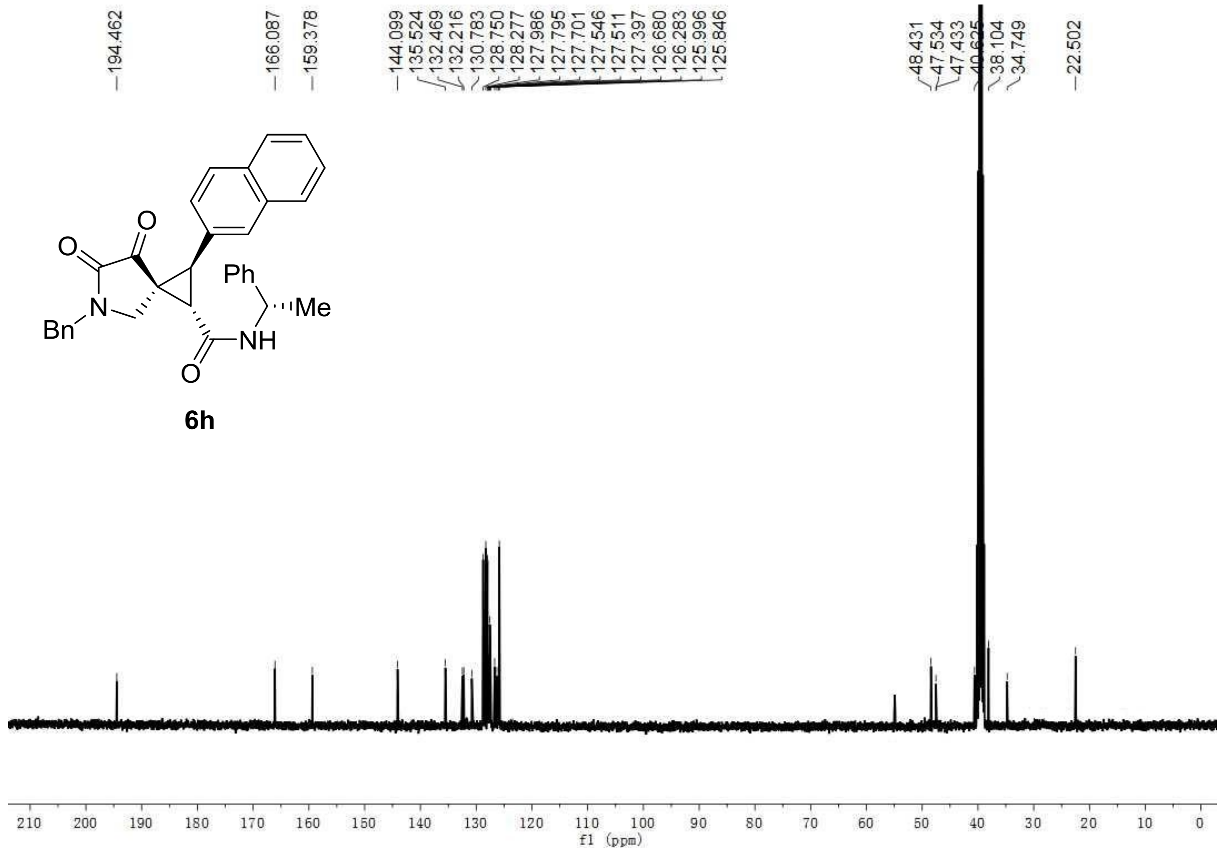

194.462
166.087
159.378
144.099
135.524
132.469
132.216
130.783
128.750
128.277
127.986
127.795
127.701
127.546
127.511
127.397
126.680
126.283
125.996
125.846
48.431
47.534
47.433
40.625
38.104
34.749
22.502
O
O
Ph
N
Me
Bn
NH
O
6h
210
200
190
180
170
160
150
140
130
120
110
100
90
80
70
60
50
40
30
20
10
0
f1 (ppm)

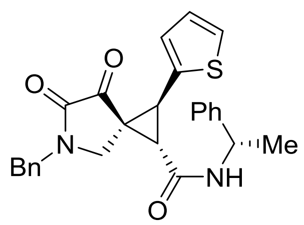

O
O
S
Ph
Me
N
Bn
NH
O

6i

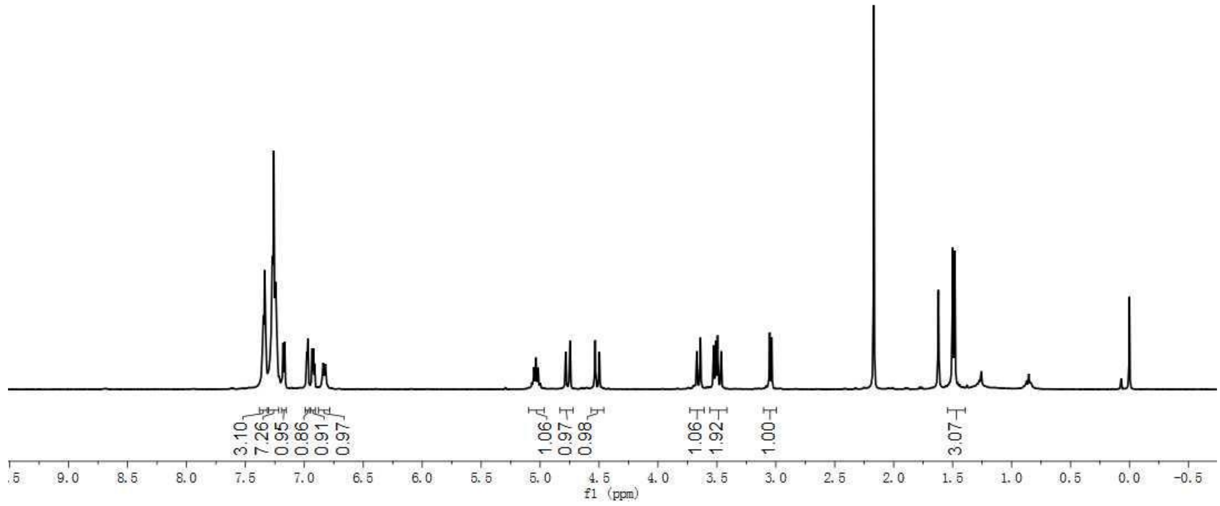

3.10
7.26
0.95
0.86
0.91
0.97
1.06
0.97
0.98
1.06
1.92
1.00
3.07
f1 (ppm)

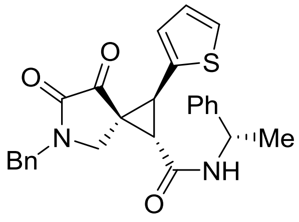

O
O
S
Ph
Me
N
Bn
NH
O

6i

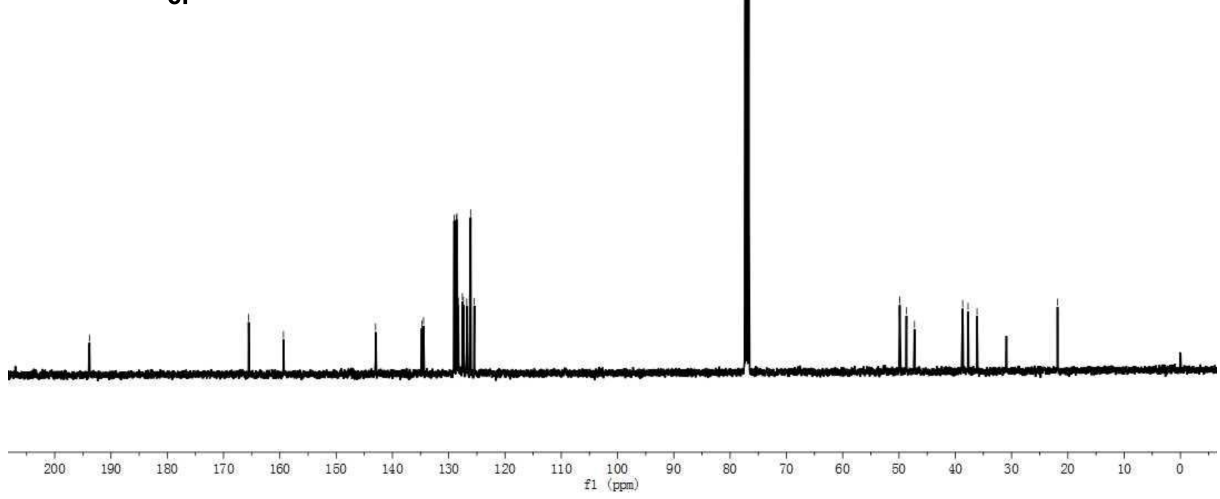

193.826
165.540
159.365
142.989
134.781
134.473
129.017
128.674
128.537
128.295
127.575
127.396
126.797
126.109
125.450
49.875
48.681
47.247
38.684
37.728
36.134
21.827
f1 (ppm)
